# *Helicobacter pylori*-Induced Angiopoietin-Like 4 Promotes Gastric Bacterial Colonization and Gastritis

**DOI:** 10.34133/research.0409

**Published:** 2024-07-17

**Authors:** Rui Xie, Nan You, Wan-Yan Chen, Peng Zhu, Pan Wang, Yi-Pin Lv, Geng-Yu Yue, Xiao-Lin Xu, Jiang-Bo Wu, Jing-Yu Xu, Si-Xu Liu, Mu-Han Lü, Sheng-Qian Yang, Ping Cheng, Fang-Yuan Mao, Yong-Sheng Teng, Liu-Sheng Peng, Jin-Yu Zhang, Ya-Ling Liao, Shi-Ming Yang, Yong-Liang Zhao, Weisan Chen, Quan-Ming Zou, Yuan Zhuang

**Affiliations:** ^1^ Department ofEndoscopy and Digestive System, Guizhou Provincial People’s Hospital, Guiyang, China.; ^2^ Department of Hepatobiliary Surgery, XinQiao Hospital, Third Military Medical University, Chongqing, China.; ^3^ National Engineering Research Center of Immunological Products, Department of Microbiology and Biochemical Pharmacy, College of Pharmacy and Laboratory Medicine, Third Military Medical University, Chongqing, China.; ^4^ Department of Gastroenterology, Suining First People’s Hospital, Suining, Sichuan, China.; ^5^ Department of Infection, The General Hospital of Western Theater Command, Chengdu, Sichuan, China.; ^6^ Department of Gastroenterology, Affiliated Hospital of Southwest Medical University, Luzhou, Sichuan, China.; ^7^ Chongqing Engineering Research Center for Pharmacodynamics Evaluation, Department of Pharmaceutics, College of Pharmacy and Laboratory Medicine, Third Military Medical University, Chongqing, China.; ^8^ Department of Gastroenterology, XinQiao Hospital, Third Military Medical University, Chongqing, China.; ^9^ Department of General Surgery and Center of Minimal Invasive Gastrointestinal Surgery, Southwest Hospital, Third Military Medical University, Chongqing, China.; ^10^ La Trobe Institute of Molecular Science, La Trobe University, Bundoora, Victoria 3085, Australia.; ^11^ State Key Laboratory of Trauma and Chemical Poisoning, Chongqing, China.

## Abstract

*Helicobacter pylori* infection is characterized as progressive processes of bacterial persistence and chronic gastritis with features of infiltration of mononuclear cells more than granulocytes in gastric mucosa. Angiopoietin-like 4 (ANGPTL4) is considered a double-edged sword in inflammation-associated diseases, but its function and clinical relevance in *H. pylori*-associated pathology are unknown. Here, we demonstrate both pro-colonization and pro-inflammation roles of ANGPTL4 in *H. pylori* infection. Increased ANGPTL4 in the infected gastric mucosa was produced from gastric epithelial cells (GECs) synergistically induced by *H. pylori* and IL-17A in a *cagA*-dependent manner. Human gastric ANGPTL4 correlated with *H. pylori* colonization and the severity of gastritis, and mouse ANGPTL4 from non-bone marrow-derived cells promoted bacteria colonization and inflammation. Importantly, *H. pylori* colonization and inflammation were attenuated in *Il17a*^−/−^, *Angptl4*^−/−^, and *Il17a*^−/−^*Angptl4*^−/−^ mice. Mechanistically, ANGPTL4 bound to integrin αV (ITGAV) on GECs to suppress CXCL1 production by inhibiting ERK, leading to decreased gastric influx of neutrophils, thereby promoting *H. pylori* colonization; ANGPTL4 also bound to ITGAV on monocytes to promote CCL5 production by activating PI3K–AKT–NF-κB, resulting in increased gastric influx of regulatory CD4^+^ T cells (T_regs_) via CCL5–CCR4-dependent migration. In turn, ANGPTL4 induced T_reg_ proliferation by binding to ITGAV to activate PI3K–AKT–NF-κB, promoting *H. pylori*-associated gastritis. Overall, we propose a model in which ANGPTL4 collectively ensures *H. pylori* persistence and promotes gastritis. Efforts to inhibit ANGPTL4-associated pathway may prove valuable strategies in treating *H. pylori* infection.

## Introduction

*Helicobacter pylori*, as the human gastric colonizing gram-negative bacterium, can infect nearly about 50% of the world’s people [1,2]. *H. pylori* infection frequently causes chronic gastritis featuring more mononuclear cell infiltration than granulocytes within the gastric mucosa, which may contribute to not only progressive inflammation but also bacterial persistence [3,4]. Although the development of chronic gastritis caused by *H. pylori* infection is previously unknown, it is often believed that *H. pylori* infection-induced altered cell infiltration within the gastric mucosa is an important contributing factor [5,6]. As the first-contacted cells infected by *H. pylori* within the gastric mucosa, gastric epithelial cells (GECs) are believed to be the major effector cells modulated by *H. pylori* to secrete proinflammatory molecules that regulate a biased inflammatory response [7,8]. Moreover, the biological activities of several inflammatory factors, including matrix metalloproteinases and matricellular proteins, could also change immune cell infiltration shown in *H. pylori* infection [9,10]. However, potential factors that induce largely mononuclear cell infiltration, rather than granulocytes during *H. pylori* infection, are not yet identified.

Angiopoietin-like (ANGPTL) proteins, as angiogenic-regulating secreted proteins, are almost structurally similar to the ANGPTs [11,12]. ANGPTL4, one member of the ANGPTL proteins, does not bind to ANGPT receptor TIE1/2, suggesting distinct functions of ANGPTL4 by different mechanisms [13]. ANGPTL4 exerts biological functions in a wide array, including angiogenesis regulation and chronic inflammation promotion [14,15], and full-length ANGPTL4 (flANGPTL4) can undergo proteolytic processing at its linker regions to release the N-terminal as well as the C-terminal portions to form proteins of nANGPTL4 and cANGPTL4, respectively [16]. ANGPTL4 (or specific domains of the protein) can play roles of protecting against cancer or promoting tumorigenesis [17]. In primary tumors, cANGPTL4 promotes tumor progression; in contrast, nANGPTL4 inhibits tumor metastasis in a postsurgical metastasis model [18]. In respiratory infection, ANGPTL4 contributes to pathology during influenza pneumonia as well as in secondary pneumococcal pneumonia [19,20]. To date, in either humans or mice, almost nothing is known about the functional mechanism as well as the clinical relevance of ANGPTL4 in gastric infection induced by *H. pylori*. In the current study, we have, for the first time, demonstrated a pathological role for ANGPTL4 in gastric *H. pylori* persistence and *H. pylori* infection-induced clinical gastritis.

## Results

### ANGPTL4 is increased in gastric mucosa of *H. pylori*-infected patients and mice

**Fig. 1.**
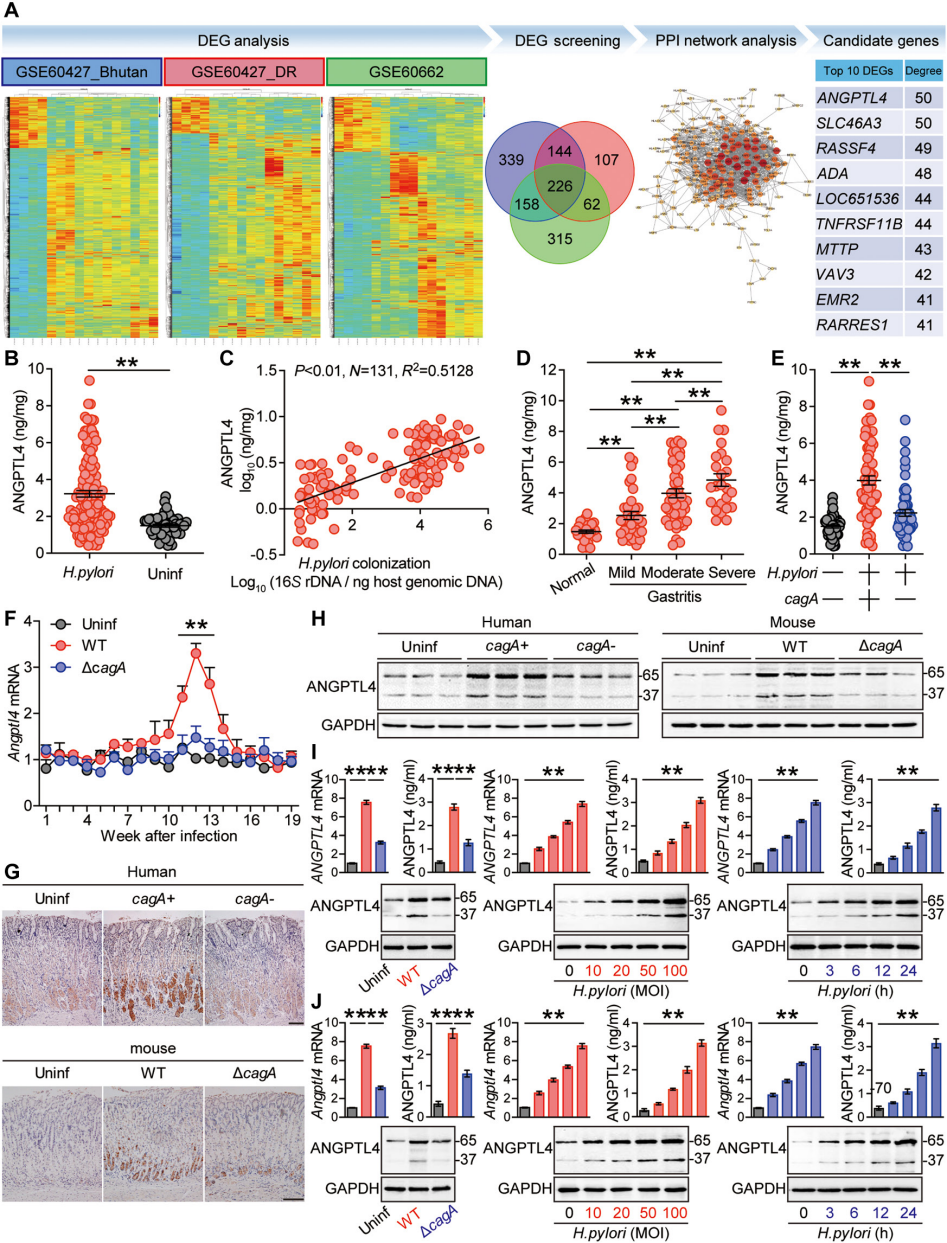
ANGPTL4 is increased in gastric mucosa of *H. pylori*-infected patients and mice. (A) Analyses of the whole-transcriptome sequencing datasets and the 3 cohorts (GSE60427_Bhutan, GSE60427_DR, and GSE60662) from 2 GEO datasets. Hierarchical clustering heatmaps of significant DEGs are shown in DEG analysis. Overlapping 3 cohorts screened and identified 226 significantly up-regulated DEGs. PPI network analysis of these 226 DEGs was performed, and the DEGs with the significance levels (*P* < 0.01 and correlation > 0.8) are shown in PPI network, and the top 10 DEGs as candidate genes are shown in the side table. (B) ANGPTL4 protein amounts in gastric mucosa of *H. pylori*-infected patients (*n* = 131) and uninfected donors (*n* = 50) were compared. (C) The correlation between ANGPTL4 protein amount and *H. pylori* colonization in gastric mucosa of *H. pylori*-infected patients was analyzed. (D) ANGPTL4 protein amounts in gastric mucosa of *H. pylori*-infected patients with mild (*n* = 34), moderate (*n* = 45), and severe inflammation (*n* = 24) and with normal gastric histopathology (*n* = 28) were compared. (E) ANGPTL4 protein amounts in gastric mucosa of *cagA*^+^ *H. pylori*-infected (*n* = 74), *cagA*^−^ *H. pylori*-infected (*n* = 57), and uninfected donors (*n* = 50) were compared. (F) Dynamic changes of *Angptl4* gene expression in gastric mucosa of WT *H. pylori*-infected, *ΔcagA*-infected, and uninfected mice (*n* = 5 per group per time point). (G and H) ANGPTL4 protein expression in gastric mucosa of *cagA*^+^ *H. pylori*-infected, *cagA*^−^ *H. pylori*-infected, and uninfected donors or in gastric mucosa of WT *H. pylori*-infected, *ΔcagA*-infected, and uninfected mice at 12 weeks p.i. was revealed by immunohistochemical staining (G) and Western blot (H), respectively. Scale bars, 100 μm. (I and J) *ANGPTL4*/*Angptl4* gene and ANGPTL4 protein expressions in/from human/mouse primary gastric mucosa of uninfected donors or mice infected with WT *H. pylori* or *ΔcagA* were analyzed ex vivo by real-time PCR, ELISA, and Western blot (*n* = 5). Data are representative of 2 independent experiments. Data are shown as mean ± SEM and analyzed by Student’s *t* test, Mann–Whitney *U* test, and one-way ANOVA. Western blot results are run in parallel and contemporaneously. *P < 0.05, **P < 0.01 for groups connected by horizontal lines or compared with uninfected mice.

To investigate the involvement of ANGPTL4 in *H. pylori*-associated pathology, we first enrolled 2 public datasets of clinic samples of gastritis associated with *H. pylori* infection from the Gene Expression Omnibus (GEO) (GSE60427 and GSE60662). In addition, we performed whole-transcriptome sequencing on 12 cases of “Bhutan gastritis” and 4 cases of “Bhutan normal” from “Series Matrix” of GSE60427 (this dataset was defined as GSE60427_Bhutan), as well as on 12 cases of “DR gastritis” and 4 cases of “DR normal” from “Series Matrix” of GSE60427 (this dataset was defined as GSE60427_DR), and identified 2,596 and 1,762 overlapping differentially expressed genes (DEGs), respectively, using substantial levels (*P* < 0.05 and fold change <0.5 or >2) (Fig. 1A). We also performed such whole-transcriptome sequencing on 12 cases of “gastritis” and 4 cases of “control” from “Series Matrix” of GSE60662, and identified 1,888 overlapping DEGs using the same significance levels described above (Fig. 1A). We further obtained 226 significantly up-regulated DEGs (*P* < 0.05 and fold change > 2) by overlapping the above 3 cohorts (GSE60427_Bhutan, GSE60427_DR, and GSE60662) (Fig. 1A). To identify the genes with significant clinical value for *H. pylori*-associated gastritis, we performed protein–protein interaction (PPI) network analysis of these 226 DEGs, and identified top 10 DEGs using significance levels (*P* < 0.01 and correlation > 0.8) (Fig. 1A and Fig. S1A). Among them, *ANGPTL4* was one of the most significantly up-regulated DEGs (Fig. 1A), suggesting a potential role of ANGPTL4 during *H. pylori* infection. Next, we analyzed the levels of ANGPTL4 protein in gastric mucosa of 50 uninfected individuals and 131 *H. pylori*-infected patients. First, compared to uninfected individuals, ANGPTL4 protein levels of *H. pylori*-infected patients were significantly higher within the gastric mucosa (Fig. 1B). Second, within the gastric mucosa, ANGPTL4 protein levels of *H. pylori*-infected patients were positively correlated with the levels of *H. pylori* colonization (Fig. 1C), together indicating an induction of ANGPTL4 by *H. pylori* infection. Third, within the gastric mucosa, higher ANGPTL4 protein levels of *H. pylori*-infected patients were strongly associated with more severe gastritis (Fig. 1D), indicating that, during *H. pylori* infection, ANGPTL4 might exert roles of promoting inflammation. Furthermore, similar findings were made when we analyzed the gene expression of *ANGPTL4* by real-time polymerase chain reaction (PCR) in these samples (Fig. S1B to D).

The *cagA*, as a key virulence factor of *H. pylori*, has been reported to be closely associated with gastritis development [21]. In humans, compared to *cagA*-negative patients, both *ANGPTL4* gene (Fig. S1E) and ANGPTL4 protein (Fig. 1E) expressions within the gastric mucosa of *cagA*-positive patients were significantly higher. In mice, *Angptl4* gene expression was also detected in wild-type (WT) *H. pylori*-infected mice, but not in *ΔcagA*-infected mice, reaching a peak 12 weeks post-infection (p.i.) (Fig. 1F), together suggesting an important role of *cagA* in increasing ANGPTL4 in vivo. Then, compared to either uninfected or *ΔcagA*-infected mice and *cagA*-negative patients, the levels of ANGPTL4 protein were higher in gastric mucosa of WT *H. pylori*-infected mice and *cagA*-positive *H. pylori*-infected patients by immunohistochemical staining (Fig. 1G) as well as Western blots (Fig. 1H), respectively. Then, compared to the human primary gastric mucosa either not infected or infected with *ΔcagA*, the expressions of *ANGPTL4* gene and ANGPTL4 protein were significantly increased in/from those infected with WT *H. pylori* in infection dose-dependent as well as time-dependent manners ex vivo (Fig. 1I). Similar findings were made when we analyzed the expressions of *Angptl4* gene and ANGPTL4 protein in/from mouse primary gastric mucosa infected with WT *H. pylori* or *ΔcagA* ex vivo (Fig. 1J). Notably, it has been reported that flANGPTL4 can undergo proteolytic processing to release nANGPTL4 and cANGPTL4 [16]; here, we always detected flANGPTL4 (65 kDa) and cANGPTL4 (37 kDa) from gastric mucosa in our systems (Fig. 1H to J). Overall, our findings above indicate that, within the *H. pylori*-infected gastric mucosa of humans and mice, ANGPTL4 is actually increased.

### *H. pylori* infects GECs to up-regulate ANGPTL4

**Fig. 2.**
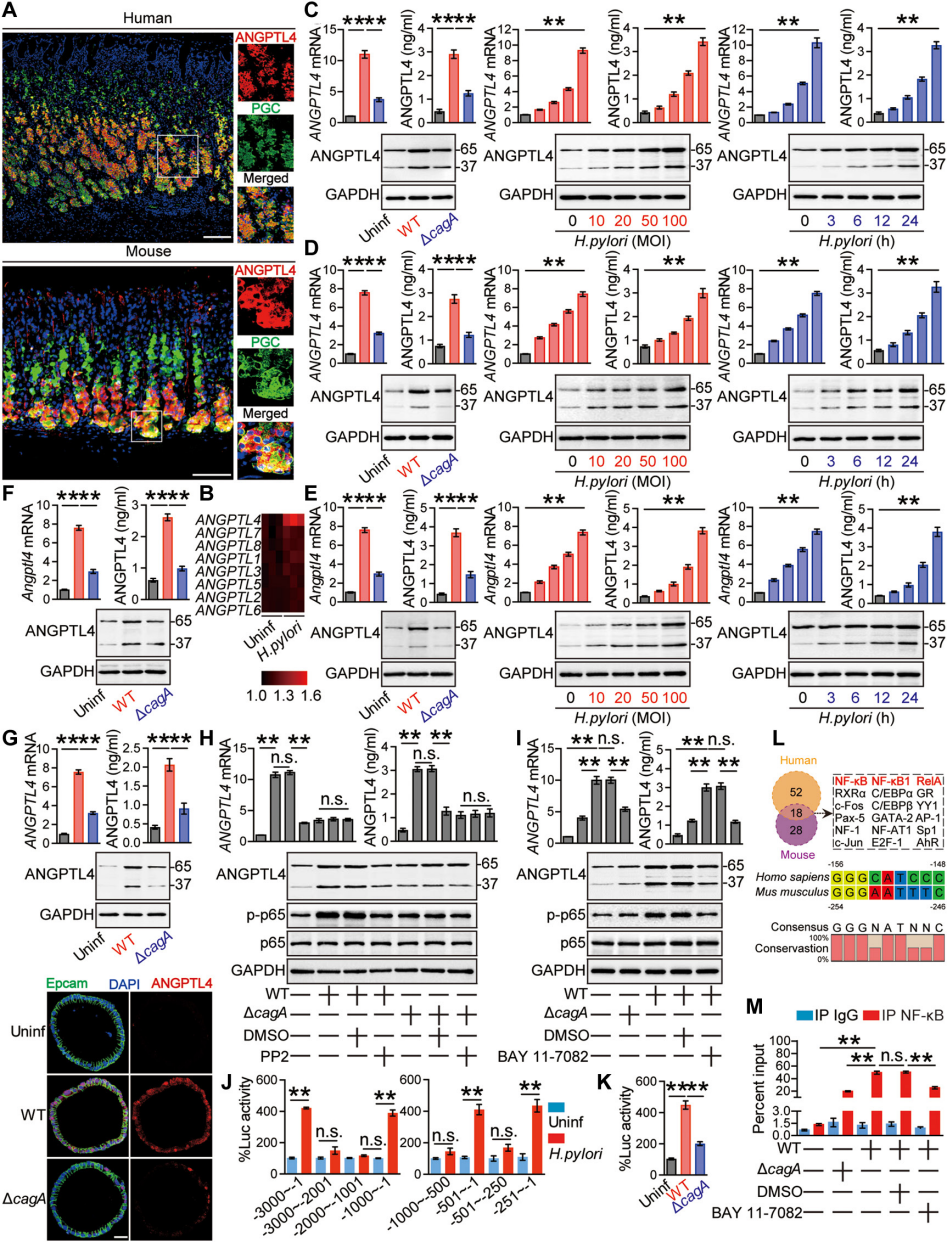
*H. pylori* stimulates GECs to produce ANGPTL4. (A) Representative immunofluorescence staining images showing ANGPTL4-expressing (red) pepsinogen II/PGC^+^ GECs (green) in gastric mucosa of *H. pylori*-infected patients or *H. pylori*-infected mice. Scale bars, 100 μm. (B) ANGPTL family mRNA expressions in WT *H. pylori*-infected and uninfected AGS cells (MOI = 100, 6 h) were compared by microarray (*n* = 3). (C to E) *ANGPTL4/Angptl4* gene and ANGPTL4 protein expressions in WT *H. pylori*-infected, *ΔcagA*-infected, and uninfected AGS cells (C), human primary GECs (D), or mouse primary GECs (E) (MOI = 100, 24 h), and *ANGPTL4/Angptl4* gene and ANGPTL4 protein expressions in WT *H. pylori*-infected and uninfected AGS cells (C), human primary GECs (D), or mouse primary GECs (E) at different time points (MOI = 100) or infected with different MOIs (24 h) were analyzed by real-time PCR, ELISA, and Western blot (*n* = 5). (F and G) *Angptl4/ANGPTL4* gene and ANGPTL4 protein expressions in WT *H. pylori*-infected, *ΔcagA*-infected, and uninfected mouse (F) and human (G) gastric organoids (MOI = 100, 24 h) were analyzed by real-time PCR, ELISA, Western blot, and immunofluorescence (*n* = 5). Scale bars, 50 μm. (H and I) AGS cells were pretreated with or without PP2 (H) and BAY 11-7082 (I) and then infected with WT *H. pylori* or *ΔcagA* (MOI = 100) for 24 h. *ANGPTL4* gene expression and ANGPTL4, p65, and p-p65 proteins were analyzed by real-time PCR, ELISA, and Western blot (*n* = 5). (J and K) AGS cells were transfected with luciferase reporter constructs containing the *ANGPTL4*-luc promoter for 4 h. Luciferase activity was measured to assess *ANGPTL4* promoter activity after WT *H. pylori* or *ΔcagA* infection (MOI = 100) for 24 h (*n* = 5). (L) The transcription factor binding sites were predicted by the PROMO website using a 3,000-bp conserved segment of *ANGPTL4*/*Angptl4* promoter. A conserved sequence of NF-κB binding site with higher JASPAR scores in humans and mice. (M) ChIP assay in AGS cells infected with WT *H. pylori* (pretreated with or without BAY 11-7082) or Δ*cagA*, followed by PCR with primers designed for NF-κB binding site of *ANGPTL4* promoter region (*n* = 5). Data are representative of 2 independent experiments. Data are shown as mean ± SEM and analyzed by Student’s *t* test, Mann–Whitney *U* test and one-way ANOVA. Western blot results are run in parallel and contemporaneously. *P < 0.05, **P < 0.01, n.s. *P* > 0.05 for groups connected by horizontal lines.

Within the gastric mucosa, GECs are regarded to be not only the first-contacted cells but also the major effector cells modulated by *H. pylori* [21]. Interestingly, within the *H. pylori*-infected gastric mucosa of patients and mice, we therefore found that ANGPTL4 was expressed in pepsinogen II/PGC^+^ GECs (Fig. 2A), suggesting that, within the *H. pylori*-infected gastric mucosa, GECs are the ANGPTL4 source cells. Then, we screened all of the ANGPTL family members in *H. pylori*-infected AGS cells and identified *ANGPTL4* as the most increased *ANGPTLs* induced by *H. pylori* infection (Fig. 2B). Then, we showed that *H. pylori*-infected AGS cells increased *ANGPTL4* gene and ANGPTL4 protein expressions in infection dose-dependent as well as time-dependent manners (Fig. 2C). Moreover, compared to uninfected AGS cells or *ΔcagA*-infected AGS cells, WT *H. pylori*-infected AGS cells also significantly increased *ANGPTL4* gene and ANGPTL4 protein expressions (Fig. 2C). Similar findings were made when we detected *ANGPTL4* gene and ANGPTL4 protein expressions in/from other human GEC lines (Fig. S2A to C). Further transwell assays showed that bacterium-cell contacts were necessary for the *ANGPTL4* gene and ANGPTL4 protein expressions in/from *H. pylori*-infected AGS cells (Fig. S2D). Then, we also confirmed that *H. pylori* infection induced human primary GECs (Fig. 2D) as well as mouse primary GECs (Fig. 2E) to increase *ANGPTL4*/*Angptl4* expression and ANGPTL4 production, both of which were dependent of *cagA* and in infection dose- and time-dependent manners. Importantly, using established mouse and human gastric organoids (Fig. S2E and F), we further confirmed that *H. pylori* infection induced mouse (Fig. 2F) as well as human (Fig. 2G) gastric organoids to increase *ANGPTL4*/*Angptl4* gene and ANGPTL4 protein expressions in *cagA*-dependent manners. Overall, these data above indicate that increased ANGPTL4 production from GECs can be induced by *H. pylori* infection.

To next investigate the signaling pathways that regulate ANGPTL4 expression in *H. pylori*-infected GECs, we performed experiments to specifically block potentially relevant signaling pathways, and found that only pretreatment with BAY 11-7082 to block the signaling pathway of nuclear factor κB (NF-κB) could effectively decrease the expression of *ANGPTL4* gene in AGS cells infected with WT *H. pylori* (Fig. S2G). Moreover, ANGPTL4 and p65, a direct NF-κB pathway downstream substrate, were predominantly increased and phosphorylated in WT *H. pylori*-infected AGS cells, which were abolished when these cells were pretreated with BAY 11-7082 (Fig. 2H and I). To investigate whether *cagA* can induce ANGPTL4 via NF-κB signaling pathway, we then infected AGS cells with WT or *ΔcagA H. pylori* or transfected AGS cells with *cagA*-pcDNA3.1. As expected, increased ANGPTL4 and p65 phosphorylation were detected in WT *H. pylori*-infected AGS cells or *cagA*-pcDNA3.1-transfected AGS cells, but not in *ΔcagA*-infected AGS cells or pcDNA3.1-transfected AGS cells (Fig. 2H and I and Fig. S2H). Importantly, these increases induced by WT *H. pylori* infection or *cagA*-pcDNA3.1 transfection were abrogated by pretreatment with BAY 11-7082 or with PP2, a *cagA* EPIYA motif phosphorylation inhibitor [22] (Fig. 2H and I and Fig. S2H).

To further investigate the mechanisms of *ANGPTL4* gene transcription induced by *H. pylori* infection, we generated a series of varying lengths of *ANGPTL4*-luc promoter constructs and then performed luciferase reporter assays. We found that *ANGPTL4* promoter (−251/−1) mediated transcription in response to *H. pylori* infection (Fig. 2J) in a *cagA*-dependent manner (Fig. 2K). Interestingly, both human and mouse *ANGPTL4* promoters contained conserved NF-κB binding sites by using PROMO and JASPAR (Fig. 2L and Tables S6 to S8). Furthermore, we performed chromatin immunoprecipitation (ChIP) assay and found that, compared to no infection, *ΔcagA* infection, or pcDNA3.1 transfection, WT *H. pylori* infection and *cagA*-pcDNA3.1 transfection significantly increased NF-κB binding to the *ANGPTL4* promoter in AGS cells, which were abolished when these cells were pretreated with BAY 11-7082 (Fig. 2M and Fig. S2I). Collectively, our data clearly demonstrate that transcription regulation of NF-κB mediated *H. pylori*-induced ANGPTL4 expression in GECs.

### *H. pylori* infection and IL-17A synergistically up-regulate ANGPTL4

**Fig. 3.**
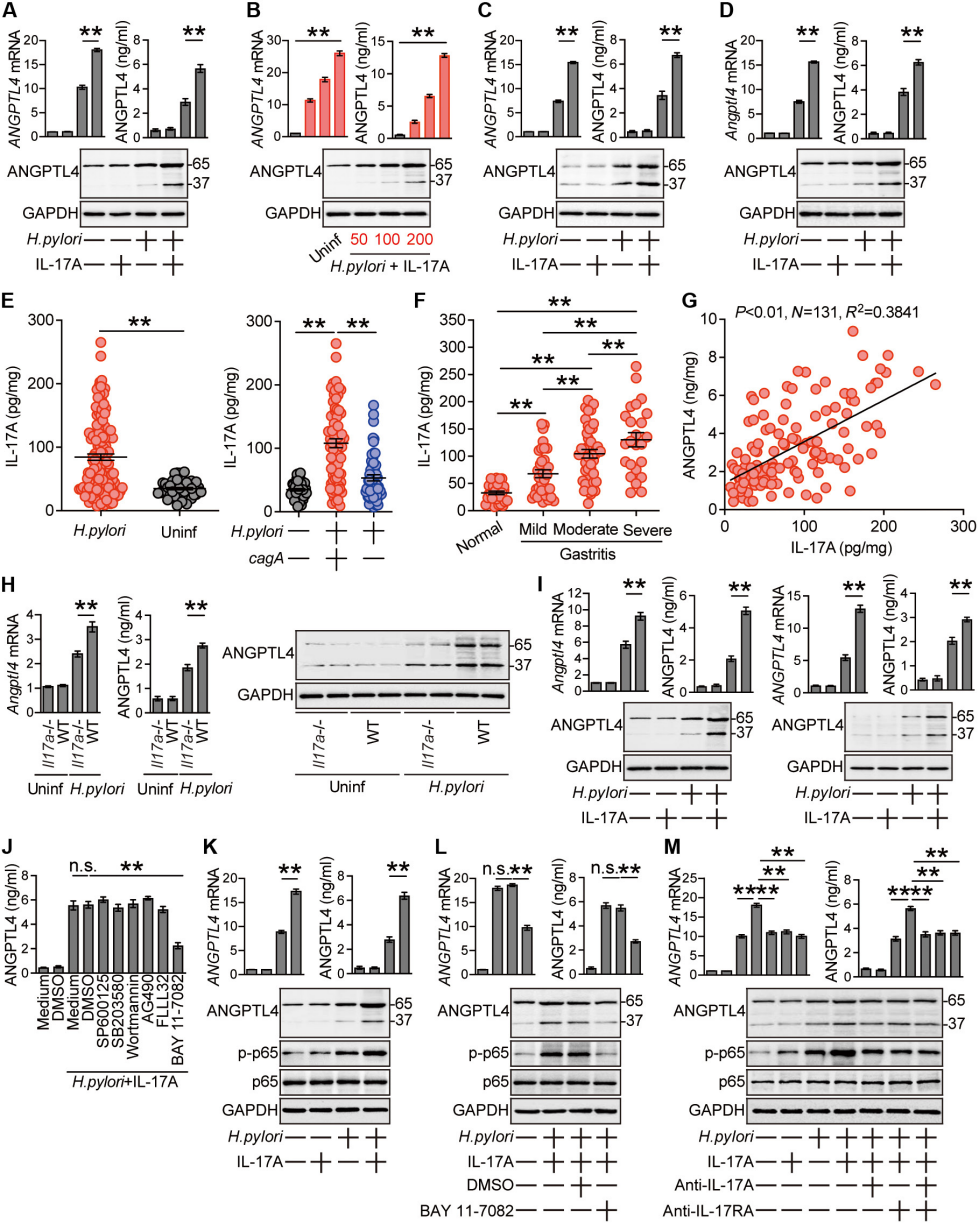
*H. pylori* and IL-17A synergistically induce ANGPTL4. (A to D) *ANGPTL4/Angptl4* gene and ANGPTL4 protein expressions in AGS cells (A), human primary GECs (C), or mouse primary GECs (D) infected with WT *H. pylori* (MOI = 100) in the presence or absence of IL-17A (100 ng/ml) (24 h), or in AGS cells infected with WT *H. pylori* (MOI = 100) in the presence of IL-17A (50, 100, and 200 ng/ml) (24 h) (B) were analyzed by real-time PCR, ELISA, and Western blot (*n* = 5). (E) IL-17A protein expression in gastric mucosa of *H. pylori*-infected (*n* = 131) and uninfected donors (*n* = 50) or in gastric mucosa of *cagA*^+^ *H. pylori*-infected (*n* = 74), *cagA*^−^ *H. pylori*-infected (*n* = 57), and uninfected donors (*n* = 50) was compared. (F) IL-17A protein expression in gastric mucosa of *H. pylori*-infected patients with mild (*n* = 34), moderate (*n* = 45), and severe inflammation (*n* = 24) and with normal gastric histopathology (*n* = 28) was compared. (G) The correlation between ANGPTL4 protein and IL-17A protein in gastric mucosa of *H. pylori*-infected patients was analyzed. (H) *Angptl4* gene and ANGPTL4 protein expressions in gastric mucosa of WT *H. pylori*-infected WT and *Il17a*^−/−^ mice at 12 weeks p.i. were compared (*n* = 5). (I) *ANGPTL4/Angptl4* gene and ANGPTL4 protein expressions in mouse and human gastric organoids infected with WT *H. pylori* (MOI = 100) in the presence or absence of IL-17A (100 ng/ml) (24 h) were analyzed by real-time PCR, ELISA, and Western blot (*n* = 5). (J) AGS cells were pretreated with signal pathway inhibitors and then infected with WT *H. pylori* (MOI = 100) in the presence of IL-17A (100 ng/ml) (24 h). ANGPTL4 protein in AGS cells was compared (*n* = 5). (K and L) AGS cells were pretreated with or without BAY 11-7082 and then infected with WT *H. pylori* (MOI = 100) in the presence or absence of IL-17A (100 ng/ml) (24 h). *ANGPTL4* gene expression and ANGPTL4, p65, and p-p65 proteins were analyzed by real-time PCR, ELISA, and Western blot (*n* = 5). (M) AGS cells were pretreated with anti-IL-17A and/or aiti-IL-17RA Abs and then infected with WT *H. pylori* (MOI = 100) in the presence of IL-17A (100 ng/ml) (24 h). *ANGPTL4* gene expression and ANGPTL4, p65, and p-p65 proteins were analyzed by real-time PCR, ELISA, and Western blot (*n* = 5). Data are representative of 2 independent experiments. Data are shown as mean ± SEM and analyzed by Student’s *t* test, Mann–Whitney *U* test, and one-way ANOVA. Western blot results are run in parallel and contemporaneously. *P < 0.05, **P < 0.01, n.s. *P* > 0.05 for groups connected by horizontal lines.

It is previously reported that T cell responses and T cell-associated inflammatory cytokines play important pathological roles in *H. pylori*-associated gastritis [23,24]. To explore whether inflammatory cytokines from these T cells and inflammatory cytokines that regulate these T cells have potentially synergistic effects on increasing ANGPTL4 from GECs infected with *H. pylori*, we first infected AGS cells with WT *H. pylori* in the presence or absence of inflammatory cytokines. Interestingly, only interleukin-17A (IL-17A) exerted synergistic effects on *ANGPTL4* gene expression (Fig. 3A and Fig. S3A) as well as ANGPTL4 protein expression (Fig. 3A) in dose-dependent manners (Fig. 3B). However, there was no synergistic effects on *IL17A* expression in WT *H. pylori*-infected AGS cells by ANGPTL4 (Fig. S3B). Similar findings were also made when we detected *ANGPTL4*/*Angptl4* gene and ANGPTL4 protein expressions in human primary GECs (Fig. 3C) or mouse primary GECs (Fig. 3D) infected with WT *H. pylori* in the presence or absence of IL-17A. We further detected IL-17A protein levels in human gastric mucosa from 131 *H. pylori*-infected patients and 50 uninfected individuals. First, compared to uninfected individuals, the levels of IL-17A protein were higher in gastric mucosa from *H. pylori*-infected patients (Fig. 3E). Second, compared to *cagA*-negative individuals, the levels of IL-17A protein in *cagA*-positive patients were significantly higher (Fig. 3E). Third, within gastric mucosa from *H. pylori*-infected patients, higher IL-17A protein levels were closely associated with more severe gastritis (Fig. 3F). Importantly, within gastric mucosa from *H. pylori*-infected patients, there were significant positive correlations between ANGPTL4 and IL-17A (Fig. 3G). Similar findings were made when we analyzed *IL17A* gene expression by real-time PCR in these samples (Fig. S3C to E). Most importantly, these findings were confirmed in gastric mucosa of *H. pylori*-infected WT and *Il17a*^−/−^ mice in vivo as we found decreased ANGPTL4 in *Il17a*^−/−^ mice (Fig. 3H). Using established mouse and human gastric organoids, we further confirmed that *H. pylori* infection and IL-17A synergistically induced mouse (Fig. 3I, left panel) and human (Fig. 3I, right panel) gastric organoids to increase *ANGPTL4*/*Angptl4* expression and ANGPTL4 production. As T helper 17 (T_H_17) cells mainly produce IL-17, which has been reported to be involved in *H. pylori* infection [24], we then analyzed T_H_17 cell responses in gastric mucosa during *H. pylori* infection and found that *H. pylori* infection indeed induced gastric T_H_17 cell responses in vivo (Fig. S3F). The data together suggest that *H. pylori* and IL-17A induce ANGPTL4 synergistically.

Signaling pathway blocking experiments showed that only pretreatment with BAY 11-7082 to block the signaling pathway of NF-κB effectively decreased *ANGPTL4* expression and ANGPTL4 induction by WT *H. pylori* and IL-17A (Fig. 3J and Fig. S3G). Furthermore, ANGPTL4 and p65, a direct NF-κB pathway downstream substrate, were predominantly increased and phosphorylated in AGS cells induced by WT *H. pylori* and IL-17A (Fig. 3K), which were abolished when these cells were pretreated with BAY 11-7082 (Fig. 3L) or when the IL-17A–IL-17A receptor interactions were blocked by using either anti-IL-17A or anti-IL-17 receptor A (IL-17RA) antibody (Ab) (Fig. 3M). Overall, our data indicate that, in *H. pylori*-infected GECs, the increased ANGPTL4 can be synergistically augmented by inflammatory cytokine IL-17A via NF-κB signaling pathway activation.

### ANGPTL4 increases gastric bacterial colonization, inflammation and Treg accumulation, but decreases neutrophil accumulation and CLDN1 expression within the gastric mucosa during *H. pylori* infection

**Fig. 4.**
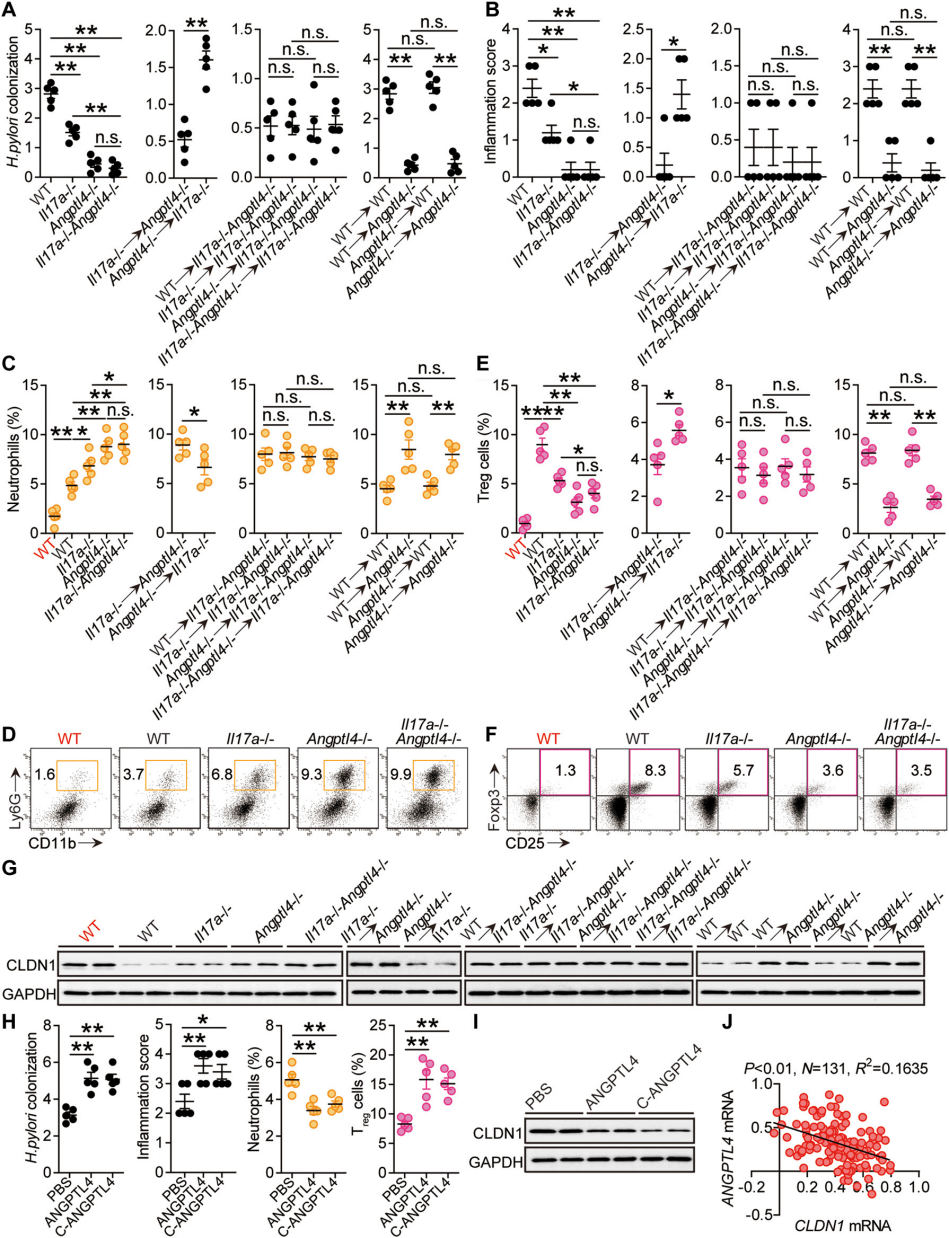
ANGPTL4 increases bacterial burden, inflammation, and T_reg_ accumulation but decreases neutrophil accumulation and CLDN1 expression in gastric mucosa during *H. pylori* infection. (A and B) The bacteria colonization (A) and histological scores of inflammation (B) in gastric mucosa of WT *H. pylori*-infected WT, *Il17a*^−/−^, *Angptl4*^−/−^, and *Il17a*^−/−^*Angptl4*^−/−^ mice or in gastric mucosa of WT *H. pylori*-infected BM chimera mice at 12 weeks p.i. were compared (*n* = 5). (C and E) The levels of neutrophils (C) and T_regs_ (E) in gastric mucosa of uninfected WT mice (red) and WT *H. pylori*-infected WT, *Il17a*^−/−^, *Angptl4*^−/−^, and *Il17a*^−/−^*Angptl4*^−/−^ mice or in gastric mucosa of WT *H. pylori*-infected BM chimera mice at 12 weeks p.i. were compared (*n* = 5). Results are expressed as the percentage of neutrophils in CD45^+^ cells and the percentage of T_regs_ among CD4^+^ T cells. (D and F) Representative dot plots of neutrophils (D) or T_regs_ (F) (defined as described in Fig. S4A) in gastric mucosa of uninfected WT mice (red) and WT *H. pylori*-infected WT, *Il17a*^−/−^, *Angptl4*^−/−^, and *Il17a*^−/−^*Angptl4*^−/−^ mice at 12 weeks p.i. (G) CLDN1 proteins in gastric mucosa of uninfected WT mice (red) and WT *H. pylori*-infected WT, *Il17a*^−/−^, *Angptl4*^−/−^, and *Il17a*^−/−^*Angptl4*^−/−^ mice or in gastric mucosa of WT *H. pylori*-infected BM chimera mice at 12 weeks p.i. were analyzed. (H) The bacteria colonization, histological scores of inflammation, and the levels of neutrophils and T_regs_ in gastric mucosa of WT mice injected with ANGPTL4, cANGPTL4, or PBS control at 12 weeks p.i. were compared (*n* = 5). (I) CLDN1 proteins in gastric mucosa of WT mice injected with ANGPTL4, cANGPTL4, or PBS control at 12 weeks p.i. were analyzed. (J) The correlation between *ANGPTL4* expression and *CLDN1* expression in gastric mucosa of *H. pylori*-infected patients was analyzed. Results are expressed as log_10_(fold change). Data are representative of 2 independent experiments. Data are shown as mean ± SEM and analyzed by Student’s *t* test, Mann–Whitney *U* test, and one-way ANOVA. Western blot results are run in parallel and contemporaneously. *P < 0.05, **P < 0.01, n.s. *P* > 0.05 for groups connected by horizontal lines.

To next investigate the possible biological effects of ANGPTL4 during *H. pylori* infection in vivo, we first analyzed the bacterial colonization levels within the gastric mucosa at 12 weeks p.i. among WT, *Il17a*^−/−^, *Angptl4*^−/−^, and *Il17a*^−/−^*Angptl4*^−/−^ mice, and found that lacking IL-17A and/or ANGPTL4 in *Il17a*^−/−^, *Angptl4*^−/−^, and *Il17a*^−/−^*Angptl4*^−/−^ mice significantly reduced gastric *H. pylori* colonization when compared to that in WT mice, which was more pronounced when ANGPTL4 was knocked out in *Angptl4*^−/−^ and *Il17a*^−/−^*Angptl4*^−/−^ mice compared to that in *Il17a*^−/−^ mice (Fig. 4A, left panel). Our findings above suggest that, within the *H. pylori*-infected gastric mucosa, ANGPTL4 is the actual effector factor, and IL-17A only exerts augmenting roles in vivo.

Within the *H. pylori*-infected gastric mucosa, it is found that T_H_ cells are likely the source cells that produce IL-17A [24], and we previously showed that GECs are likely the source cells that produce ANGPTL4 (Fig. 2). Therefore, bone marrow (BM) chimera mice were generated to determine the contributions of BM-derived IL-17A-producing cells (including T_H_ cells) as well as non-BM-derived ANGPTL4-producing cells (including GECs) to gastric bacterial colonization. First, *Il17a*^−/−^ BM into *Angptl4*^−/−^ mice effectively reduced gastric *H. pylori* colonization when compared to that in *Angptl4*^−/−^ BM into *Il17a*^−/−^ mice (Fig. 4A, middle left panel), indicating that both BM-derived IL-17A-producing cells and non-BM-derived ANGPTL4-producing cells contribute to increasing gastric bacterial colonization. Second, to further exclude the possibilities that non-BM-derived cells may also produce IL-17A and BM-derived cells may also produce ANGPTL4 within the gastric mucosa, other BM chimera mouse groups (*Il17a*^−/−^ BM, *Angptl4*^−/−^ BM, *Il17a*^−/−^*Angptl4*^−/−^ BM into *Il17a*^−/−^*Angptl4*^−/−^ mice) were generated, and we could not detect any role of potential non-BM-derived IL-17A or BM-derived ANGPTL4 on gastric bacterial colonization (Fig. 4A, middle right panel). Finally, compared to *Angptl4*^−/−^ BM into WT mice, WT BM into *Angptl4*^−/−^ mice was not able to correct the increased *H. pylori* colonization within the gastric mucosa, again indicating that this defect is actually associated with non-BM-derived ANGPTL4-producing cells (Fig. 4A, right panel). Taken together, by using these BM chimera mice above, we could convincingly show that non-BM-derived ANGPTL4-producing cells were largely responsible for gastric bacterial colonization in these models (Fig. 4A). Therefore, these findings demonstrate that ANGPTL4 produced from non-BM-derived cells exerts essential roles in increasing gastric bacterial colonization in vivo.

Chronic gastritis induced by *H. pylori* infection is believed to be characterized as the infiltrations of several inflammatory immune cells [25,26]. Therefore, also by using these knockout mice as well as BM chimera mice described above, we evaluated the gastric inflammatory responses during *H. pylori* infection. First, lacking IL-17A and/or ANGPTL4 in *Il17a*^−/−^, *Angptl4*^−/−^, and *Il17a*^−/−^*Angptl4*^−/−^ mice effectively reduced inflammation within the gastric mucosa when compared to that in WT mice (Fig. 4B), which was more pronounced when ANGPTL4 was knocked out in *Angptl4*^−/−^ and *Il17a*^−/−^*Angptl4*^−/−^ mice compared to that in *Il17a*^−/−^ mice (Fig. 4B). Then, within the *H. pylori*-infected gastric mucosa, non-BM-derived ANGPTL4-producing cells were largely responsible for inflammation, which was also confirmed by using BM chimera mice (Fig. 4B). Collectively, our data suggest that, during *H. pylori* infection, ANGPTL4 can increase gastric inflammation in vivo.

To next analyze the infiltrated immune cell types regulated by ANGPTL4 within the gastric mucosa, we obtained the suspensions of stomach cells by enzymatic digestion and then analyzed these samples by multiple-color flow cytometry. After gating on CD45^+^ cells, a sequential gating strategy of excluding cells expressing Siglec-F (eosinophils), CD49b [natural killer (NK) cells], CD19 (B cells), or CD90 (T cells), and further based on the expressions of CD11b and Ly6G, allowed for the characterization of neutrophils (CD45^+^CD90^−^CD19^−^CD49b^−^Siglec-F^−^CD11b^+^Ly6G^+^), and after gating on CD45^+^ cells, another sequential gating strategy based on the differential expressions of CD19, CD49b, CD3, CD4, CD25, and Foxp3 allowed for the characterization of NK cells (CD45^+^CD19^−^CD49b^+^), B cells (CD45^+^CD3^−^CD49b^−^CD19^+^), CD4^−^ T cells (CD45^+^CD19^−^CD49b^−^CD3^+^CD4^−^), CD4^+^ T cells (CD45^+^CD19^−^CD49b^−^CD3^+^CD4^+^), or regulatory CD4^+^ T cells (T_regs_) (CD45^+^CD19^−^CD49b^−^CD3^+^CD4^+^CD25^+^Foxp3^+^) (Fig. S4A). Compared the levels of these cells (neutrophils, NK cells, B cells, CD4^−^ T cells, CD4^+^ T cells, and T_regs_) within the gastric mucosa at 12 weeks p.i., lack of ANGPTL4 in *Angptl4*^−*/*−^ mice only led to increased gastric (Fig. 4C and D and Fig. S8B) neutrophils and reduced gastric T_regs_ (Fig. 4E and F and Fig. S9B); but not changed these cells above in BM (Fig. S5), blood (Fig. S6), or spleen (Fig. S7). Then, within the *H. pylori*-infected gastric mucosa, these results were also confirmed by the BM chimera experiments above in which non-BM-derived ANGPTL4-producing cells were largely responsible for decreased gastric neutrophils (Fig. 4C and Fig. S8A and B) and increased gastric T_regs_ (Fig. 4E and Fig. S9A and B). Similar findings were made when we analyzed the expressions of *Ly6g* gene (Fig. S10A), Ly6G protein (Fig. S11), *Foxp3* gene (Fig. S10C), and Foxp3 protein (Fig. S12) by real-time PCR as well as immunohistochemical staining in these samples. Furthermore, injection of ANGPTL4 significantly decreased gastric neutrophils and increased gastric T_regs_, bacterial colonization, and inflammation (Fig. 4H and Figs. S8C, S9C, and S10E). Interestingly, claudin 1 (CLDN1) changed the opposite direction as that of ANGPTL4, i.e., the lack of ANGPTL4 was accompanied by significantly increased CLDN1 in these samples (Fig. 4G and I and Fig. S10D and E). Furthermore, in gastric mucosa of *H. pylori*-infected patients, there was a significant negative correlation between *CLDN1* expression and *ANGPTL4* expression (Fig. 4J). Collectively, these data above indicate that, within the *H. pylori*-infected gastric mucosa, ANGPTL4 exerts important roles in increasing gastric bacterial burden, inflammation as well as T_reg_ accumulation, and decreasing neutrophil accumulation and CLDN1 expression.

### ANGPTL4 suppresses CLDN1 and CXCL1 in GECs by binding to ITGAV to inhibit ERK, leading to decreased neutrophil accumulation and increased bacterial burden during *H. pylori* infection

**Fig. 5.**
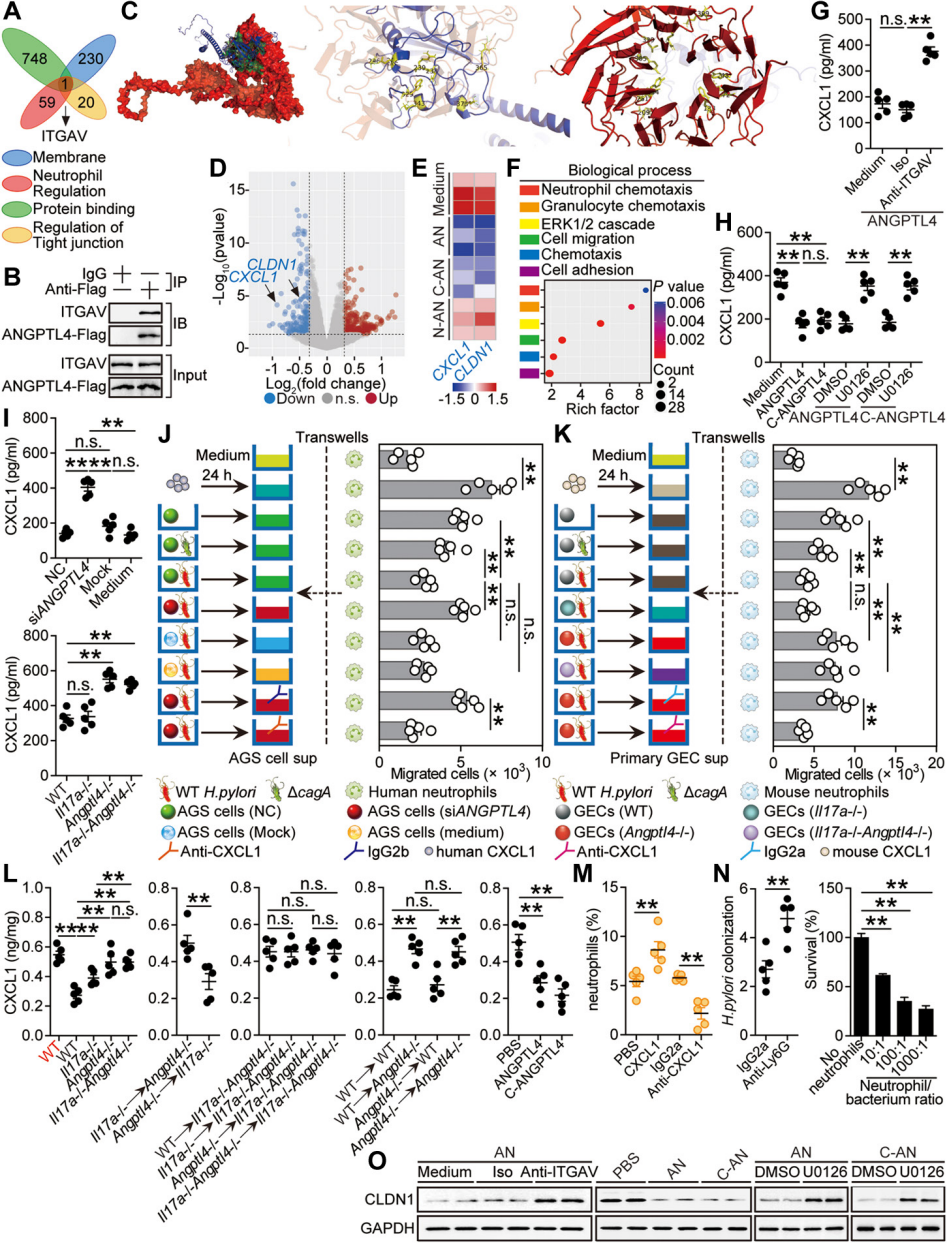
ANGPTL4 suppresses CLDN1 and CXCL1 in GECs by binding to ITGAV to inhibit ERK, leading to decreased neutrophil accumulation and increased bacterial burden during *H. pylori* infection. (A) The members of the 4 GO terms in “Cellular Component,” “Biological Process,” “Molecular Function,” and “KEGG” (described in Fig. S13B) have overlaps by MS analysis of AGS cells expressing the Flag-tagged ANGPTL4 (ANGPTL4-Flag) immunoprecipitated (IP) with anti-Flag Abs. ITGAV is the core member of each GO terms. (B) AGS cells expressing ANGPTL4-Flag were lysed and IP with IgG or anti-Flag Abs. The IP samples were subjected to Western blot analysis with the indicated Abs to detect the interaction with ITGAV. (C) The optimally predicted protein–protein complex obtained from HADDOCK’s easy interface: left, an overarching schematic of ANGPTL4 (represented as a blue cartoon model) binding with ITGAV (indicated as a red surface model); middle, the key active residues involved in the binding of ANGPTL4 to ITGAV, denoted as yellow sticks within the blue structural domain of ANGPTL4; right, the key active residues crucial for ITGAV binding with ANGPTL4, denoted as yellow sticks within the red structural domain of ITGAV. (D) VolcanoPlot reveals gene changes in AGS cells stimulated with ANGPTL4 (1 μg/ml) for 24 h. (E) Heatmap reveals *CXCL1* and *CLDN1* changes in AGS cells stimulated with ANGPTL4, cANGPTL4, or nANGPTL4 (1 μg/ml) for 24 h. (F) Compared to unstimulated AGS cells, significantly changed genes in ANGPTL4-stimulated AGS cells were clustered with GO analysis, and the top 6 GO terms of “Biological Process” are shown. (G) AGS cells were pretreated with anti-ITGAV Abs and then stimulated with ANGPTL4 (1 μg/ml) for 24 h. CXCL1 production was measured in cell culture supernatants by ELISA (*n* = 5). (H) AGS cells were pretreated with U0126 and then stimulated with ANGPTL4 or cANGPTL4 (1 μg/ml) for 24 h. CXCL1 production was measured in cell culture supernatants by ELISA (*n* = 5). (I) *ANGPTL4* siRNA, nonspecific control siRNA (NC), or Lipofectamine 2000 only (mock) pretreated AGS cells and primary GECs from uninfected WT, *Il17a*^−/−^, *Angptl4*^−/−^, and *Il17a*^−/−^*Angptl4*^−/−^ mice were infected with WT *H. pylori* (MOI = 100) for 24 h. CXCL1 production was measured in cell culture supernatants by ELISA (*n* = 5). (J and K) Neutrophil migrations were assessed by transwell assays as described in Methods and statistically analyzed (*n* = 5). (L) CXCL1 proteins in gastric mucosa of uninfected WT mice (red) and WT *H. pylori*-infected WT, *Il17a*^−/−^, *Angptl4*^−/−^, and *Il17a*^−/−^*Angptl4*^−/−^ mice, in gastric mucosa of WT *H. pylori*-infected BM chimera mice, or in gastric mucosa of WT mice injected with ANGPTL4, cANGPTL4, or PBS control at 12 weeks p.i. were compared (*n* = 5). (M) The neutrophil percentages in gastric mucosa of WT *H. pylori*-infected mice injected with CXCL1 or PBS control, or anti-CXCL1 Abs or control IgG at 12 weeks p.i. were compared (*n* = 5). Results are expressed as the percentage of neutrophils in CD45^+^ cells. (N) Left: Bacteria colonization in gastric mucosa of WT *H. pylori*-infected mice injected with anti-Ly6G Abs or control IgG at 12 weeks p.i. was compared (*n* = 5). Right: Killing of *H. pylori* in vitro by human neutrophils at the indicated neutrophil/bacterium ratios. Bacterial survival was measured as described in Methods and statistically analyzed (*n* = 3). (O) CLDN1 proteins in AGS cells stimulated with ANGPTL4 or cANGPTL4 (1 μg/ml) (pretreated with or without U0126 or anti-ITGAV Abs) for 24 h were analyzed. Data are representative of 2 independent experiments. Data are shown as mean ± SEM and analyzed by Student’s *t* test, Mann–Whitney *U* test, and one-way ANOVA. Western blot results are run in parallel and contemporaneously. *P < 0.05, **P < 0.01, n.s. *P* > 0.05 for groups connected by horizontal lines. sup, supernatant.

We next tried to investigate the underlying mechanisms by which ANGPTL4 suppressed neutrophil infiltration and CLDN1 expression within the *H. pylori*-infected gastric mucosa. First, by using generated AGS cells stably expressing ANGPTL4-Flag, immunoprecipitation (IP) followed by mass spectrometry (MS) assay was performed to identify potential partners interacting with ANGPTL4 (Fig. S13A). Further protein network analysis of the MS results identified top 6 gene ontology (GO) terms of “KEGG,” “Biological Process,” “Molecular Function,” as well as “Cellular Component” significantly enriched in the candidate interacting partners (Fig. S13B). We then identified one candidate, integrin αV (ITGAV), that overlapped and presented of the GO term “membrane” in “Cellular Component,” the GO term “neutrophil regulation” in “Biological Process,” the GO term “protein binding” in “Molecular Function,” and the GO term “regulation of tight junction” in “KEGG” (Fig. 5A). Moreover, the interactions between ANGPTL4 and ITGAV were validated by co-IP experiments by using generated AGS cells stably expressing ANGPTL4-Flag (Fig. 5B). To further confirm whether ANGPTL4 colocalizes with ITGAV on GEC membrane, AGS cells were incubated with ANGPTL4 at 4 °C. Then, confocal microscopy using Abs for ANGPTL4 and ITGAV demonstrated that ANGPTL4 colocalized with ITGAV on the plasma membranes of AGS cells (Fig. S13C). In silico prediction of the interaction of ANGPTL4 with ITGAV showed that there were several residues between ANGPTL4 (ASN238, ARG239, GLU242, GLU296, TRP329, LYS343, ARG365, and LYS373) (Fig. 5C, middle panel) and ITGAV (TYR48, LYS72, TRP123, TRP209, TYR251, TYR254, TYR305, LEU329, ARG369, and LYS399) (Fig. 5C, right panel) in an overarching schematic of ANGPTL4 binding with ITGAV (Fig. 5C, left panel).

ANGPTL4 can undergo proteolytic processing to release nANGPTL4 and cANGPTL4 [16]. We found that, compared to nANGPTL4-treated AGS cells, ANGPTL4-treated AGS cells and cANGPTL4-treated AGS cells had the similar whole-transcriptome patterns (Fig. S13D) and exerted decreased expressions of *CXCL1* and *CLDN1* (Fig. 5D and E and Fig. S14B and I). Then, further “Biological Process” (Fig. 5F) and “KEGG” (Fig. S13F) analyses in ANGPTL4-treated AGS cells revealed that extracellular signal-regulated kinase (ERK) was probably involved in ANGPTL4-associated signaling. More importantly, Ab blocking experiments (Fig. 5G and Fig. S14A) and signaling pathway blocking experiments (Fig. 5H and Fig. S14C) showed that ANGPTL4′ binding to ITGAV contributed to the suppression of CXCL1 production via ERK. Collectively, these data suggest that, on GECs, ANGPTL4 interacts with ITGAV to suppress GEC’s CXCL1 production.

Moreover, CXCL1 productions from AGS cells as well as from mouse primary GECs were regulated in ANGPTL4-dependent manners (Fig. 5I and Fig. S14D). To investigate the contributions of ANGPTL4-CXCL1 axis to the neutrophil migration in vitro, we performed human neutrophil chemotaxis assays and found that the culture supernatants from *ΔcagA*-infected AGS cells pretreated with nonspecific control small interfering RNA (siRNA) (NC) or from WT *H. pylori*-infected AGS cells pretreated with *ANGPTL4* siRNA induced significantly more migration of neutrophils than those from WT *H. pylori*-infected AGS cells pretreated with NC, and these effects were lost upon pretreatment with neutralizing Abs against CXCL1 (Fig. 5J). Then, we performed mouse neutrophil chemotaxis assays and found that the culture supernatants from *ΔcagA*-infected primary GECs of WT mice or from WT *H. pylori*-infected primary GECs of *Angptl4*^−/−^ mice also induced significantly more migration of neutrophils than those from WT *H. pylori*-infected primary GECs of WT mice, and these effects were also lost upon pretreatment with neutralizing Abs against CXCL1 (Fig. 5K). Moreover, lacking IL-17A and/or ANGPTL4 in *Il17a*^−/−^, *Angptl4*^−/−^, and *Il17a*^−/−^*Angptl4*^−/−^ mice resulted in increased CXCL1 production when compared to that in WT mice, which was more pronounced in the absence of ANGPTL4 in *Angptl4*^−/−^ and *Il17a*^−/−^*Angptl4*^−/−^ mice compared to that in *Il17a*^−/−^ mice (Fig. 5L and Fig. S14E); then, non-BM-derived ANGPTL4 was largely responsible for the CXCL1 inhibition, which was also confirmed by using BM chimera mice (Fig. 5L and Fig. S14E); and injection of ANGPTL4 significantly decreased gastric CXCL1 production during *H. pylori* infection (Fig. 5L and Fig. S14E). Then, in gastric mucosa of *H. pylori*-infected patients, there was a significant negative correlation between ANGPTL4 and CXCL1 (Fig. S14F). Finally, we conducted a series of blocking or reconstitution experiments in vivo involving CXCL1 and analyzed the levels of neutrophils within the gastric mucosa at 12 weeks p.i. We found that CXCL1 administration effectively increased gastric accumulation of neutrophils; conversely, CXCL1 neutralization effectively decreased gastric accumulation of neutrophils (Fig. 5M and Fig. S14G). Overall, these data indicate that, within the *H. pylori*-infected gastric mucosa, ANGPTL4 suppresses the productions of CXCL1, contributing to decreased neutrophil accumulation.

To further demonstrate the direct bactericidal capacities of neutrophils in vivo and in vitro, we first performed Ab-mediated neutrophil depletion and found that neutropenic mice cleared significantly less *H. pylori* than the control mice (Fig. 5N, left panel). Then, we performed bacteria-killing assays and found that, as neutrophils increased, bacterial survival significantly decreased (Fig. 5N, right panel). Collectively, these results suggest that neutrophils are critical host defense against *H. pylori*.

Next, Ab blocking experiments and signaling pathway blocking experiments showed that ANGPTL4′ binding to ITGAV contributed to the inhibition of CLDN1 expression via ERK (Fig. 5O and Fig. S14H to J). Furthermore, in *H. pylori*-infected patients, there was a significant negative correlation between ANGPTL4 and CLDN1 in gastric mucosa (Fig. S14K). As the gastric tight junction is formed by ZO-1, occludin, and claudins together [27,28], we further analyzed RNA sequencing data from ANGPTL4-stimulated AGS cells and found that CLDN1 was the most down-regulated one among tight junction molecules, which was also confirmed by real-time PCR (Fig. S14L). Overall, our findings indicate that ANGPTL4 interacts with ITGAV to inhibit CLDN1 expression on GECs.

### ANGPTL4 promotes CCL5 in monocytes by binding to ITGAV to activate NF-κB, leading to increased Treg accumulation during *H. pylori* infection

**Fig. 6.**
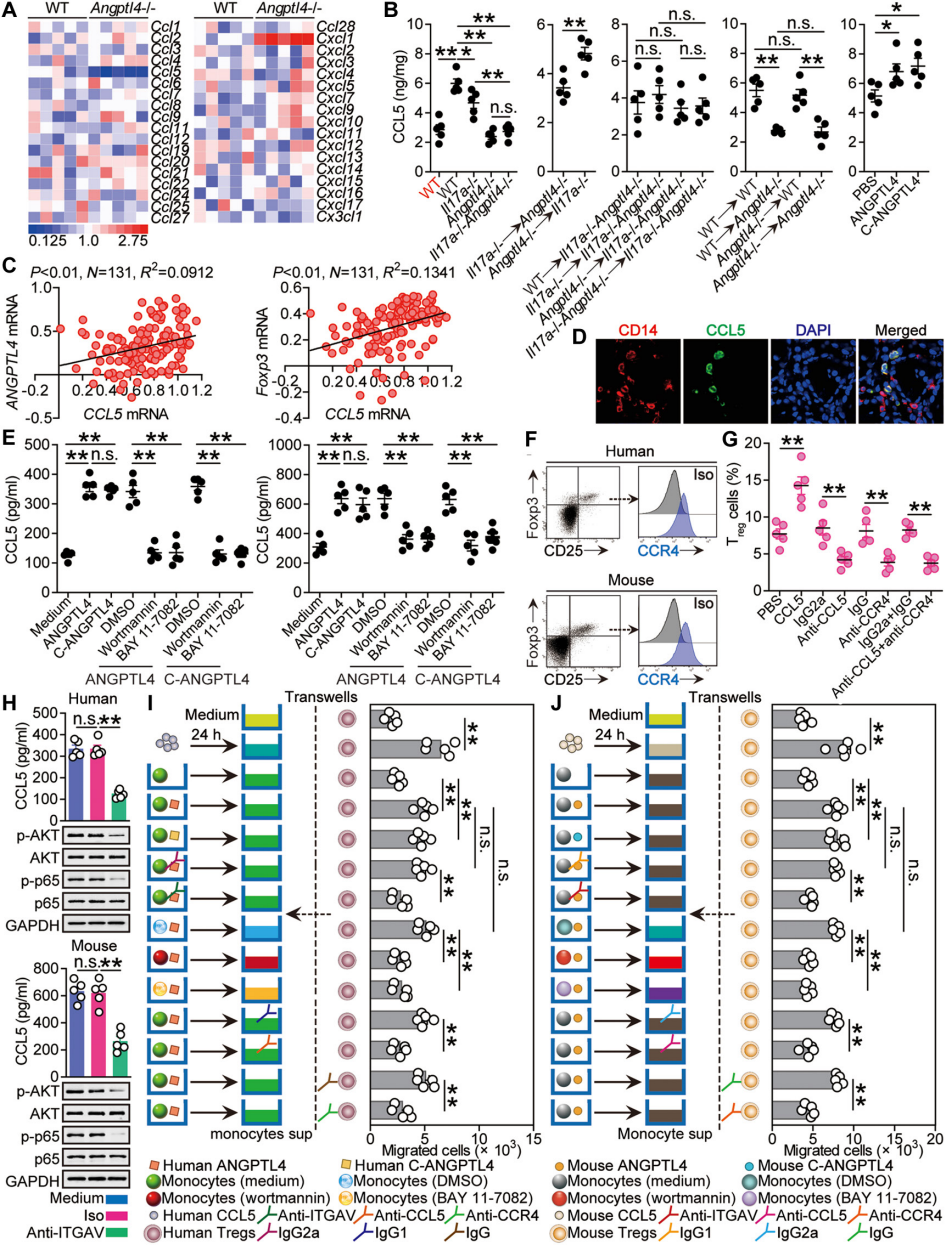
ANGPTL4 promotes CCL5 production in monocytes by binding to ITGAV to activate NF-κB, leading to increased T_reg_ accumulation during *H. pylori* infection. (A) The expressions of chemokine family members in gastric mucosa of WT *H. pylori*-infected WT and *Angptl4*^−/−^ mice at 12 weeks p.i. were analyzed by real-time PCR (*n* = 5). (B) CCL5 proteins in gastric mucosa of uninfected WT mice (red) and WT *H. pylori*-infected WT, *Il17a*^−/−^, *Angptl4*^−/−^ and *Il17a*^−/−^*Angptl4*^−/−^ mice, in gastric mucosa of WT *H. pylori*-infected BM chimera mice, or in gastric mucosa of WT mice injected with ANGPTL4, cANGPTL4, or PBS control at 12 weeks p.i. were compared (*n* = 5). (C) The correlations between *ANGPTL4* expression and *CCL5* expression, and between *CCL5* expression and *Foxp3* expression in gastric mucosa of *H. pylori*-infected patients were analyzed. Results are expressed as log_10_(fold change). (D) Representative immunofluorescence staining images showing CCL5-expressing (green) CD14^+^ monocytes (red) in gastric mucosa from *H. pylori*-infected patients. (E) Human (left panel) or mouse (right panel) monocytes were pretreated with wortmannin or BAY 11-7082 and then stimulated with ANGPTL4 or cANGPTL4 (1 μg/ml) for 24 h. CCL5 production was measured in cell culture supernatants by ELISA (*n* = 5). (F) Representative dot plots of T_regs_ and CCR4 expression on T_regs_ in blood from *H. pylori*-infected patients or WT *H. pylori*-infected mice at 12 weeks p.i. (G) The T_reg_ levels in gastric mucosa of WT *H. pylori*-infected mice injected with CCL5 or PBS control, and/or anti-CCR4 Abs or control IgG at 12 weeks p.i. were compared (*n* = 5). Results are expressed as the percentage of T_regs_ among CD4^+^ T cells. (H) Monocytes were pretreated with anti-ITGAV Abs and then stimulated with ANGPTL4 (1 μg/ml) for 6 or 24 h. CCL5 production, AKT and p-AKT, and p65 and p-p65 proteins were analyzed by ELISA (at 24 h) and Western blot (at 6 h) (*n* = 5). (I and J) T_reg_ migrations were assessed by transwell assays as described in Methods and statistically analyzed (*n* = 5). Data are representative of 2 independent experiments. Data are shown as mean ± SEM and analyzed by Student’s *t* test, Mann–Whitney *U* test, and one-way ANOVA. Western blot results are run in parallel and contemporaneously. *P < 0.05, **P < 0.01, n.s. *P* > 0.05 for groups connected by horizontal lines.

We next tried to investigate the underlying mechanisms by which ANGPTL4 promoted the infiltration of T_regs_ within the *H. pylori*-infected gastric mucosa. First, chemokines within the gastric mucosa at 12 weeks p.i. between WT and *Angptl4*^−/−^ mice were screened, and only *Ccl5* expression was decreased in *Angptl4*^−/−^ mice (Fig. 6A). Moreover, lacking IL-17A and/or ANGPTL4 in *Il17a*^−/−^, *Angptl4*^−/−^, and *Il17a*^−/−^*Angptl4*^−/−^ mice led to reduced CCL5 production when compared to that in WT mice, which was more pronounced when ANGPTL4 was knocked out in *Angptl4*^−/−^ and *Il17a*^−/−^*Angptl4*^−/−^ mice compared to that in *Il17a*^−/−^ mice (Fig. 6B and Fig. S15A); then, non-BM-derived ANGPTL4 was largely responsible for the CCL5 production, which was also confirmed by using BM chimera mice (Fig. 6B and Fig. S15A); and injection of ANGPTL4 significantly increased gastric CCL5 production (Fig. 6B and Fig. S15A).

We next tried to investigate whether gastric accumulation of T_regs_ was regulated by ANGPTL4-CCL5 axis during *H. pylori* infection. First, within the gastric mucosa of patients infected with *H. pylori*, positive correlations between ANGPTL4 and CCL5 as well as between CCL5 and Foxp3 were found (Fig. 6C and Fig. S15B), and CCL5 was also increased and was found to be positively correlated with the severity of gastritis (Fig. S15C to F). Then, the CCL5 expression by CD14^+^ monocytes within the gastric mucosa of patients infected with *H. pylori* (Fig. 6D), along with high ITGAV expression by monocytes in Human Protein Atlas (HPA) immune cell dataset (Fig. S16A), implied that ANGPTL4 probably induced monocytes to produce CCL5 by binding to ITGAV to activate phosphoinositide 3-kinase (PI3K)–AKT–NF-κB signaling pathway [10,29]. Moreover, signaling pathway blocking experiments revealed that ANGPTL4 and cANGPTL4 significantly induced CCL5 via PI3K–AKT–NF-κB (Fig. 6E and Fig. S16B). *H. pylori*-infected mice as well as *H. pylori*-infected patients exhibited high expressions of CCR4, the receptor of chemokine CCL5, on T_regs_ (Fig. 6F). Furthermore, we conducted a series of blocking or reconstitution experiments in vivo involving CCL5–CCR4 axis and analyzed the levels of T_regs_ within the gastric mucosa at 12 weeks p.i., and found that CCL5 administration significantly increased gastric accumulation of T_regs_; conversely, neutralizations of CCL5 and/or CCR4 significantly decreased gastric accumulation of T_regs_ (Fig. 6G and Fig. S16C). Finally, Ab blocking experiments showed that ANGPTL4′ binding to ITGAV on monocytes contributed to the induction of CCL5 production via PI3K–AKT–NF-κB (Fig. 6H and Fig. S16D and E). Collectively, our findings suggest that, within the *H. pylori*-infected gastric mucosa, ANGPTL4 interacts with ITGAV on monocytes to induce CCL5 production by activating PI3K–AKT–NF-κB pathway, which promotes T_reg_ accumulation via CCL5–CCR4 axis.

To next evaluate the contributions of ANGPTL4–ITGAV–CCL5–CCR4 axis to T_reg_ migration, we performed in vitro human T_reg_ chemotaxis assays and found that culture supernatants from ANGPTL4/cANGPTL4-stimulated human monocytes induced significantly more human T_reg_ migration than culture supernatants from unstimulated monocytes, and these effects were lost upon when pretreated with PI3K-AKT inhibitor wortmannin or NF-κB inhibitor BAY 11-7082 as well as with neutralizing Abs against ITGAV, CCL5, or CCR4 (Fig. 6I). Similar findings were made when we analyzed mouse T_reg_ migration by using culture supernatants from mouse monocytes with these treatments (Fig. 6J). Taken together, the results above suggest that, within the gastric mucosa during *H. pylori* infection, ANGPTL4–ITGAV–CCL5–CCR4 axis contributes to T_reg_ accumulation.

### *ANGPTL4 promotes Treg proliferation by binding to ITGAV to activate NF-κB, aggravating inflammation during* H. pylori *infection*

**Fig. 7.**
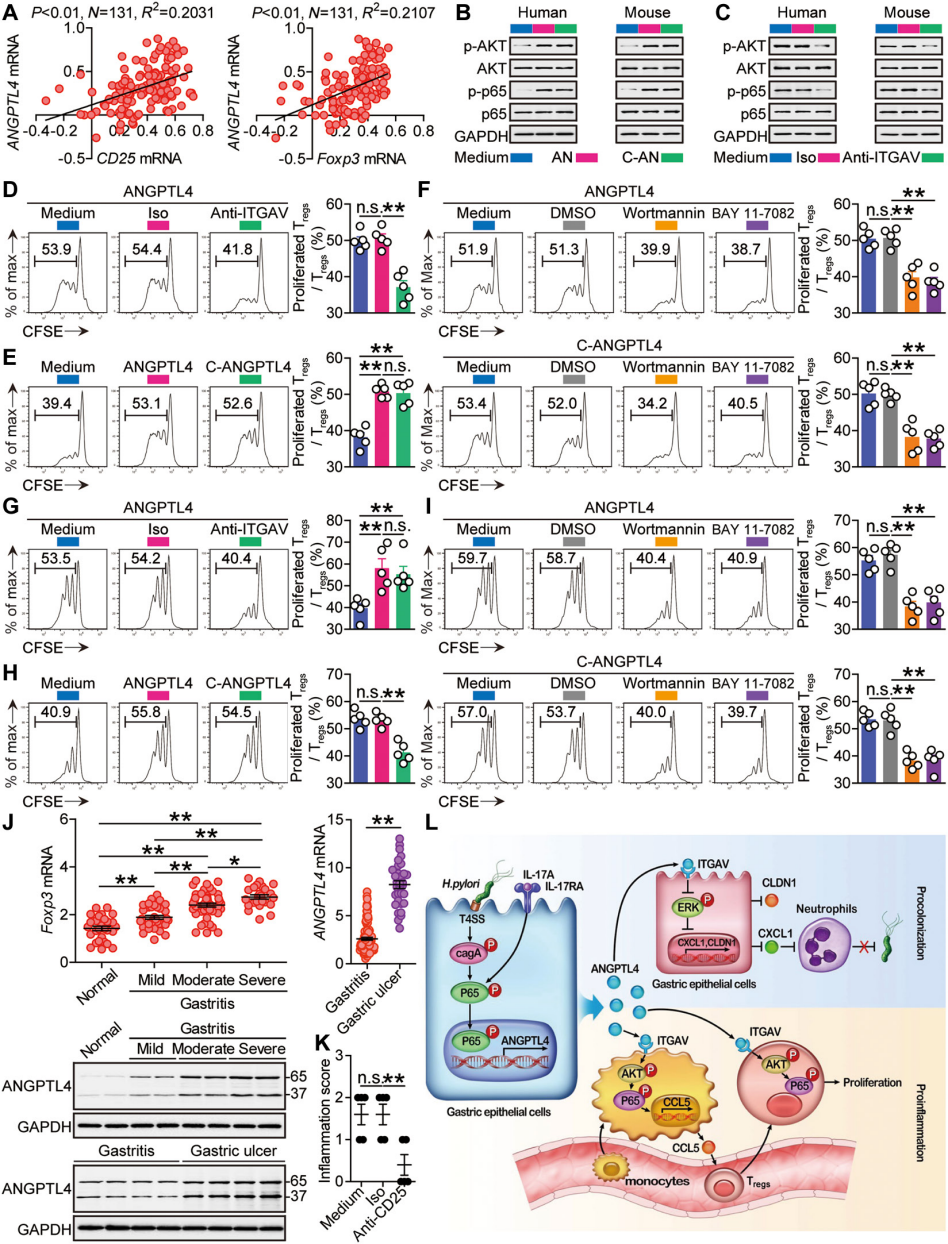
ANGPTL4 promotes T_reg_ proliferation by binding to ITGAV to activate NF-κB, aggravating inflammation during *H. pylori* infection. (A) The correlations between *ANGPTL4* expression and *CD25* expression, and between *ANGPTL4* expression and *Foxp3* expression in gastric mucosa of *H. pylori*-infected patients were analyzed. Results are expressed as log_10_(fold change). (B) T_regs_ were pretreated with anti-ITGAV Abs and then stimulated with ANGPTL4 or cANGPTL4 (1 μg/ml) for 6 h. AKT and p-AKT and p65 and p-p65 proteins were analyzed by Western blot. (C) T_regs_ were pretreated with anti-ITGAV Abs and then stimulated with ANGPTL4 (1 μg/ml) for 6 h. AKT and p-AKT and p65 and p-p65 proteins were analyzed by Western blot. (D to F) CFSE-labeled T_regs_ from *H. pylori*-infected patients were pretreated with anti-ITGAV Abs and cultured with ANGPTL4 (1 μg/ml) for 5 d (D), or were cultured with ANGPTL4 or cANGPTL4 (1 μg/ml) for 5 d (E), or were pretreated with wortmannin or BAY 11-7082 and cultured with ANGPTL4 (1 μg/ml) for 5 d (F). Representative data and statistical analysis of T_reg_ proliferation were shown (*n* = 5). (G to I) CFSE-labeled T_regs_ from WT *H. pylori*-infected mice (12 weeks p.i.) were pretreated with anti-ITGAV Abs and cultured with ANGPTL4 (1 μg/ml) for 5 d (G), or were cultured with ANGPTL4 or cANGPTL4 (1 μg/ml) for 5 d (H), or were pretreated with wortmannin or BAY 11-7082 and cultured with ANGPTL4 (1 μg/ml) for 5 d (I). Representative data and statistical analysis of T_reg_ proliferation were shown (*n* = 5). (J) *Foxp3* expression in gastric mucosa of *H. pylori*-infected patients with mild (*n* = 34), moderate (*n* = 45), and severe inflammation (*n* = 24) and with normal gastric histopathology (*n* = 28) was compared. *ANGPTL4* expression in gastric mucosa of patients with gastritis (*n* = 103) or with gastric ulcer (*n* = 32) was compared. ANGPTL4 proteins in gastric mucosa of *H. pylori*-infected patients with mild, moderate, and severe inflammation and with normal gastric histopathology or in gastric mucosa of patients with gastritis or with gastric ulcer were analyzed by Western blot. (K) The histological scores of inflammation in gastric mucosa of WT mice injected with anti-CD25 Abs or control IgG at 12 weeks p.i. were compared (*n* = 5). (L) Proposed model of cross-talk among *H. pylori*, GECs, monocytes, and T_regs_, facilitated by IL-17A, ANGPTL4, and ITGAV, leading to ANGPTL4-mediated pro-colonization and pro-inflammation in gastric mucosa during *H. pylori* infection. Data are representative of 2 independent experiments. Data are shown as mean ± SEM and analyzed by Student’s *t* test, Mann–Whitney *U* test, and one-way ANOVA. Western blot results are run in parallel and contemporaneously. *P < 0.05, **P < 0.01, n.s. *P* > 0.05 for groups connected by horizontal lines.

Positive correlations between ANGPTL4 and Foxp3 as well as between ANGPTL4 and CD25 within the gastric mucosa of *H. pylori*-infected patients (Fig. 7A), and high expressions of ITGAV on T_regs_ from HPA immune cell dataset (Fig. S16A) implied that ANGPTL4 might have effects on T_regs_ by binding to ITGAV to activate PI3K–AKT–NF-κB signaling pathway [10,29]. ANGPTL4-treated T_regs_ and cANGPTL4-treated T_regs_ showed activated PI3K–AKT–NF-κB signaling pathways (Fig. 7B), which was lost upon when pretreated with neutralizing Abs against ITGAV (Fig. 7C). More importantly, Ab blocking (Fig. 7D) and signaling pathway inhibiting experiments (Fig. 7E and F) showed that ANGPTL4′ binding to ITGAV contributed to the proliferation of human T_regs_ by activating PI3K–AKT–NF-κB. Similar findings were made when we analyzed mouse T_reg_ proliferation (Fig. 7G to I). Collectively, our findings suggest that ANGPTL4 interacts with ITGAV to promote T_reg_ proliferation by activating PI3K–AKT–NF-κB.

Notably, compared to the patients with gastritis, the levels of ANGPTL4 in the patients with gastric ulcer were significantly higher (Fig. 7J), along with the down-regulation of tight junction protein CLDN1 by ANGPTL4 (Figs. 4 and 5) and the decreased transepithelial electrical resistance (TEER) values in ANGPTL4-stimulated AGS cells (Fig. S16F), suggesting potential roles of *H. pylori*-induced ANGPTL4 in the damage of gastric mucosa. Next, within the gastric mucosa of *H. pylori*-infected patients, Foxp3 as well as CD25 were increased and were found to be positively correlated with the severity of gastritis (Fig. 7J and Fig. S17). Moreover in vivo CD25 Ab-mediated T_reg_ depletion showed that, compared to the control mice, there was significant less inflammation in the treated mice (Fig. 7K). Overall, our findings above indicate that, within the *H. pylori*-infected gastric mucosa, ANGPTL4 exerts important roles in aggravating inflammation, which may contribute to the damage of gastric mucosa.

## Discussion

During persistent *H. pylori* infection, gastric stromal cells often produce secreted angiogenic-regulating factors to promote bacterial infection-associated pathology [30,31]. Some of these proteins exhibit both pro-colonization and pro-inflammation roles by regulating chronic inflammatory responses of mononuclear cells rather than granulocytes. Here, we have shown, for the first time, that infection with *H. pylori* elevates host ANGPTL4 expression, which reshapes the gastric environment by promoting the infiltration of mononuclear cells (T_regs_ in our data) rather than granulocytes (neutrophils in our data) into the infected gastric mucosa, directly leading to gastritis progression and bacterial persistence.

Recently, high-throughput RNA sequencing of colorectal cancer cells cultured with *Fusobacterium nucleatum* showed that *ANGPTL4*, as one of the most significantly up-regulated genes, was increased upon infection [32,33]. In our study, we have shown that ANGPTL4 expression is readily induced and expressed upon *H. pylori* infection in the gastric tissues as well as GECs, which is consistent with the other reports about the increased expressions of ANGPTL4 in the lung tissues with influenza pneumonia [19]. ANGPTL4 has been reported to be regulated by several cytokines such as transforming growth factor-β (TGF-β) [34] and IL-1β [35]. In our case of *H. pylori* infection, we identify a novel ANGPTL4 regulating cytokine, IL-17A, which exerts synergistic effects on the induction of ANGPTL4 by activating the NF-κB signaling pathway, where its pathway is similar to other pathogen infection [36]. These data, together with the previous findings on the pathologic functions of IL-17A and T_H_17 cells during *H. pylori* infection [37], emphasizes the important determinants of IL-17A from T_H_17 cells for ANGPTL4 induction within the gastric mucosa. More importantly, increased IL-17A serum levels and gastric T_H_17 cells have been found in *H. pylori*-infected patients with gastric premalignant lesions [38], and *H. pylori* lipoprotein HP1454 can regulate T cell response by shaping T cell receptor signaling [39], together suggesting that T_H_17 cell responses induced by *H. pylori*-associated factors contribute to progressive pathological roles within the gastric mucosa.

Almost all knowledge on the functions of ANGPTL4 derives from the key roles of nANGPTL4 in regulating lipid metabolism and glucose homeostasis [40,41], as well as the key roles of cANGPTL4 in the promotion of vessel permeability and angiogenesis [42,43]. Several conflicting data portrayed ANGPTL4 either as a protumorigenic or proangiogenic molecule [44,45] or as a primarily antiangiogenic and antitumorigenic cytokine [46,47], and recent preclinical reports supported the roles of cANGPTL4 in suppressing tumor growth and angiogenesis and nANGPTL4 in inhibiting angiogenesis and tumor metastasis [18], indicating that the C-terminal and N- terminal fragments of ANGPTL4 could have opposing functions during tumor progression. The disease-promoting roles of ANGPTL4 in *H. pylori*-infected gastric environment could be such a case, in which flANGPTL4 and cANGPTL4 are mainly detected in GECs and they show similar effects on monocytes and T_regs_. Moreover, CLDN1 expression was negatively correlated with ANGPTL4 within the *H. pylori*-infected gastric mucosa of patients and mice along with higher ANGPTL4 in patients with gastric ulcer, suggesting potential roles of gastric mucosal damage of ANGPTL4 in *H. pylori*-associated gastric diseases.

The functional roles of ANGPTL4 are reported to be indicated to involve several mechanisms, and one of which is that ANGPTL4 and cANGPTL4 interact with integrins to decluster proteins of tight junction, leading to cell disruption [48]. Interestingly, this observation is consistent with our findings that tight junction protein CLDN1 of GECs is down-regulated by ANGPTL4 and cANGPTL4. Integrins, as important cell surface receptors, connect intracellular structures to serve important signaling functions. Tumor-derived ANGPTL4 interacts with integrins to regulate cancer progression by triggering the PI3K-associated signaling [49] and partly due to suppression of ERK signaling [46], which is also consistent with our findings as we have shown that integrin signaling pathway involving ERK and PI3K are regulated by ANGPTL4. Moreover, we have further shown that ANGPTL4 and cANGPTL4 suppress the production of CXCL1 from GECs and promote the production of CCL5 from monocytes through direct binding to ITGAV to inhibit ERK and activate PI3K–AKT–NF-κB, respectively. Furthermore, we have shown that ANGPTL4 suppresses CXCL1 production contributing to the increased gastric bacterial colonization, which is due to the impaired neutrophil infiltration that plays important roles in controlling gastric *H. pylori* colonization in our previous report [50]. Our study also identifies pathological roles of ANGPTL4 that contribute to the gastric environmental changes of gastritis featuring preferential infiltration of mononuclear cells (T_regs_ in our data) rather than granulocytes; further, our findings elucidate the molecular mechanisms of ANGPTL4 in promoting T_reg_ chemotaxis and proliferation. Consistent with the pathologic potential of T_regs_ in other diseases [51,52], we here demonstrate the proinflammatory roles of T_regs_ in gastritis induced by *H. pylori* infection.

### Conclusion

Collectively, our data identify ANGPTL4 as a novel pathological host factor that contributes to gastritis as well as promotes *H. pylori* colonization, mostly acting through 2-pronged mechanisms, involving both suppressing gastric neutrophil infiltration and promoting T_reg_ chemotaxis/proliferation, which reshapes host gastric environments with features of infiltration of mononuclear cells more than granulocytes. Importantly, our results together identify a multi-step model of persistent *H. pylori* infection accompanied with chronic gastritis involving cross-talks between *H. pylori*, IL-17A, GECs, ANGPTL4, neutrophils, monocytes, as well as T_regs_ (Fig. 7L). Therefore, targeting the ANGPTL4-associated molecular pathways will be interesting to improve the outcomes of the *H. pylori* infection-associated diseases.

## Methods

### Patients and specimens

The gastric biopsy specimens and blood were collected from 50 uninfected donors, 131 *H. pylori*-infected patients, and 32 gastric ulcer patients who underwent upper esophagogastroduodenoscopy for dyspeptic symptoms at XinQiao Hospital (Table S1). *H. pylori* infection was determined by [^14^C] urea breath test and rapid urease test of biopsy specimens taken from the antrum, and subsequently confirmed by real-time PCR for 16*S* ribosomal DNA (rDNA) and serology test for specific anti-*H. pylori* Abs. For isolation of human primary GECs, fresh nontumor gastric tissues (at least 5 cm distant from the tumor site) were obtained from gastric cancer patients who underwent surgical resection and were determined as *H. pylori*-negative individuals as above at the Southwest Hospital. None of these patients had received chemotherapy or radiotherapy before sampling. Individuals with atrophic gastritis, hypochlorhydria, antibiotics treatment, autoimmune disease, infectious diseases, and multi-primary cancer were excluded.

### Abs and other reagents (Table S2)

### Mice

C57BL/6 *Angptl4*^−*/*−^ mice were generated (Cyagen Biosciences, China). C57BL/6 *Il17a*^−/−^ mice were kindly provided by R. A. Flavell (Yale University, USA). *Il17a*^−/−^*Angptl4*^−/−^ mice and their littermate control (WT) mice were generated by cross-breeding *Il17a*^−/−^ and *Angptl4*^−/−^ mice. Only female mice were used in all experiments except for the chimeric experiments, in which male mice were also used and were free of Abs specific for pathogenic murine viruses; negative for pathogenic bacteria, including *Helicobacter* spp., and parasites; and were maintained under specific pathogen-free conditions in a barrier-sustained facility and provided with sterile food and water [23].

### Bacteria culture and infection of mice with bacteria

*H. pylori* NCTC 11637 (*cagA* positive) (WT *H. pylori*) and *cagA*-knockout mutant *H. pylori* NCTC 11637 (*ΔcagA*) (kindly provided by C. Sasakawa [53,54]) were grown in brain–heart infusion plates containing 10% rabbit blood at 37 °C under microaerophilic conditions. For infecting mouse, bacteria were propagated in Brucella broth with 5% fetal bovine serum (FBS) with gentle shaking at 37 °C under microaerobic conditions. After culture for 1 d, live bacteria were collected and adjusted to 10^9^ colony-forming units (CFU)/ml. The number of bacteria was determined by measuring the optical density at 600 nm (1 OD_600_ = 1 × 10^9^ CFU/ml). The mice were fasted overnight and orogastrically inoculated twice at a 1-d interval with 3 × 10^8^ CFU bacteria. *H. pylori* infection status and *H. pylori*-induced gastritis in murine experiments were confirmed using real-time PCR of *H. pylori* 16*S* rDNA, urease biopsy assays, Warthin–Starry staining, and immunohistochemical staining for *H. pylori* and evaluation of inflammation by hematoxylin and eosin (H&E) staining.

### Generation of BM chimera mice

The following BM chimeric mice were created: male *Il17a*^−/−^ BM→female *Angptl4*^−/−^ mice and male *Angptl4*^−/−^ BM→female *Il17a*^−/−^ mice; or male WT BM→female *Il17a*^−/−^*Angptl4*^−/−^ mice, male *Il17a*^−/−^ BM→female *Il17a*^−/−^*Angptl4*^−/−^ mice, male *Angptl4*^−/−^ BM→female *Il17a*^−/−^*Angptl4*^−/−^ mice, and male *Il17a*^−/−^*Angptl4*^−/−^ BM→female *Il17a*^−/−^*Angptl4*^−/−^ mice; or male WT BM→female WT mice, male WT BM→female *Angptl4*^−/−^ mice, male *Angptl4*^−/−^ BM→female WT mice, and male *Angptl4*^−/−^ BM→female *Angptl4*^−/−^ mice. BM cells were collected from the femurs and tibia of donor mice by aspiration and flushing, and were suspended in phosphate-buffered saline (PBS) at the concentration of 5 × 10^7^/ml. The BM in recipient mice was ablated with lethal irradiation (8 Gy). Then, the animals received intravenously 1.5 × 10^7^ BM cells from donor mice in a volume of 300 μl of sterile PBS under anesthesia. Thereafter, the transplanted BM was allowed to regenerate for 8 weeks before subsequent experimental procedures. To verify successful engraftment and reconstitution of the BM in the host mice, genomic DNA was isolated from tail tissues of each chimera mouse 8 weeks after BM transplantation. Quantitative PCR was performed to detect the *Sry* gene present in the Y chromosome (primers seen in Table S3) and mouse *β2-microglobulin* gene as an internal control. The chimeric rates were calculated on the assumption that the ratio of the *Sry* to *β2-microglobulin* gene was 100% in female recipient mice. We confirmed that the chimeric rates were consistently higher than 90%. After BM reconstitution was confirmed, mice were infected with bacteria as described above.

### ANGPTL4/chemokine/Ab administration

One day after infection with WT *H. pylori* as described above, WT mice were injected intraperitoneally with 25 μg of recombinant mouse ANGPTL4/cANGPTL4, recombinant mouse CXCL1, recombinant mouse CCL5, or anti-mouse CXCL1, anti-mouse CCL5, anti-mouse CCR4, anti-mouse Ly6G, anti-mouse CD25 Abs, or their control IgG (100 μg) and repeated every week until the mice were euthanized.

### Evaluation of bacteria colonization

The mice were euthanized at the indicated times. The stomach was cut open from the greater curvature, and half of the tissue was cut into 4 parts for RNA extraction, DNA extraction, protein extraction, and tissue fixation for immunohistochemistry staining, respectively. DNA of the biopsy specimens was extracted with QIAamp DNA Mini Kit. As previously described [55], *H. pylori* colonization was quantified by measuring *H. pylori*-specific 16*S* rDNA using specific primer and probe (Table S3) by the TaqMan method. The amount of mouse β2-microglobulin DNA in the same specimen was used to normalize the data. According to a previous study [56], the density of *H. pylori* was shown as the number of bacterial genomes per nanogram of host genomic DNA [24]. Another half of stomach was used for isolation of single cells as described below. The isolated single cells were collected and analyzed by flow cytometry staining.

### Evaluation of inflammation

Mice were euthanized at the indicated times. The greater curvature of the stomach was cut to perform H&E staining. The intensity of inflammation was evaluated independently by 2 pathologists according to previous established criteria [23].

### Isolation of single cells from tissues

Fresh tissues were washed 3 times with Hank’s solution containing 1% FBS, cut into small pieces, collected in RPMI 1640 containing 1 mg/ml collagenase IV and 10 mg/ml deoxyribonuclease I, and then mechanically dissociated by using the gentleMACS Dissociator (Miltenyi Biotec). Dissociated cells were further incubated for 0.5 to 1 h at 37 °C under continuous rotation. The cell suspensions were then filtered through a 70-μm cell strainer (BD Labware).

### Gastric organoid cultures

For mouse gastric organoid, gastric tissue single-cell suspensions isolated from uninfected mice were mixed in Matrigel and cultured in 24-well plates. As previously described [57], the mixture was coagulated in a 37 °C incubator for 10 min before adding culture medium containing recombinant mouse growth factors [Wnt3a (100 ng/ml), Noggin (100 ng/ml), R-Spindin-1 (1 μg/ml), epidermal growth factor (EGF) (50 ng/ml), FGF-10 (100 ng/ml), Gastrin I (10 nM)] and various value-added components (1× N-2 supplement, 10% GlutaMAX supplement to advanced Dulbecco’s modified Eagle’s medium/F12). Special notes were to supplement the culture medium with Y-27632 (10 mM) in the first 2 d of cultivation to avoid loss of nest cell apoptosis. The culture medium was changed once every 3 to 5 d, and the passage of organoids was performed in a 1:4 ratio depending on the growth situation after 2 weeks. For human gastric organoid, gastric nontumor (at least 5 cm distant from the gastric cancer site) tissues were obtained from *H. pylori*-uninfected gastric cancer donors who underwent surgical resections. As previously described [58], the fresh gastric mucosa layer of the collected tissues was separated and cut into small pieces (2 mm^3^) and then digested with collagenase and hyaluronidase for 1 h at 37 °C. The supernatant was filtered with a 100-μm cell filter, resuspended in appropriate precooled Matrigel, and added to the center of each well of 12-well plates until the Matrigel was completely solidified. Then, the pre-prepared medium containing various growth factors (Biogenous) was added. The culture medium was changed once every 3 to 5 d, and the passage of organoids was performed in a 1:4 ratio depending on the growth situation after 2 weeks.

### Cell/tissue/organoid culture and stimulation

Primary GECs were purified from gastric tissue single-cell suspensions from uninfected donors or mice with a MACS column purification system using anti-human or mouse CD326 magnetic beads (Miltenyi Biotec). The sorted primary GECs were used only when their viability was determined >90% and their purity was determined >95%. The cells were cultured in complete RPMI 1640 medium supplemented with 10% FBS in a humidified environment containing 5% CO_2_ at 37 °C. Human GEC lines (AGS cells, GES-1 cells, HGC-27 cells, and SGC-7901 cells) were obtained from the American Type Culture Collection (Manassas, VA, USA). Human GEC lines, primary GECs, or primary gastric mucosa tissues from uninfected donors or mice were infected with WT *H. pylori* or *ΔcagA* at a multiplicity of infection (MOI) of 100 for 24 h. Human and mouse gastric organoids were infected with WT *H. pylori* or *ΔcagA* (MOI = 100) for 24 h, or infected with WT *H. pylori* (MOI = 100) in the presence or absence of IL-17A (100 ng/ml) for 24 h. AGS cells were also infected with WT *H. pylori* (MOI = 100) in the presence or absence of IL-17A (50, 100, 200 ng/ml) for 24 h, or infected with WT *H. pylori* (MOI = 100) in the presence or absence of IL-1β, IL-2, IL-3, IL-4, IL-6, IL-8, IL-10, IL-12, IL-17A, IL-17F, IL-22, IL-23, IL-33, TNF-α, TGF-β, or interferon-γ (IFN-γ) (100 ng/ml) for 24 h, or infected with WT *H. pylori* (MOI = 100) and/or IL-17A (100 ng/ml) in the presence or absence of neutralizing Abs against IL-17A (20 μg/ml) and/or IL-17RA (20 μg/ml) for 24 h. AGS cells, primary GECs, or primary gastric mucosa tissues were also infected with WT *H. pylori* at different MOI (24 h) or at the indicated time points (MOI = 100) or infected with WT *H. pylori* (MOI = 100) in the presence or absence of IL-17A (100 ng/ml) for 24 h. AGS cells were infected with WT *H. pylori* (MOI = 100) in the presence or absence of ANGPTL4 (100 ng/ml) for 24 h. AGS cells were stimulated with ANGPTL4, cANGPTL4, or nANGPTL4 (1 μg/ml) for 24 h or were stimulated with ANGPTL4/cANGPTL4 (1 μg/ml) for 24 h in the presence or absence of neutralizing Abs against ITGAV (20 μg/ml) or control IgG (20 μg/ml). Human or mouse monocytes from blood of *H. pylori*-infected donors or WT *H. pylori*-infected mice (12 weeks p.i.) were sorted by using anti-CD14 magnetic beads (STEMCELL Technologies). Monocytes were stimulated with ANGPTL4/cANGPTL4 (1 μg/ml) for 6 or 24 h in the presence or absence of neutralizing Abs against ITGAV (20 μg/ml) or control IgG (20 μg/ml). Human (CD45^+^CD19^−^CD56^−^CD3^+^CD4^+^CD25^+^) or mouse (CD45^+^CD19^−^CD49b^−^CD3^+^CD4^+^CD25^+^) T_regs_ from blood of *H. pylori*-infected donors or WT *H. pylori*-infected mice (12 weeks p.i.) were sorted by fluorescence-activated cell sorting (FACS) (FACSAria III; BD Biosciences) and then were further stimulated with ANGPTL4/cANGPTL4 (1 μg/ml) for 6 or 24 h in the presence or absence of neutralizing Abs against ITGAV (20 μg/ml) or control IgG (20 μg/ml). In some cases, AGS cells were transfected with plasmids *cagA*-pcDNA3.1 or pcDNA3.1 (control vector) by using Lipofectamine 2000 according to the manufacturer’s protocols for 48 h. For signal pathway inhibition experiments, AGS cells, monocytes, and T_regs_ were pretreated with PP2, SP600125, SB203580, wortmannin, AG490, BAY 11-7082, U0126 (20 μM), or dimethyl sulfoxide (DMSO) for 2 h. For ANGPTL4 inhibition experiments, AGS cells were pretreated with *ANGPTL4* siRNA or nonspecific control siRNA (NC) (40 nM) or Lipofectamine 2000 only (mock) for 24 h. For transwell assays, AGS cells were added to the lower chamber, and WT *H. pylori* (MOI = 100) were placed into the lower or the upper chambers of transwells (0.4-μm pore) and then incubated for 24 h. After coculture, the cells were collected for microarray, immunofluorescence, real-time PCR, and Western blot, and the culture supernatants were harvested for enzyme-linked immunosorbent assay (ELISA).

### Neutrophil chemotaxis assay

Human neutrophils (CD45^+^CD11b^+^CD15^+^CD66b^+^) or mouse neutrophils (CD45^+^CD90^−^CD19^−^CD49b^−^Siglec-F^−^CD11b^+^Ly6G^+^) from blood of *H. pylori*-infected donors or WT *H. pylori*-infected mice (12 weeks p.i.) were sorted by FACS (FACSAria III; BD Biosciences). AGS cells were pretreated with *ANGPTL4* siRNA or nonspecific control siRNA (NC) (40 nM) or Lipofectamine 2000 only (mock) for 24 h and then stimulated with WT *H. pylori* or *ΔcagA* (MOI = 100) for 24 h. The culture supernatants were collected and used as source of chemoattractants in a human neutrophil chemotaxis assay. In another set of experiments, mouse primary GECs were purified from gastric tissue single-cell suspensions with a MACS column purification system using anti-mouse CD326 magnetic beads (Miltenyi Biotec) and then stimulated with WT *H. pylori* or *ΔcagA* (MOI = 100) for 24 h. The culture supernatants were collected and used as source of chemoattractants in a mouse neutrophil chemotaxis assay. In a chemotaxis assay, sorted cells (1 × 10^5^) were transferred into the upper chambers of transwells (5-μm pore). CXCL1 (100 ng/ml) and culture supernatants from various cultures were placed in the lower chambers. After 6-h culture, migration was quantified by counting cells in the lower chamber and cells adhering to the bottom of the membrane. In some cases, blocking Abs for mouse CXCL1 (20 μg/ml) or control IgG (20 μg/ml) were added into culture supernatants.

### T_reg_ chemotaxis assay

Human (CD45^+^CD19^−^CD56^−^CD3^+^CD4^+^CD25^+^) or mouse (CD45^+^CD19^−^CD49b^−^CD3^+^CD4^+^CD25^+^) T_regs_ from blood of *H. pylori*-infected donors or WT *H. pylori*-infected mice (12 weeks p.i.) were sorted by FACS (FACSAria III; BD Biosciences). Human or mouse monocytes from blood of *H. pylori*-infected donors or WT *H. pylori*-infected mice (12 weeks p.i.) were sorted by using anti-CD14 magnetic beads (STEMCELL Technologies). Monocytes were stimulated with ANGPTL4/cANGPTL4 (1 μg/ml) for 24 h in the presence or absence of neutralizing Abs against ITGAV (20 μg/ml) or control immunoglobulin G (IgG) (20 μg/ml). For signal pathway inhibition experiments, monocytes were pretreated with wortmannin, BAY 11-7082 (20 μM), or DMSO for 2 h before ANGPTL4/cANGPTL4 stimulation. The culture supernatants were collected and used as source of chemoattractants in T_reg_ chemotaxis assays. In a chemotaxis assay, sorted cells (1 × 10^5^) were transferred into the upper chambers of transwells (5-μm pore). CCL5 (100 ng/ml) and culture supernatants from various cultures were placed in the lower chambers. After 6-h culture, migration was quantified by counting cells in the lower chamber and cells adhering to the bottom of the membrane. In some cases, blocking Abs for CCL5 (20 μg/ml) or control IgG (20 μg/ml) were added into culture supernatants, and blocking Abs for CCR4 (20 μg/ml) or control IgG (20 μg/ml) were added into cell suspensions and incubated for 2 h before chemotaxis assay.

### T_reg_ proliferation assay

Human (CD45^+^CD19^−^CD56^−^CD3^+^CD4^+^CD25^+^) or mouse (CD45^+^CD19^−^CD49b^−^CD3^+^CD4^+^CD25^+^) T_regs_ from blood of *H. pylori*-infected donors or WT *H. pylori*-infected mice (12 weeks p.i.) were sorted by FACS (FACSAria III; BD Biosciences) and then labeled with carboxyfluorescein succinimidyl ester (CFSE) and further cultured (1 × 10^5^ cells/well) with ANGPTL4/cANGPTL4 (1 μg/ml) in 200 μl of RPMI 1640 medium containing recombinant IL-2 (20 IU/ml), anti-CD3 (2 μg/ml), and anti-CD28 (1 μg/ml) Abs in the presence or absence of neutralizing Abs against ITGAV (20 μg/ml) or control IgG (20 μg/ml). For signal pathway inhibition experiments, T_regs_ were pretreated with wortmannin, BAY 11-7082 (20 μM), or DMSO for 2 h before culture. After a 5-d incubation, the cells were collected for flow cytometry.

### Luciferase reporter assay

Promoter constructs containing the region from −3,000 to −1 of the *ANGPTL4* gene were amplified from human genomic DNA by PCR. The amplified full length or fragments were cloned into the NheI and HindIII sites of the pGL3-basic vector, respectively, by Sangon Biotech (Shanghai, China). For luciferase reporter assay, cells were seeded in 24-well plates and transfected when reaching approximately 80% confluence with the constructed luciferase reporter vector for 4 h. Lipofectamine 2000 was used to transfect AGS cells according to the manufacturer’s protocols. Luciferase activity was measured to assess promoter activity after WT *H. pylori* or Δ*cagA* infection (MOI = 100) for 24 h or after *cagA*-pcDNA3.1 plasmid transfection (pretreated with or without BAY 11-7082) for 48 h by the Dual-Luciferase Reporter assay following the manufacturer’s protocol. Luciferase activity was normalized to Renilla luciferase activity.

### Chromatin immunoprecipitation

AGS cells were infected with WT *H. pylori* or *ΔcagA* (MOI = 100) for 24 h or transfected with *cagA*-pcDNA3.1 for 48 h (pretreated with or without BAY 11-7082). The cells were then treated at room temperature for 10 min with 1% formaldehyde in cell culture medium. Glycine (11% in medium) solution was then gently mixed in at room temperature for 5 min to terminate cross-linking. The cells were washed twice with ice-cold PBS and palleted at 3,000*g* for 5 min. Membrane Extraction Buffer containing protease/phosphatase inhibitors was then added to each palleted sample. The cell lysates were pulse-sonicated on ice; supernatants containing the digested chromatin were collected into 2 tubes for input and IP. Anti-p65 Abs or control IgG was added, and IP reactions were conducted overnight at 4 °C with agitation. ChIP grade protein A/G magnetic beads were then added to each IP reaction. Two hours later, beads were collected and washed, and bounded IP materials eluted with 5 M NaCl containing 20 μg/ml proteinase K. The cross-linking was reversed by heating up to 65 °C for 1.5 h, and DNA was purified. Purified DNA samples were analyzed by PCR with designed primers (Table S5).

### Generation of AGS cells expressing the flag-tagged ANGPTL4 (ANGPTL4-flag)

The full length of human ANGPTL4 CDS [National Center for Biotechnology Information (NCBI) gene ID: 51129], with the Flag tag just behind the start codon, was chemically synthesized and inserted into the pLVX-mCMV-ZsGreen1-Puro lentivirus vector at the 2 restriction sites EcoRI and BamHI. The above reconstructed plasmids were transiently transfected into the AGS cells, and IP and Western blotting were applied to confirm its overexpression. Briefly, the adherent cells were lysed by precold IP dilution buffer (20 mM tris-HCl, 2 mM EDTA, 1% Triton-X100, 150 mM NaCl) supplemented with phenylmethylsulfonyl fluoride (PMSF) and protein inhibitor complex (PIC) on ice. The cellular debris was removed by centrifugation at 12,000 rpm at 4 °C for 10 min, and the supernatant was maintained for the subsequent IP. In detail, the total cell lysis was equally divided into 2 parts, which were incubated with mouse anti-Flag monoclonal Ab or mouse IgG isotype, respectively. After gentle agitation at 4 °C overnight, pretreated Pierce Protein G Magnetic beads were added into the lysate samples and agitated at 4 °C for 4 h. Next, the immunoprecipitated protein complexes were enriched using a magnetic separator, collected in elution buffer (10% SDS, 0.5 M EDTA, 1 M tris-HCl), and validated by Western blotting.

### MS analysis

The samples were sent to Sangon Biotech (Shanghai, China) for MS analysis. In brief, the sample was digested in-gel and then analyzed by on-line nanospray liquid chromatography (LC)–MS/MS on Q Exactive HF mass spectrometer (Thermo Fisher Scientific, USA) coupled to an EASY-nanoLC 1000 system (Thermo Fisher Scientific, USA). The peptides (3 μl) were loaded (analytical column: Acclaim PepMap C18, 75 μm × 25 cm) and separated with a 60-min gradient. The column flow rate was maintained at 300 nl/min with the column temperature controlled at 40 °C. The electrospray voltage of 2 kV versus the inlet of the mass spectrometer was used. The mass spectrometer was run under data-dependent acquisition mode and automatically switched between MS and MS/MS mode. Tandem mass spectra were processed by PEAKS Studio version X+ (Bioinformatics Solutions Inc., Waterloo, Canada). PEAKS DB was set up to search the uniprot_homo_sapiens_201907 database (version 201907, 20414 entries) assuming trypsin as the digestion enzyme.

### IP assay

IP assay was performed using a Pierce Classic Magnetic IP/Co-IP Kit following the manufacturer’s protocol. Whole-cell extracts of AGS cells expressing ANGPTL4-Flag were lysed in IP Lysis/Wash Buffer in the presence of protease inhibitor. After centrifugation for 10 min at 13,000*g*, supernatants were collected and incubated with anti-Flag Abs or control IgG at 4 °C overnight. After overnight incubation, the protein A/G magnetic beads (washed 3 times with IP Lysis/Wash Buffer) were incubated with total cell extracts with gentle shaking for 1 h at room temperature. Then, the beads were washed 3 times with IP Lysis/Wash Buffer and resuspended in 50 μl of 1% (w/v) SDS sample buffer and boiled at 97 °C for 10 min. The proteins were separated by SDS-PAGE (polyacrylamide gel electrophoresis) (10% SDS) and transferred to a polyvinylidene difluoride (PVDF) membrane for Western blot analysis.

### Immunohistochemistry

Paraformaldehyde-fixed and paraffin-embedded samples were cut into 5-μm sections. For immunohistochemical staining, the sections were incubated with rabbit anti-human/mouse ANGPTL4, rabbit anti-mouse Ly6G, or rabbit anti-mouse Foxp3, followed by horseradish peroxidase (HRP)-conjugated anti-rabbit IgG and later its substrate diaminobenzidine. All the sections were finally counterstained with hematoxylin and examined using a microscope (Nikon Eclipse 80i; Nikon).

### Immunofluorescence

Human gastric organoids were infected with WT *H. pylori* or *ΔcagA* (MOI = 100) for 24 h and then fixed. AGS cells were treated with ANGPTL4 (1 μg/ml) at 4 °C for 3 h and then fixed. Paraformaldehyde-fixed cryostat tissue sections, gastric organoid sections, or AGS cells were washed in PBS, blocked for 30 min with 20% goat serum in PBS, and stained for ANGPTL4 and PGC, ANGPTL4 and Epcam, ANGPTL4 and ITGAV, and CD14 and CCL5. Slides were examined with a confocal fluorescence microscope (LSM 510 META, Zeiss).

### Real-time PCR

DNA of the biopsy specimens was extracted with QIAamp DNA Mini Kit and RNA of biopsy specimens, and cultured cells were extracted with TRIzol reagent. The RNA samples were reverse-transcribed into cDNA with PrimeScript RT reagent Kit. Real-time PCR was performed on an IQ5 (Bio-Rad) with Real-time PCR Master Mix according to the manufacturer’s specifications. The mRNA expression was measured using the TaqMan and/or SYBR Green method with the relevant primers (Table S3). For mice, mouse *β2-microglobulin*/*β-actin* mRNA level served as a normalizer, and its level in the unstimulated/uninfected cells or stomach/cells of uninfected or WT mice served as a calibrator. For human, human *GAPDH* mRNA level served as a normalizer, and its level in the unstimulated/uninfected/NC-treated cells or stomach/cells of uninfected donors served as a calibrator. The relative gene expression was expressed as fold change of relevant mRNA calculated by the ΔΔCt method.

### Flow cytometry

Cell surface markers were stained with specific or isotype control Abs. For intracellular molecule measurements, the cells were stimulated for 5 h using Leukocyte Activation Cocktail. Intracellular cytokine staining was performed after fixation and permeabilization using Perm/Wash solution. Then, the cells were analyzed by multicolor flow cytometry on FACSCanto (BD Biosciences). Data were analyzed with FlowJo (TreeStar) or FACSDiva software (BD Biosciences).

### ELISA

Isolated human and mouse gastric tissues were homogenized in 1-ml sterile Protein Extraction Reagent and centrifuged. Tissue supernatants were collected for ELISA. Concentrations of ANGPTL4, IL-17A, CXCL1, and CCL5 in the tissue/cell supernatants were determined using ELISA kits according to the manufacturer’s instructions.

### Western blot analysis

Western blots were performed on 10% to 15% SDS-PAGE gel-transferred PVDF membranes with equivalent amounts of cell or tissue lysate proteins for each sample. Five percent skim milk was used for blocking the PVDF membranes. Mouse ANGPTL4, p65, p-p65, AKT, p-AKT, and CLDN1 were detected with rabbit anti-ANGPTL4 Abs, rabbit anti-p65 Abs, rabbit anti-p-p65 Abs, rabbit anti-AKT Abs, rabbit anti-p-AKT Abs, and mouse anti-CLDN1 Abs; human ANGPTL4, p65, p-p65, AKT, p-AKT, ITGAV, and CLDN1 were detected with rabbit anti-ANGPTL4 Abs, rabbit anti-p65 Abs, rabbit anti-p-p65 Abs, rabbit anti-AKT Abs, rabbit anti-p-AKT Abs, mouse anti-ITGAV Abs, and mouse anti-CLDN1 Abs, respectively. This was followed by incubation with HRP-conjugated secondary Abs. Bound proteins were visualized using Super ECL Plus Western Blotting Kit (Bioground, China).

### Transepithelial electrical resistance (TEER) measurements

AGS cells were seeded in 12-well Transwell plates (8.0-μm pore) considering an initial seeding of 150,000 cells/well to achieve a 60 to 80% confluence after a 24-h incubation at 37 °C. After this incubation, AGS cells were stimulated with ANGPTL4 (1 μg/ml) for 24 h. As previously described [59], TEER measurements were then performed using an EVOM2 Epithelial Voltohmeter with an STX2 electrode, and TEER values were further calculated.

### RNA sequencing

Total RNA from ANGPTL4-, cANGPTL4-, or nANGPTL4-stimulated AGS cells (1 μg/ml, 24 h) were extracted using TRIzol Reagent. Then, the samples were sent to Guangzhou Epibiotek Co. Ltd. (Guangzhou, China) for RNA sequencing. The concentration and quality of RNA samples were determined by the NanoDrop 2000 spectrophotometer (NanoDrop Technologies, USA), and then VAHTS Stranded mRNA-seq Library Prep Kit for Illumina V2 (Vazyme Biotech, NR612-02) was used for library preparation according to the instructions. RNA libraries for sequencing were performed on Illumina NovaSeq 6000 platform. Subsequently, 150–base pair (bp) paired-end reads were mapped to the reference human genome build GRCh38/hg38 by using HISAT2. The reads that mapped the genome were calculated using HTSeq. Differential expression analysis presented here has been performed with edgeR package. Significant differential expression genes [log_2_(fold change) >0.32 or <−0.32 and *P* < 0.05) were screened. GO and Kyoto Encyclopedia of Genes and Genomes (KEGG) pathway enrichment analyses were conducted by clusterProfiler R package. Hierarchically clustered heatmaps and volcano plots were visualized by heatmap R package and ggplot2 R package.

### Microarray experiments

Gene expression profiles of WT *H. pylori*-infected and uninfected AGS cells were analyzed with the human Exon 1.0 ST GeneChip (Affymetrix), strictly following the manufacturer’s protocol. Microarray experiments were performed at the Genminix Informatics (China) with the microarray service certified by Affymetrix.

### Gene expression data analysis

To analyze the expression of ANGPTL4 in *H. pylori* infection and *H. pylori*-associated gastritis, we used 2 public datasets (GSE60427 and GSE60662) of clinic samples of *H. pylori*-associated gastritis patients from the GEO datasets (https://www.ncbi.nlm.nih.gov/gds/). All clinic samples from these 2 public datasets (GSE60427 and GSE60662) were infected by *H. pylori*. To execute the background adjustment for the raw data of the datasets from Affymetrix, the “Affy” package in R language was used in Robust Multichip Analysis algorithm [60]. To identify the genes with significant clinical value for *H. pylori*-associated gastritis, 12 cases of “Bhutan gastritis” and 4 cases of “Bhutan normal” from “Series Matrix” of GSE60427 as well as 12 cases of “DR gastritis” and 4 cases of “DR normal” from “Series Matrix” of GSE60427 were selected. The DEGs between “Bhutan gastritis” and “Bhutan normal” groups as well as between “DR gastritis” and “DR normal” groups were identified by using the “limma” package. In addition, 12 cases of “gastritis” and 4 cases of “control” from “Series Matrix” of GSE60662 were also selected. The DEGs between “gastritis” and “control” groups were also identified by using the “limma” package. Statistical significance for each probe set was determined using *P* value. All data were analyzed using R software (R 4.2.1; https://www. r-project.org/).

### PPI network analysis

To predict the interaction pattern of DEGs in *H. pylori* infection and *H. pylori*-associated gastritis, the PPI of DEGs was constructed with a confidence score of ≥0.7 from the STRING12.0 database and then was merged and visualized by Cytoscape software (version 3.7.2). We removed the protein nodes with no interactions with other proteins. Moreover, to identify which genes may be co-regulated to other genes, we conducted coexpression analysis of all genes to calculate Pearson correlation of pair-to-pair genes with the screening criteria (*P* < 0.01 and correlation > 0.8). In the PPI network, the genes served as the nodes and the edges represented the associated interactions. The connectivity degree of each node indicates the number of interactions of the corresponding gene.

### Statistical analysis

Results are expressed as mean ± SEM. Student’s *t* test was generally used to analyze the differences between 2 groups, but when the variances differed, the Mann–Whitney *U* test was used. Inflammation score data were analyzed by the Mann–Whitney *U* test. For multiple comparisons, the one-way analysis of variance (ANOVA) was used. Correlations between parameters were assessed using Pearson correlation analysis and linear regression analysis, as appropriate. SPSS statistical software (version 13.0) was used for all statistical analysis. All data were analyzed using 2-tailed tests, and *P* < 0.05 was considered statistically significant. Raw data from each array were analyzed using TwoClassDif.

## Ethical Approval

All breeding and experiments were undertaken with the review and approval from the Animal Ethical and Experimental Committee of Third Military Medical University (AMUWEC20208020) and the Animal Ethical and Experimental Committee of The General Hospital of Western Theater Command (2024EC2-ky015). The experiments involving human samples were approved by the Ethics Committee of XinQiao Hospital and Southwest Hospital of Third Military Medical University (2021-148-01). The written informed consent was obtained from each subject.

## Data Availability

The human RNA-sequencing data generated in this study have been deposited in the NCBI GEO dataset under accession code GSE266891. The human microarray data generated in this study have been deposited in the NCBI GEO dataset under accession code GSE264263. The publicly available human gastric tissue data used in this study are available in the NCBI GEO database under accession code GSE60427 and GSE60662. All data needed to evaluate the conclusions in the paper are present in the paper and/or the Supplementary Materials. Additional data are available from authors upon request.
