# Supplementary material for: Helicobacter pylori-Induced Angiopoietin-Like 4 Promotes Gastric Bacterial Colonization and Gastritis
Source: Research (Wash D C). 2024 Jul 17;7:0409. doi: 10.34133/research.0409 (PMC11254415; doi:10.34133/research.0409)
Supplement: Supplementary 1 — Figs. S1 to S17 Tables S1 to S8 [file research.0409.f1.zip › revised Supplementary Figures(R1).pdf]

Fig. S1

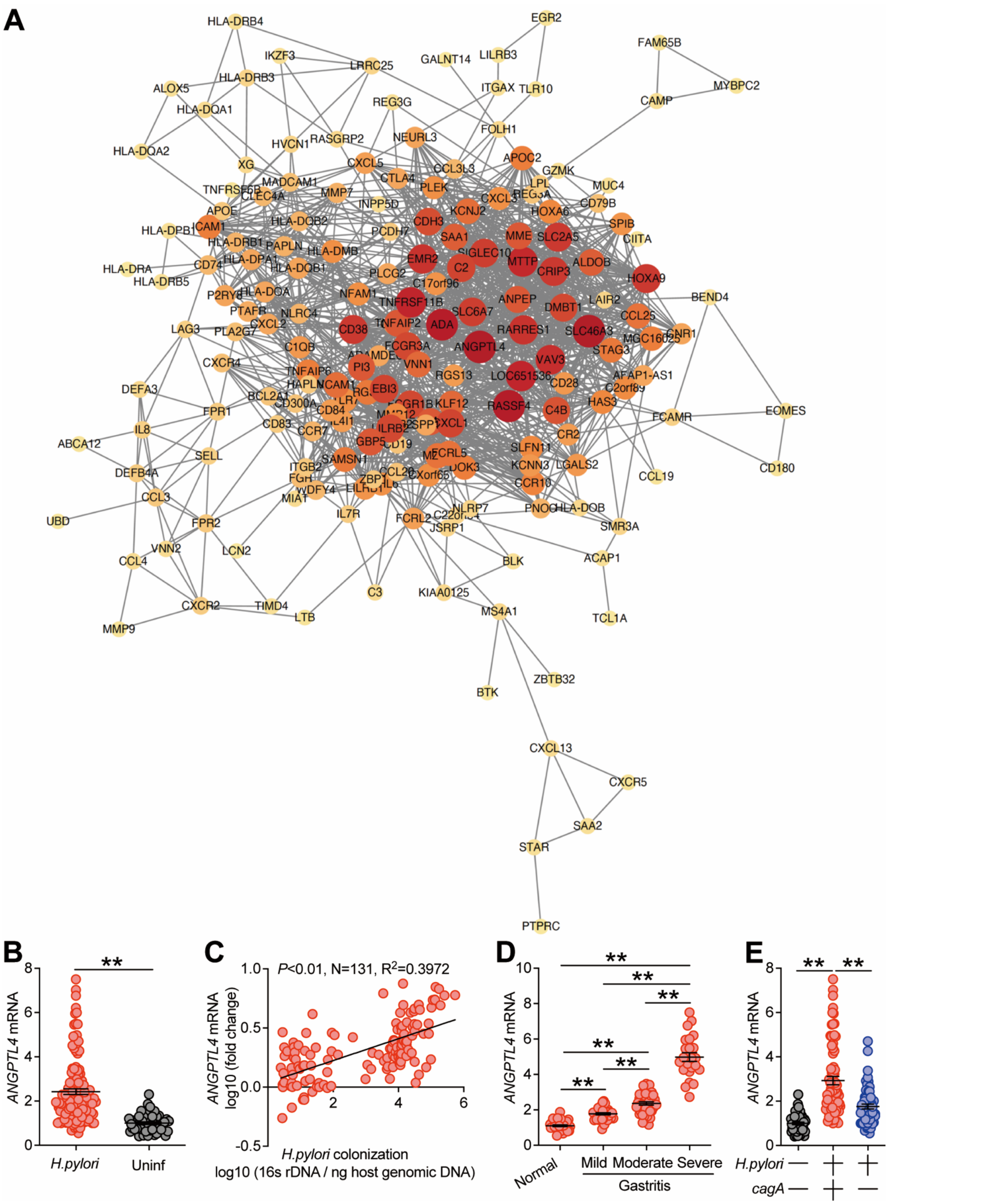

ANGPTL4 is increased in gastric mucosa of *H. pylori*-infected patients and mice. (A) PPI network analysis of the 226 significantly upregulated DEGs in overlapping 3 cohorts (GSE60427_Bhutan, GSE60427_DR and GSE60662) was performed, and the genes with the significance levels (P<0.01 and correlation>0.8) were shown in PPI network. (B) *ANGPTL4* expression in gastric mucosa of *H. pylori*-infected (n=131) and

uninfected donors (n=50) was compared. (C) The correlation between *ANGPTL4* expression and *H. pylori* colonization in gastric mucosa of *H. pylori*-infected patients was analyzed. (D) *ANGPTL4* expression in gastric mucosa of *H. pylori*-infected patients with mild (n=34), moderate (n=45), severe inflammation (n=24), and with normal gastric histopathology (n=28) was compared. (E) *ANGPTL4* expression in gastric mucosa of $cagA^+$ *H. pylori*-infected (n=74), $cagA^-$ *H. pylori*-infected (n=57), and uninfected donors (n=50) was compared. Data are shown as mean ± SEM and analyzed by Student *t* test, Mann-Whitney U test and 1-way ANOVA. *$P<0.05$, **$P<0.01$ for groups connected by horizontal lines.

Fig. S2

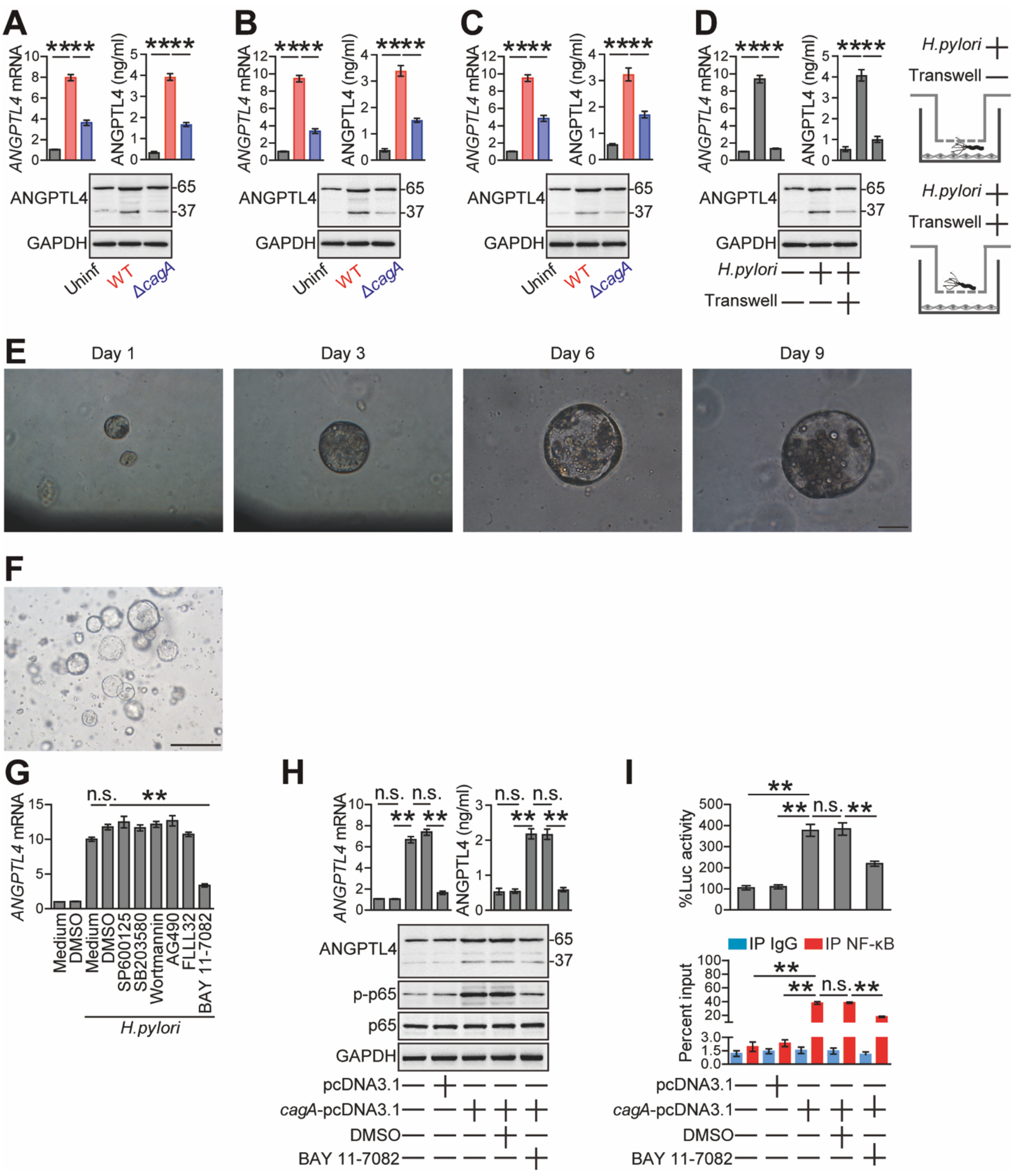

H. pylori stimulates gastric epithelial cells (GECs) to produce ANGPTL4. (A-C) *ANGPTL4* gene and ANGPTL4 protein expressions in WT *H. pylori*-infected, *ΔcagA*-infected, and uninfected GES-1 cells (A), HGC-27 cells (B) and SGC-7901 cells (C) (MOI=100, 24 h) were analyzed by real-time PCR, ELISA and western blot (n=5). (D) *ANGPTL4* gene and ANGPTL4 protein expressions in AGS cells infected with WT *H. pylori* (MOI=100, 24 h) were assessed by a transwell assay and analyzed by real-time PCR, ELISA and western blot (n=5) as descried in the Methods. (E) Representative example of a growing mouse gastric organoid. Scale bars: 50 microns. (F) Representative morphology of human gastric organoids. Scale bars: 200 microns. (G) AGS cells were pre-treated with signal pathway inhibitors and then infected with WT *H. pylori* (MOI=100) for 24 h.

ANGPTL4 gene expression in AGS cells was analyzed by real-time PCR (n=5). (H) AGS cells were transfected with plasmids pcDNA3.1 or *cagA*-pcDNA3.1 for 24 h, then treated with or without BAY 11-7082 for 2 h and cultured for an additional 24 h. *ANGPTL4* gene expression and ANGPTL4, p65 and p-p65 proteins were analyzed by real-time PCR, ELISA and western blot (n=5). (I) AGS cells were co-transfected with *ANGPTL4*-luc construct and *cagA*-pcDNA3.1 (pre-treated with or without BAY 11-7082) or pcDNA3.1 for 48 h. Luciferase activity was measured to assess *ANGPTL4* promoter activity (n=5). ChIP assay in AGS cells transfected with plasmids *cagA*-pcDNA3.1 (pre-treated with or without BAY 11-7082) or pcDNA3.1, followed by PCR with primers designed for NF-κB binding site of *ANGPTL4* promoter region (n=5). Data are representative of 2 independent experiments. Data are shown as mean ± SEM and analyzed by Student *t* test, Mann-Whitney U test and 1-way ANOVA. Western blot results are run in parallel and contemporaneously. **P*<0.05, ***P*<0.01 for groups connected by horizontal lines.

Fig. S3

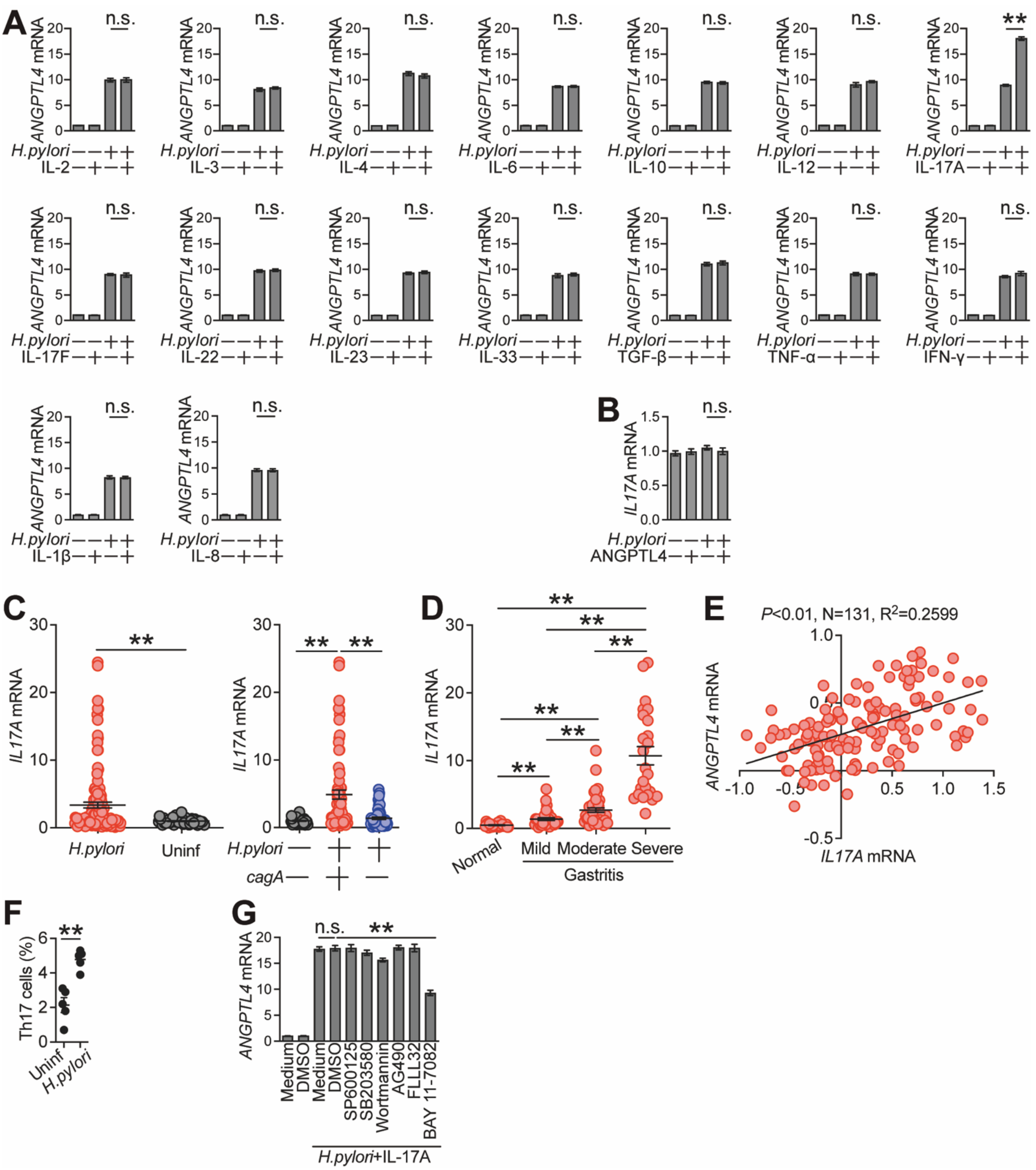

H. pylori and IL-17A synergistically induce ANGPTL4. (A) *ANGPTL4* gene expression in AGS cells infected with WT *H. pylori* (MOI=100) in the presence or absence of IL-1β, IL-2, IL-3, IL-4, IL-6, IL-8, IL-10, IL-12, IL-17A, IL-17F, IL-22, IL-23, IL-33, TNF-α, TGF-β or IFN-γ (100 ng/ml) (24 h) was analyzed by real-time PCR (n=5). (B) *IL17A* gene expression in AGS cells infected with WT *H. pylori* (MOI=100) in the presence or absence of ANGPTL4 (100 ng/ml) (24 h) was analyzed by real-time PCR (n=5). (C) *IL17A* expression in gastric mucosa of *H. pylori*-infected (n=131) and uninfected donors (n=50), or in gastric mucosa of *cagA*$^{+}$ *H.*

pylori-infected (n=74), *cagA*$^-$ *H. pylori*-infected (n=57), and uninfected donors (n=50) was compared. (D) *IL17A* expression in gastric mucosa of *H. pylori*-infected patients with mild (n=34), moderate (n=45), severe inflammation (n=24), and with normal gastric histopathology (n=28) was compared. (E) The correlation between *ANGPTL4* expression and *IL17A* expression in gastric mucosa of *H. pylori*-infected patients was analyzed. Results are expressed as log10(fold change). (F) The levels of Th17 cells in gastric mucosa of uninfected or WT *H. pylori*-infected WT mice at 12 week p.i. were compared (n=5). Results are expressed as the percentage of Th17 cells among CD4$^+$ T cells. (G) AGS cells were pre-treated with signal pathway inhibitors and then infected with WT *H. pylori* (MOI=100) in the presence of IL-17A (100 ng/ml) (24 h). *ANGPTL4* gene expression in AGS cells was compared (n=5). Data are representative of 2 independent experiments. Data are shown as mean ± SEM and analyzed by Student *t* test, Mann-Whitney U test and 1-way ANOVA. **P*<0.05, ***P*<0.01, n.s. *P*>0.05 for groups connected by horizontal lines.

Fig. S4

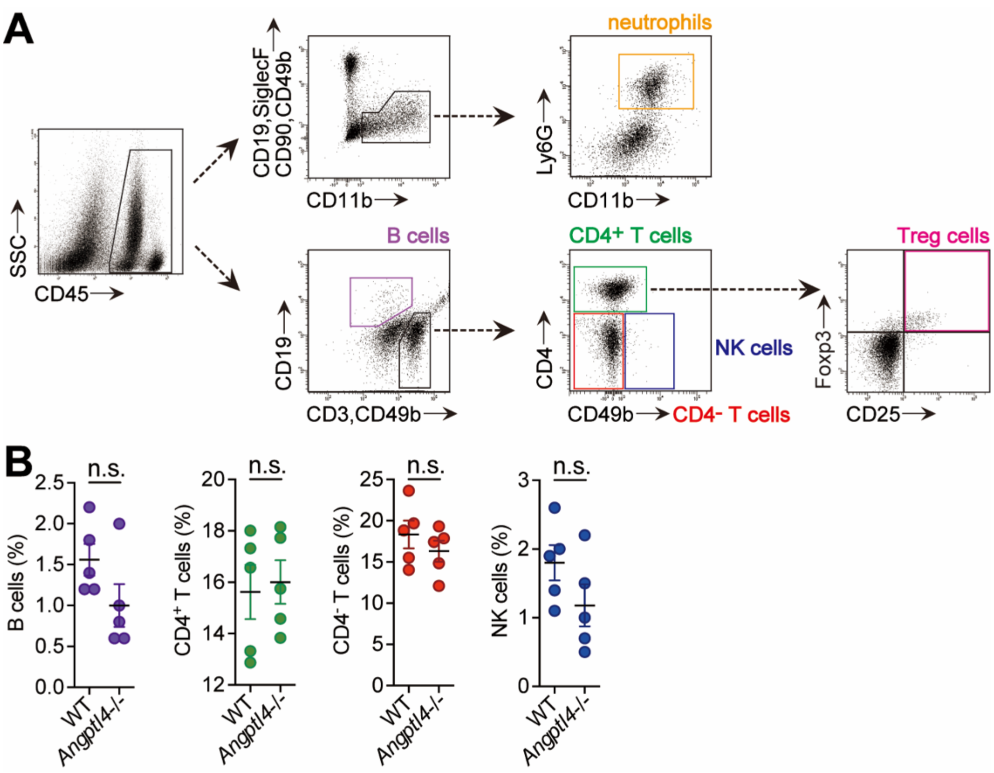

ANGPTL4 increases inflammation in gastric mucosa during *H. pylori* infection. (A) Characterization by flow cytometry of neutrophils, B cells, NK cells, CD4$^+$ T cells, CD4$^-$ T cells and Tregs in the mouse stomachs. (B) The levels of B cells, NK cells, CD4$^+$ T cells and CD4$^-$ T cells in gastric mucosa of WT *H. pylori*-infected WT and *Angptl4*$^{-/-}$ mice at 12 week p.i. were compared (n=5). Results are expressed as the percentage of B cells, NK cells, CD4$^+$ T cells and CD4$^-$ T cells in CD45$^+$ cells. Data are representative of 2 independent experiments. Data are shown as mean ± SEM and analyzed by Student *t* test and Mann-Whitney U test. n.s. P>0.05 for groups connected by horizontal lines.

Fig. S5

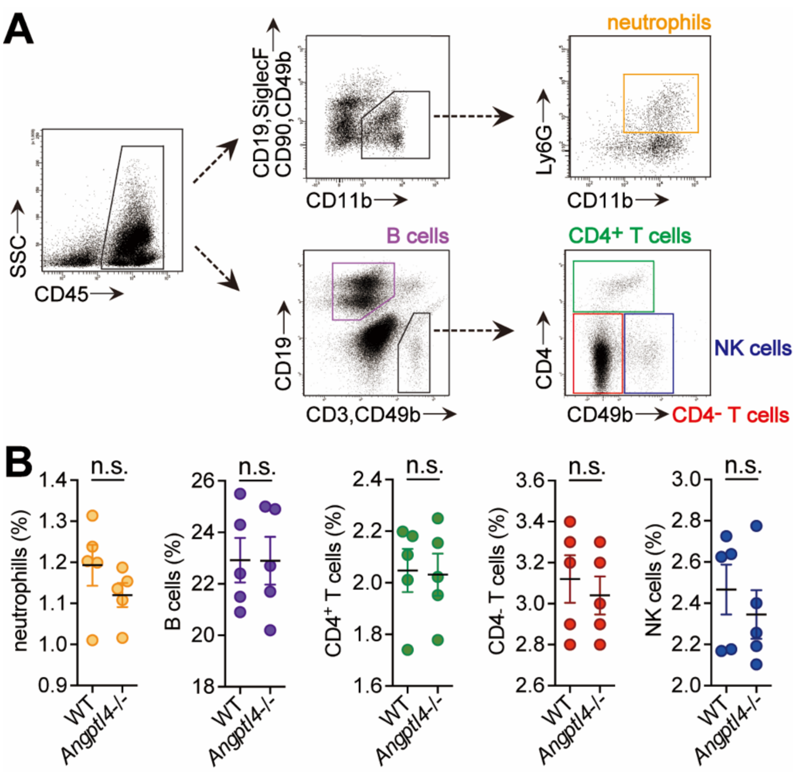

ANGPTL4 has no effect on the accumulation of neutrophils, B cells, NK cells, $CD4^+$ T cells and $CD4^-$ T cells in bone marrow during *H. pylori* infection. (A) Characterization by flow cytometry of neutrophils, B cells, NK cells, $CD4^+$ T cells and $CD4^-$ T cells in the mouse bone marrows. (B) The levels of neutrophils, B cells, NK cells, $CD4^+$ T cells and $CD4^-$ T cells in bone marrow of WT *H. pylori*-infected WT and *Angptl4*$^{-/-}$ mice at 12 week p.i. were compared (n=5). Results are expressed as the percentage of neutrophils, B cells, NK cells, $CD4^+$ T cells and $CD4^-$ T cells in $CD45^+$ cells. Data are representative of 2 independent experiments. Data are shown as mean ± SEM and analyzed by Student *t* test and Mann-Whitney U test. n.s. *P*>0.05 for groups connected by horizontal lines.

Fig. S6

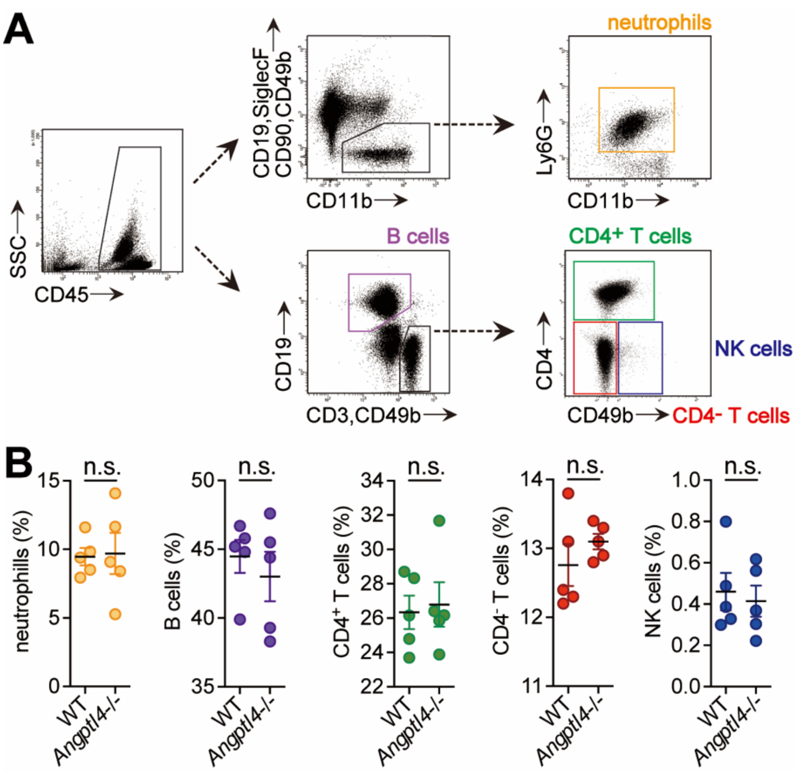

ANGPTL4 has no effect on the accumulation of neutrophils, B cells, NK cells, $CD4^{+}$ T cells and $CD4^{-}$ T cells in blood during *H. pylori* infection. (A) Characterization by flow cytometry of neutrophils, B cells, NK cells, $CD4^{+}$ T cells and $CD4^{-}$ T cells in the mouse bloods. (B) The levels of neutrophils, B cells, NK cells, $CD4^{+}$ T cells and $CD4^{-}$ T cells in blood of WT *H. pylori*-infected WT and *Angptl4*$^{-/-}$ mice at 12 week p.i. were compared (n=5). Results are expressed as the percentage of neutrophils, B cells, NK cells, $CD4^{+}$ T cells and $CD4^{-}$ T cells in $CD45^{+}$ cells. Data are representative of 2 independent experiments. Data are shown as mean ± SEM and analyzed by Student *t* test and Mann-Whitney U test. n.s. *P*>0.05 for groups connected by horizontal lines.

Fig. S7

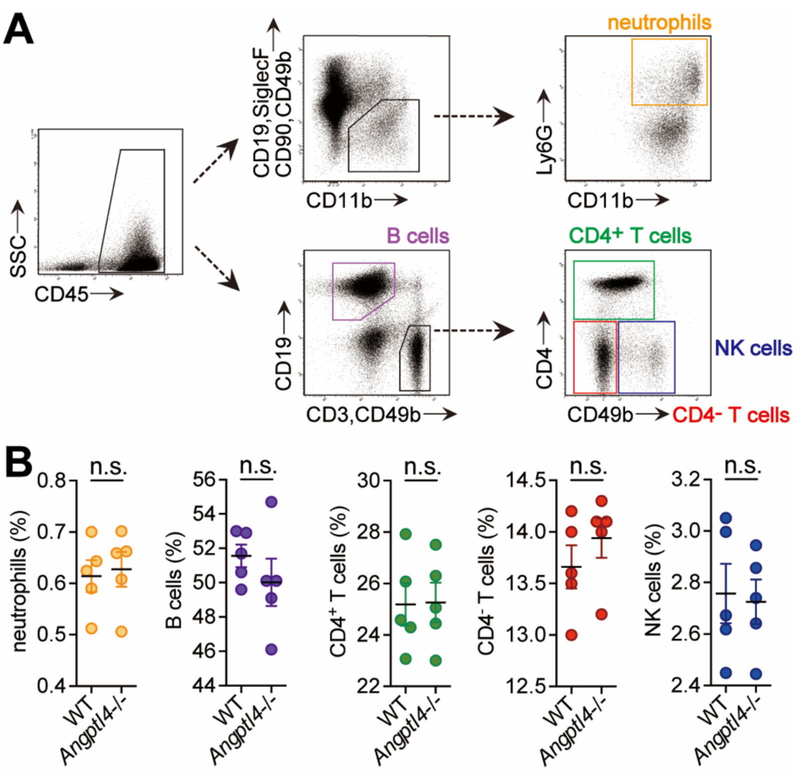

ANGPTL4 has no effect on the accumulation of neutrophils, B cells, NK cells, CD4$^+$ T cells and CD4$^-$ T cells in spleen during *H. pylori* infection. (A) Characterization by flow cytometry of neutrophils, B cells, NK cells, CD4$^+$ T cells and CD4$^-$ T cells in the mouse spleens. (B) The levels of neutrophils, B cells, NK cells, CD4$^+$ T cells and CD4$^-$ T cells in spleen of WT *H. pylori*-infected WT and *Angptl4*$^{-/-}$ mice at 12 week p.i. were compared (n=5). Results are expressed as the percentage of neutrophils, B cells, NK cells, CD4$^+$ T cells and CD4$^-$ T cells in CD45$^+$ cells. Data are representative of 2 independent experiments. Data are shown as mean ± SEM and analyzed by Student *t* test and Mann-Whitney U test. n.s. P>0.05 for groups connected by horizontal lines.

Fig. S8

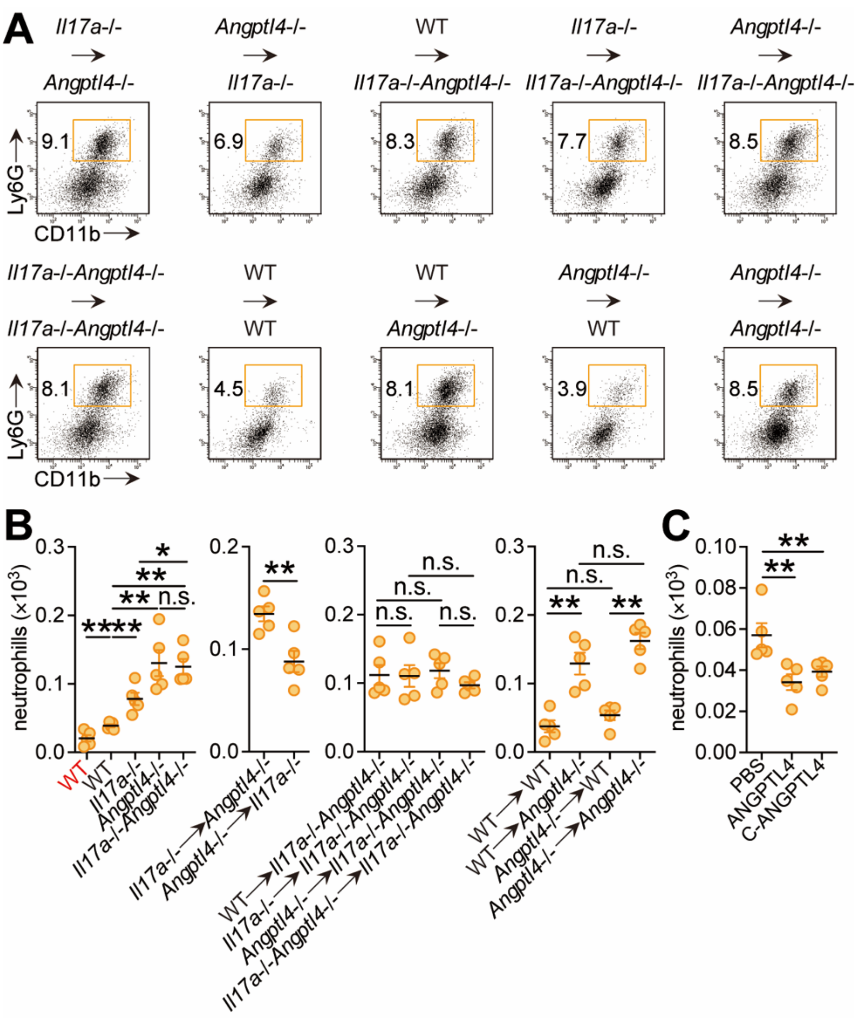

ANGPTL4 decreases neutrophil accumulation in gastric mucosa during *H. pylori* infection. (A) Representative dot plots of neutrophils in gastric mucosa of WT *H. pylori*-infected BM chimera mice at 12 week p.i.. Results are expressed as the percentage of neutrophils in CD45$^+$ cells. (B) The levels of neutrophils in gastric mucosa of uninfected WT mice (red), WT *H. pylori*-infected WT, *Il17a*$^{-/-}$, *Angptl4*$^{-/-}$ and *Il17a*$^{-/-}$*Angptl4*$^{-/-}$ mice, or in gastric mucosa of WT *H. pylori*-infected BM chimera mice at 12 week p.i. were compared (n=5). Results are expressed as the number of neutrophils per million total cells. (C) The levels of neutrophils in gastric mucosa of WT mice injected with ANGPTL4, cANGPTL4 or PBS control at 12 week p.i. were compared (n=5). Results are expressed as the number of neutrophils per million total cells. Data are representative of 2 independent experiments. Data are shown as mean ± SEM and analyzed by Student *t* test, Mann-Whitney U test and 1-way ANOVA. **P*<0.05, ***P*<0.01, n.s. *P*>0.05 for groups connected by horizontal lines.

Fig. S9

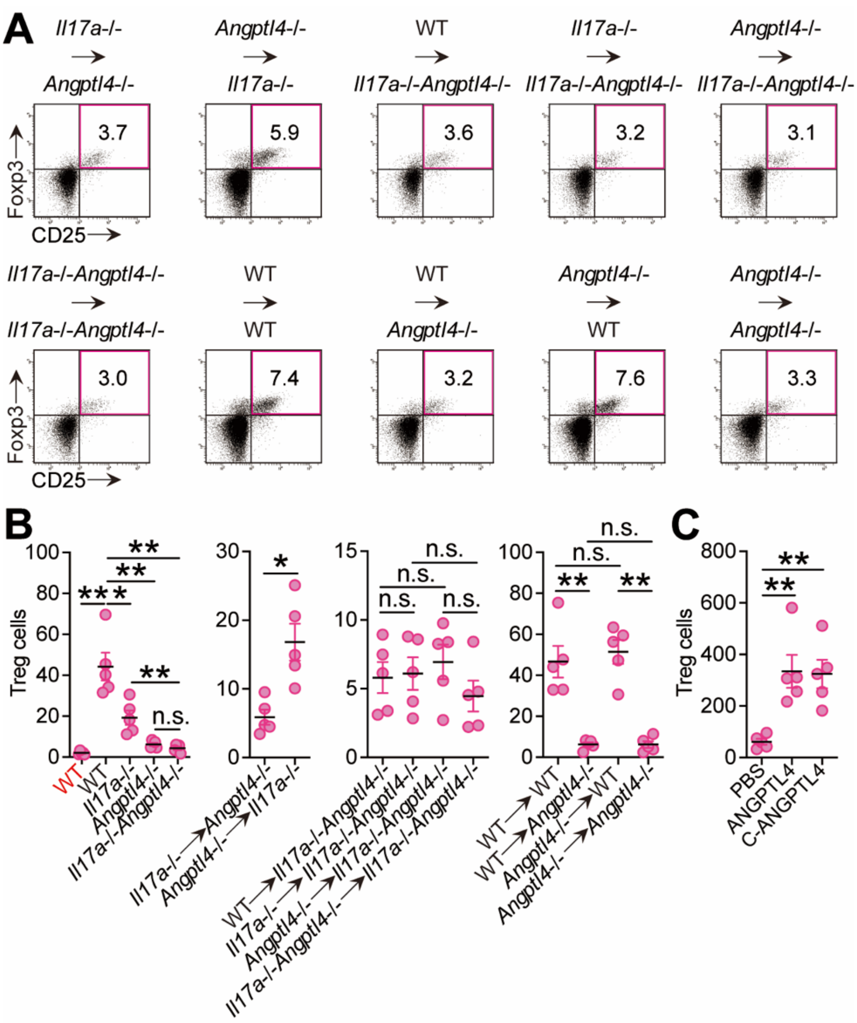

ANGPTL4 increases Treg accumulation in gastric mucosa during *H. pylori* infection. (A) Representative dot plots of Tregs in gastric mucosa of WT *H. pylori*-infected BM chimera mice at 12 week p.i.. Results are expressed as the percentage of Tregs among CD4$^+$ T cells. (B) The levels of Tregs in gastric mucosa of uninfected WT mice (red), WT *H. pylori*-infected WT, $Il17a^{-/-}$, $Angptl4^{-/-}$ and $Il17a^{-/-}Angptl4^{-/-}$ mice, or in gastric mucosa of WT *H. pylori*-infected BM chimera mice at 12 week p.i. were compared (n=5). Results are expressed as the number of Tregs per million total cells. (C) The levels of Tregs in gastric mucosa of WT mice injected with ANGPTL4, cANGPTL4 or PBS control at 12 week p.i. were compared (n=5). Results are expressed as the number of Tregs per million total cells. Data are representative of 2 independent experiments. Data are shown as mean ± SEM and analyzed by Student *t* test, Mann-Whitney U test and 1-way ANOVA. **P*<0.05, ***P*<0.01, n.s. *P*>0.05 for groups connected by horizontal lines.

Fig. S10

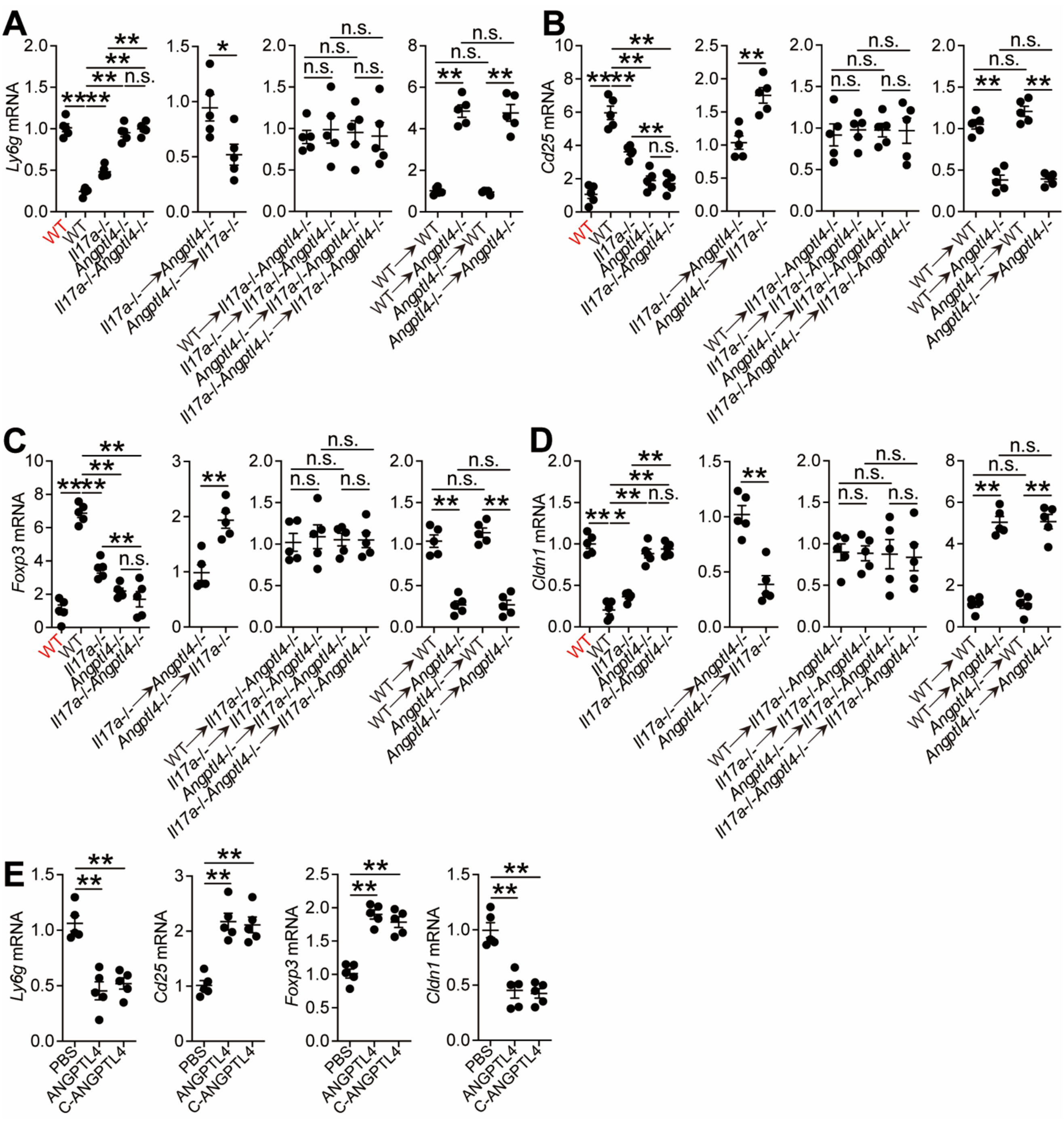

ANGPTL4 increases Treg accumulation, but decreases neutrophil accumulation and CLDN1 expression in gastric mucosa during *H. pylori* infection. (A-D) The expressions of *Ly6g* (A), *Cd25* (B), *Foxp3* (C) and *Cldn1* (D) in gastric mucosa of uninfected WT mice (red), WT *H. pylori*-infected WT, *Il17a*$^{-/-}$, *Angptl4*$^{-/-}$ and *Il17a*$^{-/-}$*Angptl4*$^{-/-}$ mice, or in gastric mucosa of WT *H. pylori*-infected BM chimera mice at 12 week p.i. were compared (n=5). (E) The expressions of *Ly6g*, *Cd25*, *Foxp3* and *Cldn1* in gastric mucosa of WT mice injected with ANGPTL4, cANGPTL4 or PBS control at 12 week p.i. were compared (n=5). Data are representative of 2 independent experiments. Data are shown as mean ± SEM and analyzed by Student *t* test, Mann-Whitney U test and 1-way ANOVA. **P*<0.05, ***P*<0.01, n.s. *P*>0.05 for groups connected by horizontal lines.

Fig. S11

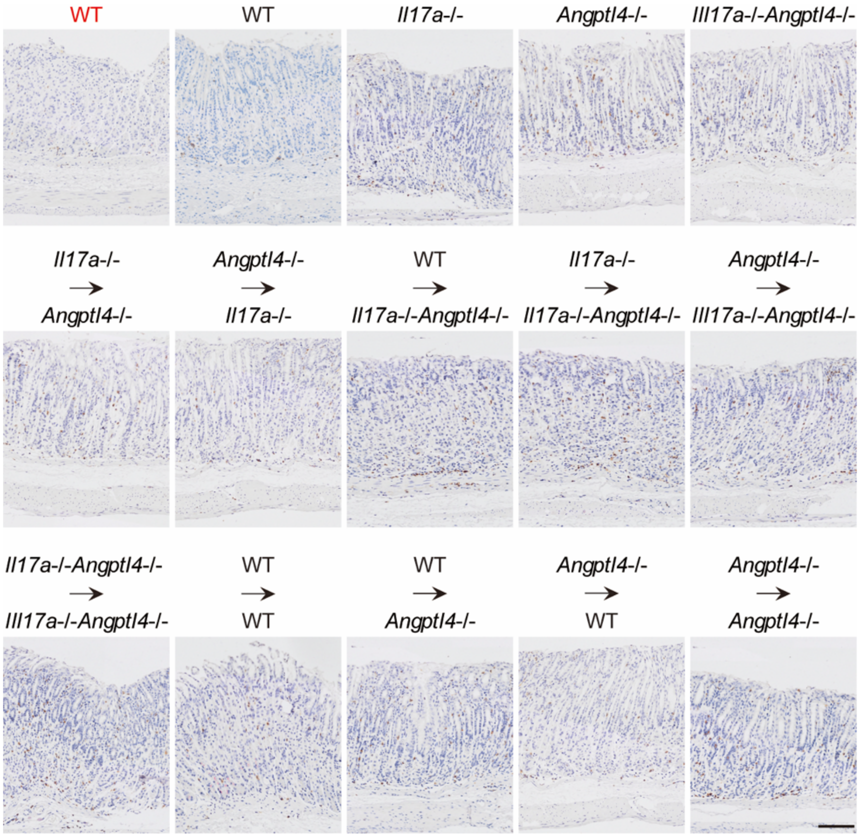

ANGPTL4 decreases neutrophil accumulation in gastric mucosa during *H. pylori* infection. Representative analysis of Ly6G^{+} (brown) neutrophil distributions in gastric mucosa of uninfected WT mice (red), WT *H. pylori*-infected WT, *Il17a*$^{-/-}$, *Angptl4*$^{-/-}$ and *Il17a*$^{-/-}$*Angptl4*$^{-/-}$ mice, or in gastric mucosa of WT *H. pylori*-infected BM chimera mice at 12 week p.i. by immunohistochemical staining. Scale bars: 100 microns.

Fig. S12

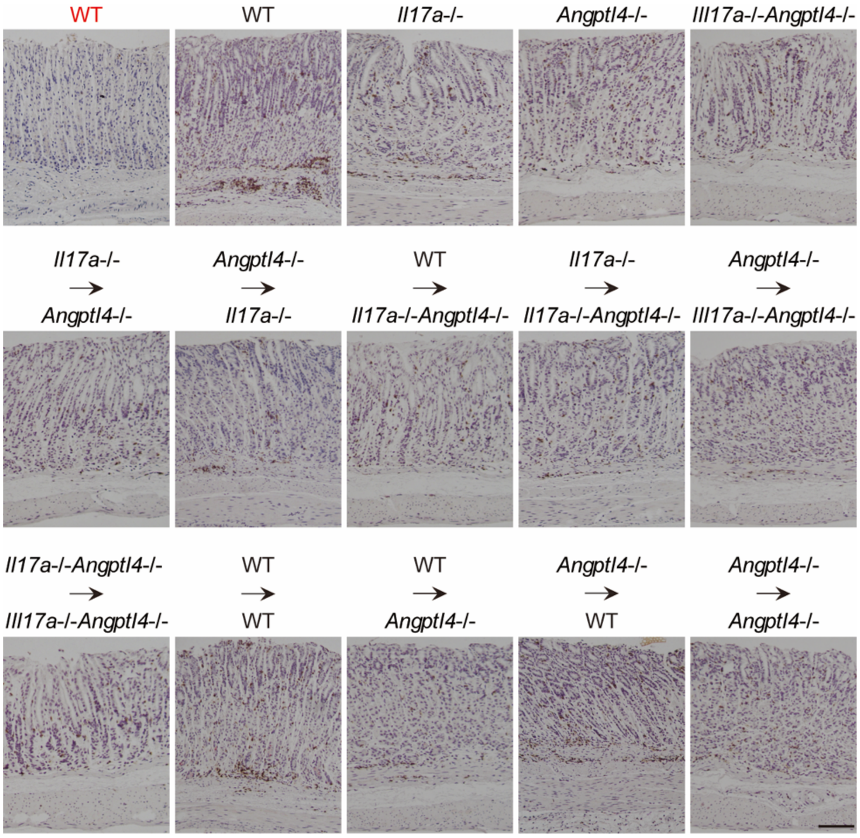

ANGPTL4 increases Treg accumulation in gastric mucosa during *H. pylori* infection. Representative analysis of Foxp3$^+$ (brown) Treg distributions in gastric mucosa of uninfected WT mice (red), WT *H. pylori*-infected WT, *Il17a*$^{-/-}$, *Angptl4*$^{-/-}$ and *Il17a*$^{-/-}$*Angptl4*$^{-/-}$ mice, or in gastric mucosa of WT *H. pylori*-infected BM chimera mice at 12 week p.i. by immunohistochemical staining. Scale bars: 100 microns.

Fig. S13

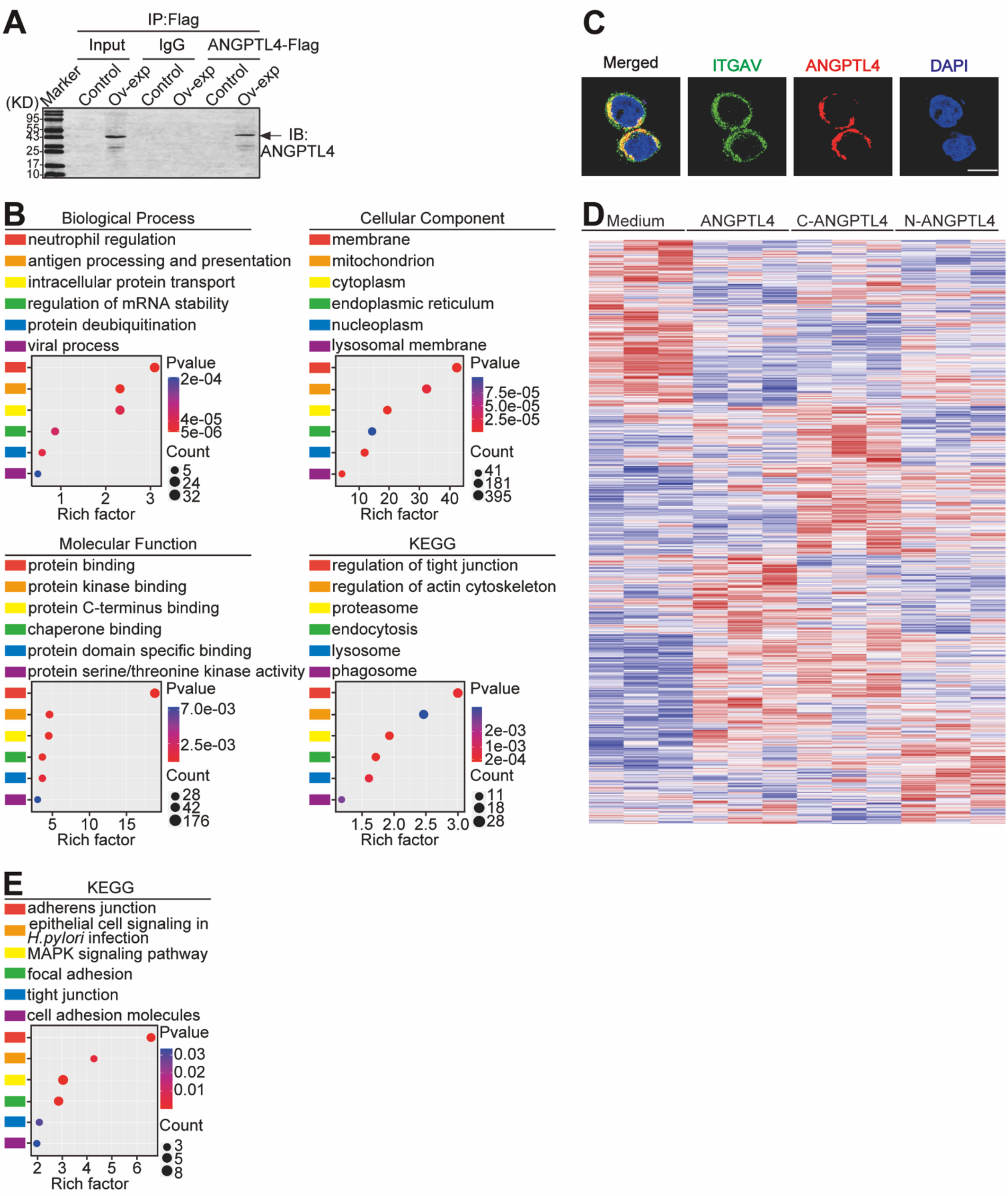

ANGPTL4 binds to ITGAV on GECs to inhibit ERK during *H. pylori* infection. (A) AGS cells expressing the Flag-tagged ANGPTL4 (ANGPTL4-Flag) were lysed and immunoprecipitated (IP) with IgG (control) or anti-Flag Abs. The IP samples were subjected to western blot before mass spectrometry analysis. (B) ANGPTL4-interacting partners were clustered with gene ontology (GO) analysis, and the top 6 GO terms of “Cellular Component”, “Biological Process”, “Molecular Function” and “KEGG” were shown. (C) Confocal microscopy for AGS cells treated with ANGPTL4 (1 μg/ml) at 4°C for 3 h, then fixed and stained with

anti-ANGPTL4 and anti-ITGAV Abs. (D) Heatmap reveals gene changes in AGS cells stimulated with ANGPTL4, cANGPTL4 or nANGPTL4 (1 μg/ml) for 24 h. (E) Compared to unstimulated AGS cells, significantly changed genes in ANGPTL4-stimulated AGS cells were clustered with gene ontology (GO) analysis, and the top 6 GO terms of “KEGG” were shown.

Fig. S14

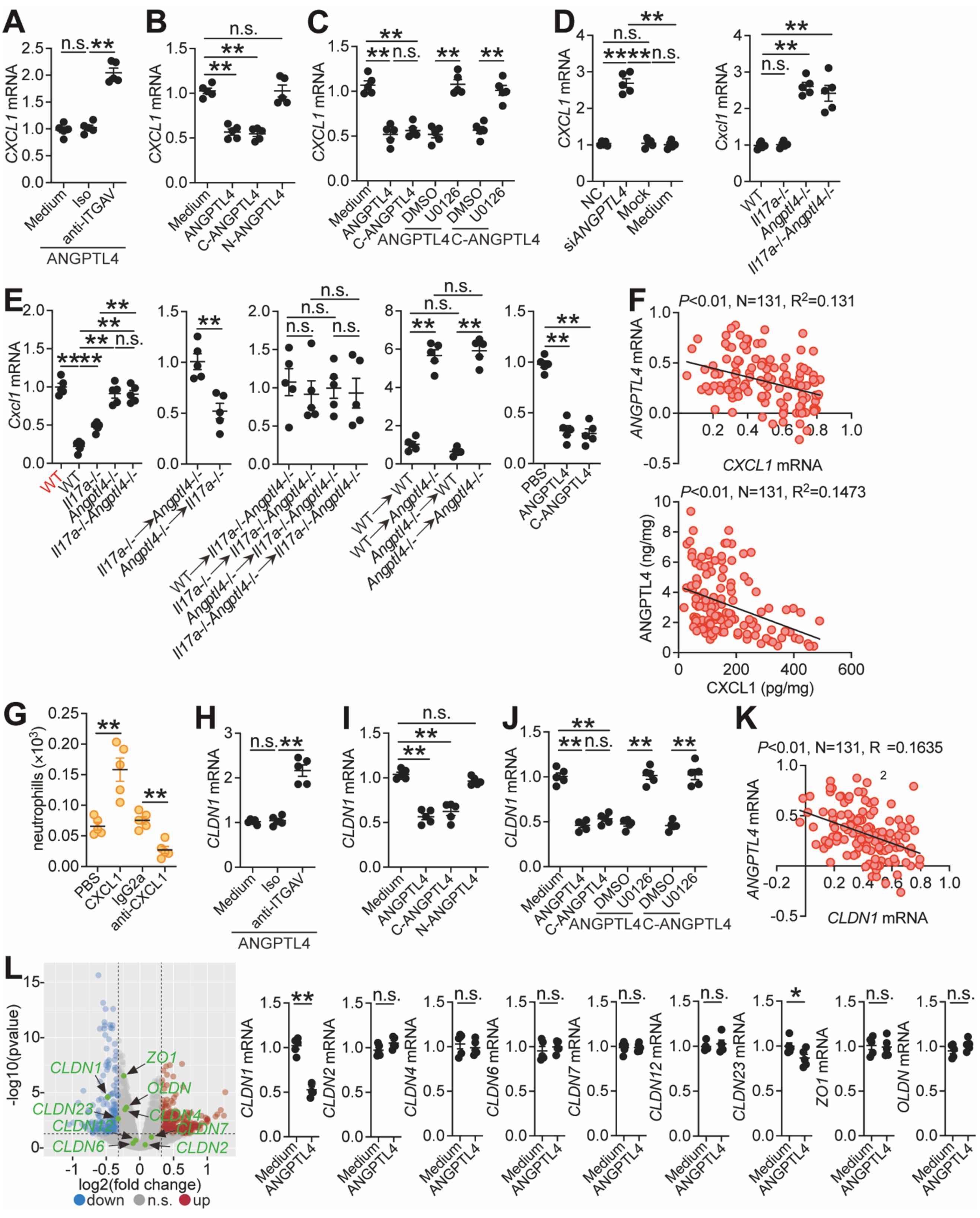

ANGPTL4 suppresses CLDN1 and CXCL1 in GECs through binding to ITGAV to inhibit ERK, leading to decreased neutrophil accumulation and increased bacterial burden during *H. pylori* infection. (A) AGS cells were pre-treated with anti-ITGAV Abs and then stimulated with ANGPTL4 (1 μg/ml) for 24 h. *CXCL1* expression was analyzed by real-time PCR (n=5). (B) AGS cells were stimulated with ANGPTL4, cANGPTL4 or nANGPTL4 (1 μg/ml) for 24 h. *CXCL1* expression was analyzed by real-time PCR (n=5). (C) AGS cells

were pre-treated with U0126 and then stimulated with ANGPTL4 or cANGPTL4 (1 μg/ml) for 24 h. *CXCL1* expression was analyzed by real-time PCR (n=5). (D) *ANGPTL4* siRNA, non-specific control siRNA (NC), or lipo2000 only (Mock) pre-treated AGS cells, and primary GECs from uninfected WT, *Il17a*$^{-/-}$, *Angptl4*$^{-/-}$ and *Il17a*$^{-/-}$*Angptl4*$^{-/-}$ mice were stimulated with WT *H. pylori* (MOI=100) for 24 h. *CXCL1/Cxcl1* expression was analyzed by real-time PCR (n=5). (E) *Cxcl1* expression in gastric mucosa of uninfected WT mice (red), WT *H. pylori*-infected WT, *Il17a*$^{-/-}$, *Angptl4*$^{-/-}$ and *Il17a*$^{-/-}$*Angptl4*$^{-/-}$ mice, in gastric mucosa of WT *H. pylori*-infected BM chimera mice, or in gastric mucosa of WT mice injected with ANGPTL4, cANGPTL4 or PBS control at 12 week p.i. was compared (n=5). (F) The correlations between *ANGPTL4* expression and *CXCL1* expression, and between ANGPTL4 protein and CXCL1 protein in gastric mucosa of *H. pylori*-infected patients were analyzed. Results are expressed as log10(fold change). (G) The neutrophil level in gastric mucosa of WT *H. pylori*-infected mice injected with CXCL1 or PBS control, or anti-CXCL1 Abs or control IgG at 12 week p.i. was compared (n=5). Results are expressed as the number of neutrophils per million total cells. (H) AGS cells were pre-treated with anti-ITGAV Abs and then stimulated with ANGPTL4 (1 μg/ml) for 24 h. *CLDN1* expression was analyzed by real-time PCR (n=5). (I) AGS cells were stimulated with ANGPTL4, cANGPTL4 or nANGPTL4 (1 μg/ml) for 24 h. *CLDN1* expression was analyzed by real-time PCR (n=5). (J) AGS cells were pre-treated with U0126 and then stimulated with ANGPTL4 or cANGPTL4 (1 μg/ml) for 24 h. *CLDN1* expression was analyzed by real-time PCR (n=5). (K) The correlation between *ANGPTL4* expression and *CLDN1* expression in gastric mucosa of *H. pylori*-infected patients was analyzed. Results are expressed as log10(fold change). (L) VolcanoPlot reveals gene changes of tight junction molecules in AGS cells stimulated with ANGPTL4 (1 μg/ml) for 24 h. AGS cells were stimulated with ANGPTL4 (1 μg/ml) for 24 h. The expressions of tight junction molecules (*CLDN1*, *CLDN2*, *CLDN4*, *CLDN6*, *CLDN7*, *CLDN12*, *CLDN23*, *ZO1*, *OLDN1*) were analyzed by real-time PCR (n=5). Data are representative of 2 independent experiments. Data are shown as mean ± SEM and analyzed by Student *t* test, Mann-Whitney U test and 1-way ANOVA. *P<0.05, **P<0.01, n.s. P> 0.05 for groups connected by horizontal lines.

Fig. S15

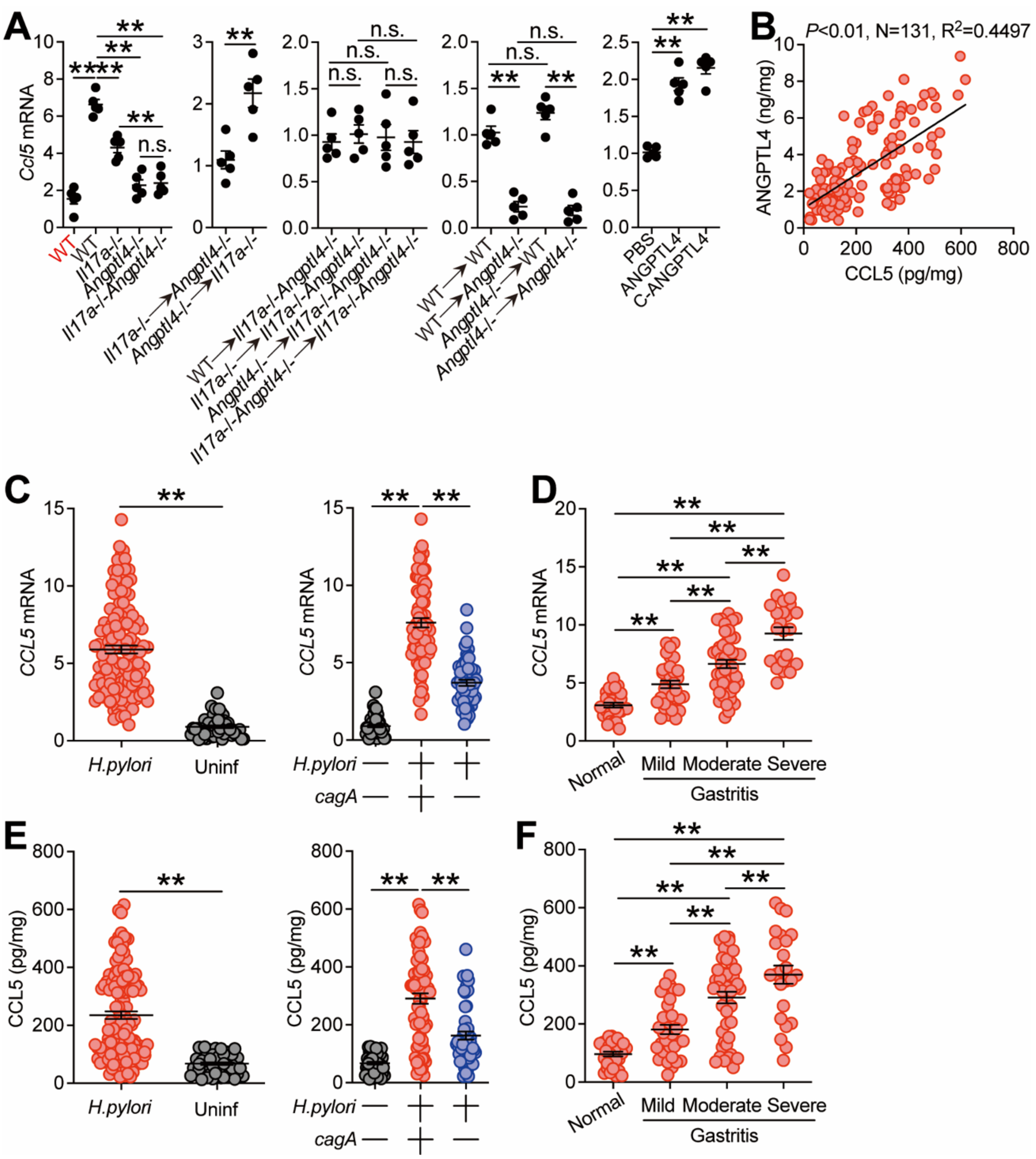

ANGPTL4 promotes CCL5 in monocytes through binding to ITGAV to activate NF-κB during *H. pylori* infection. (A) *Ccl5* expression in gastric mucosa of uninfected WT mice (red), WT *H. pylori*-infected WT, *Il17a*$^{-/-}$, *Angptl4*$^{-/-}$ and *Il17a*$^{-/-}$*Angptl4*$^{-/-}$ mice, in gastric mucosa of WT *H. pylori*-infected BM chimera mice, or in gastric mucosa of WT mice injected with ANGPTL4, cANGPTL4 or PBS control at 12 week p.i. was compared (n=5). (B) The correlation between ANGPTL4 protein and CCL5 protein in gastric mucosa of *H. pylori*-infected patients was analyzed. (C and E) *CCL5* expression (C) or CCL5 protein (E) in gastric mucosa of *H. pylori*-infected (n=131) and uninfected donors (n=50), or in gastric mucosa of *cagA*$^{+}$ *H. pylori*-infected (n=74), *cagA*$^{-}$ *H. pylori*-infected (n=57), and uninfected donors (n=50) was compared. (D and F) *CCL5* expression (D) or CCL5 protein (F) in gastric mucosa of *H. pylori*-infected patients with mild (n=34), moderate (n=45), severe inflammation (n=24), and with normal gastric histopathology (n=28) was compared. Data are representative of 2 independent

experiments. Data are shown as mean ± SEM and analyzed by Student *t* test, Mann-Whitney U test and 1-way ANOVA. *$P<0.05$, **$P<0.01$, n.s. $P>0.05$ for groups connected by horizontal lines.

Fig. S16

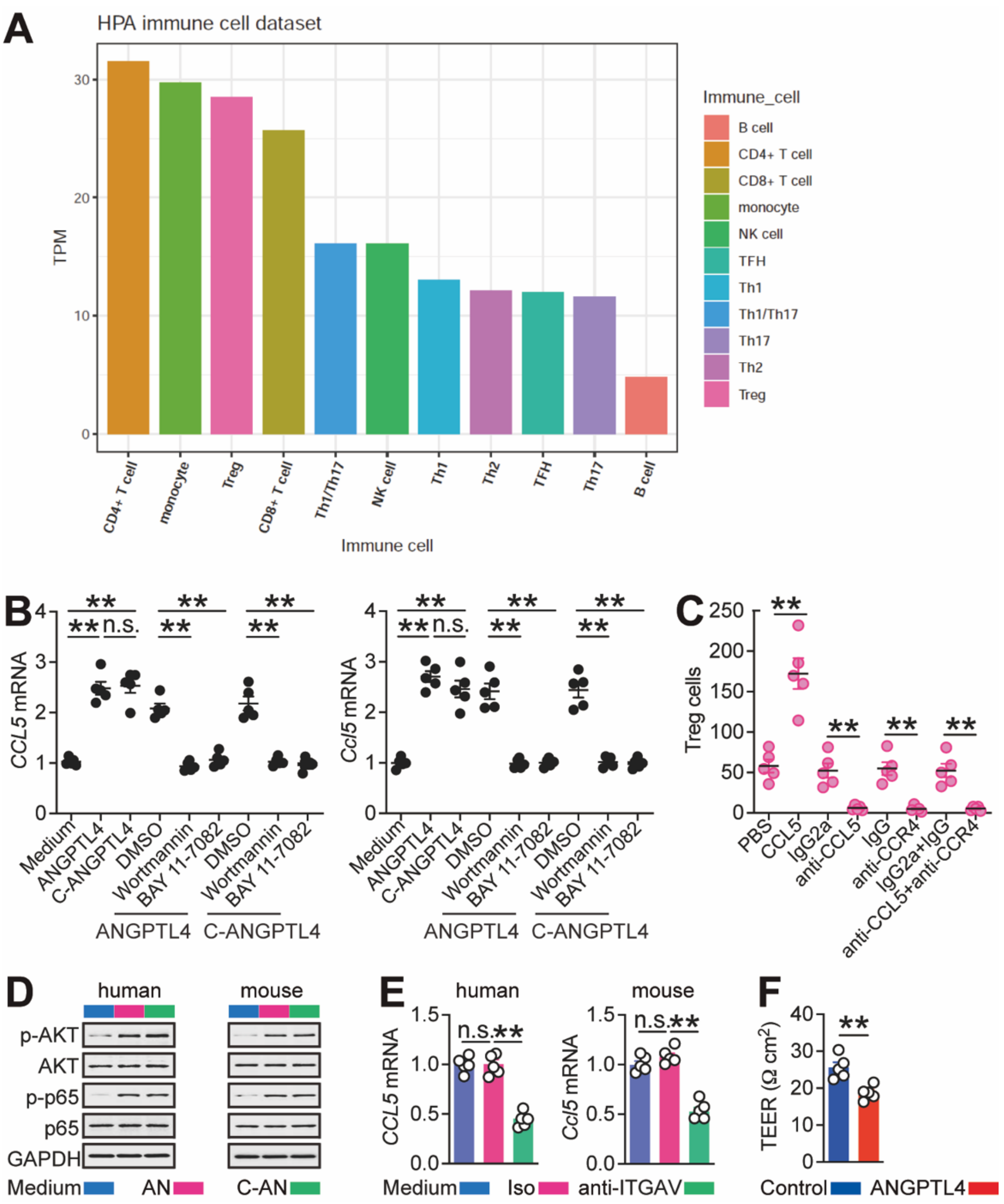

ANGPTL4 promotes CCL5 in monocytes through binding to ITGAV to activate NF-κB, leading to increased Treg accumulation during *H. pylori* infection. (A) ITGAV expression on immune cells in HPA immune cell dataset (https://www.proteinatlas.org/ENSG00000138448-ITGAV/immune+cell). (B) Human (left panel) or mouse (right panel) monocytes were pre-treated with Wortmannin or BAY 11-7082 and then stimulated with ANGPTL4 or cANGPTL4 (1 μg/ml) for 24 h. *CCL5*/*Ccl5* expression was analyzed by real-time PCR (n=5). (C) The Treg level in gastric mucosa of WT *H. pylori*-infected mice injected with CCL5 or PBS control, and/or anti-CCR4 Abs or control IgG at 12 week p.i. was compared (n=5). Results are expressed as the number of Tregs per million total cells. (D) Monocytes were stimulated with ANGPTL4 or cANGPTL4 (1 μg/ml) for 6 h. AKT and p-AKT, p65 and p-p65 proteins were analyzed by western blot. (E) Monocytes were pre-treated with anti-ITGAV Abs and then stimulated with ANGPTL4 (1 μg/ml) for 24 h. *CCL5*/*Ccl5* expression was analyzed by real-time PCR (n=5). (F) AGS cells were stimulated with ANGPTL4 (1 μg/ml) for 24 h. Transepithelial

electrical resistance (TEER) measurements were then performed, and TEER values were further calculated. Data are representative of 2 independent experiments. Data are shown as mean ± SEM and analyzed by Student *t* test, Mann-Whitney U test and 1-way ANOVA. Western blot results are run in parallel and contemporaneously. *$P<0.05$, **$P<0.01$, n.s. $P> 0.05$ for groups connected by horizontal lines.

Fig. S17

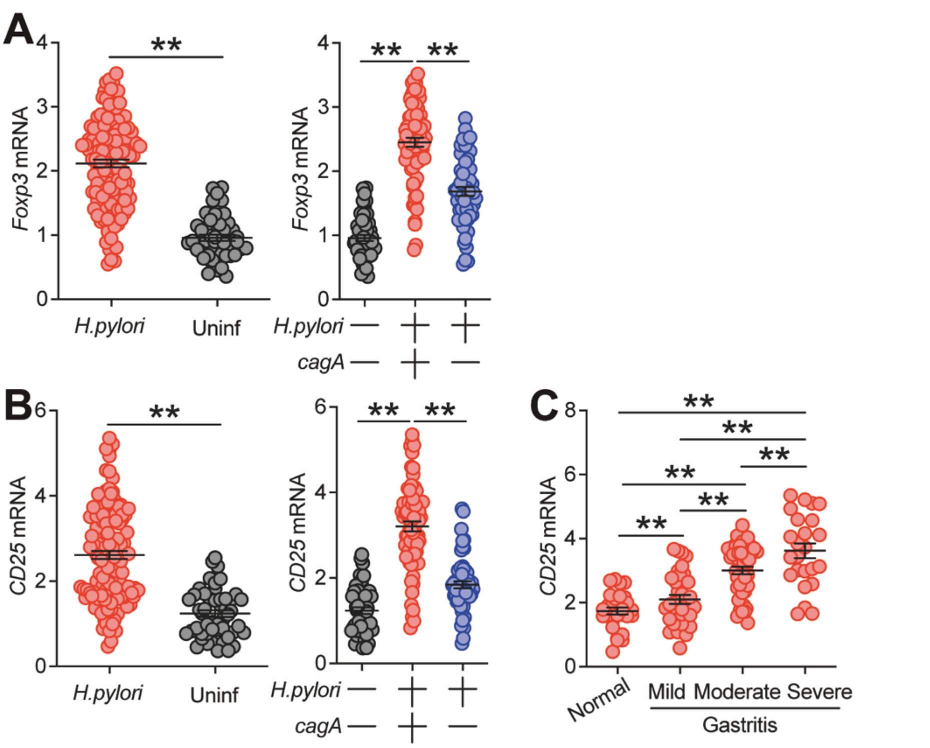

ANGPTL4 promotes Treg proliferation, aggravating inflammation during *H. pylori* infection. (A and B) *Foxp3* expression (A) or *CD25* expression (B) in gastric mucosa of *H. pylori*-infected (n=131) and uninfected donors (n=50), or in gastric mucosa of *cagA*$^{+}$ *H. pylori*-infected (n=74), *cagA*$^{-}$ *H. pylori*-infected (n=57), and uninfected donors (n=50) was compared. (C) *CD25* expression in gastric mucosa of *H. pylori*-infected patients with mild (n=34), moderate (n=45), severe inflammation (n=24), and with normal gastric histopathology (n=28) was compared. Data are shown as mean ± SEM and analyzed by Student *t* test, Mann-Whitney U test and 1-way ANOVA. *P<0.05, **P<0.01, n.s. P> 0.05 for groups connected by horizontal lines.
