# Supplementary material for: Helicobacter pylori-Induced Angiopoietin-Like 4 Promotes Gastric Bacterial Colonization and Gastritis
Source: Research (Wash D C). 2024 Jul 17;7:0409. doi: 10.34133/research.0409 (PMC11254415; doi:10.34133/research.0409)
Supplement: Supplementary 1 — Figs. S1 to S17 Tables S1 to S8 [file research.0409.f1.zip › revised Supplementary Tables(R1).docx]

Table. S1. Clinical characteristics of patients

| Variables | *H. pylori*-infected | Uninfected | Gastric ulcer |
| --- | --- | --- | --- |
| Age (median, range) | (50 year, 16–86 years) | (51 year, 23–71 years) | (45 year, 28–66 years) |
| Sex (male/female) | 72/59 | 31/19 | 20/12 |

Exclusion criteria were: previous treatment for *H. pylori* infection, use of inhibitors of acid secretion and/or antibiotics during the 2 months before the study, use of anticoagulant drugs in the last week, gastrointestinal malignancy, severe concomitant cardiovascular, respiratory or endocrine diseases, clinically significant renal or hepatic disease, haematological disorders, previous gastro-oesophageal surgery, history of allergy to any of the drug used in the study, pregnancy or lactation, alcohol abuse, drug addiction, severe neurological or psychiatric disorders, and long-term use of corticosteroids or anti-inflammatory drugs.

Table. S2. Antibodies and other reagents

| Antibodies and reagents | Manufacturers |
| --- | --- |
| Antibodies for flow cytometry |  |
| Part1 |  |
| anti-mouse CD45-BV421 (563890) | BD Pharmingen |
| anti-mouse CD11b-PE-Cy7 (552850) | BD Pharmingen |
| anti-mouse Siglec-F-PE (562068) | BD Pharmingen |
| anti-mouse CD19-PE (561736) | BD Pharmingen |
| anti-mouse CD90-PE (553005) | BD Pharmingen |
| anti-mouse CD49b-PE (108907) | Biolegend |
| anti-mouse CD11b-APC-Cy7 (101226) | Biolegend |
| anti-mouse Ly6G-FITC (127606) | Biolegend |
| Part2 |  |
| anti-mouse CD19-APC (152410) | Biolegend |
| anti-mouse CD3-BV605 (100237) | Biolegend |
| anti-mouse CD49b-BV605 (740363) | BD Bioscience |
| anti-mouse CD4-PE (100408) | Biolegend |
| anti-mouse CD49b-PerCP-Cy5.5 (103620) | Biolegend |
| anti-mouse CD45-BV421(103133) | Biolegend |
| Part3 |  |
| anti-mouse CD19-PE-Cy7 (152418)  anti-mouse CD4-FITC (100405) | Biolegend  Biolegend |
| anti-mouse CCR4-PerCP-Cy5.5 (131219) | Biolegend |
| anti-mouse CD25-PE (102007) | Biolegend |
| anti-mouse Foxp3-Alexa Fluor® 647 (320013) | Biolegend |
| anti-mouse CD3-APC/Fire^TM^ 750 (100247)  anti-mouse CD49b-APC/Fire^TM^ 750 (108926) | Biolegend  Biolegend |
| anti-mouse CD45-BV421(103133) | Biolegend |
| Part4 |  |
| anti-human CD45-PE-Cy7 (304016) | Biolegend |
| anti-human CD11b-PerCP-Cy5.5 (301328) | Biolegend |
| anti-human CD66b-FITC (305104) | Biolegend |
| anti-human CD15-APC-Cy7 (323048) | Biolegend |
| Part5 |  |
| anti-human CD45-PE-Cy7 (304016) | Biolegend |
| anti-human CD19-BV421 (302234) | Biolegend |
| anti-human CD56-FITC (362546) | Biolegend |
| anti-human CD3-APC-Cy7 (300426) | Biolegend |
| anti-human CD4-BV605 (344646) | Biolegend |
| anti-human CD25-PE (356104) | Biolegend |
| anti-human Foxp3-Alexa Fluor® 647 (320214) | Biolegend |
| anti-human CCR4-PerCP-Cy5.5 (359406)  Part6  anti-mouse CD45-BV421(103133)  anti-mouse CD3-APC/Fire^TM^ 750 (100247)  anti-mouse CD4-FITC (100405)  anti-mouse IL-17A-APC (506915) | Biolegend  Biolegend  Biolegend  Biolegend  Biolegend |
| Antibodies for immunohistochemical staining |  |
| rabbit anti-human/mouse ANGPTL4 (40-9800, 1:100) | Invitrogen |
| rabbit anti-mouse Ly6G (ab238132, 1:200)  rabbit anti-mouse Foxp3 (ab255942, 1:100) | Abcam  Abcam |
| horseradish peroxidase anti-rabbit IgG (ZB-2301) | Zhongshan Biotechnology |
| horseradish peroxidase anti-mouse IgG (ZB-2305) | Zhongshan Biotechnology |
| Antibodies for immunofluorescence |  |
| rabbit anti-human/mouse ANGPTL4 (ab196746,1:100)  rabbit anti-human/mouse pepsinogen II/PGC (ab255826,1:1000)  rabbit anti-human Epcam (MA5-12436, 1:100)  mouse anti-human ITGAV (327902,1:100)  mouse anti-human/mouse Claudin 1 (37-4900,1:200)  rabbit anti-human/mouse CD14 (MA5-35200,1:200)  rabbit anti-human/mouse CCL5 (710001,1:100) | Abcam  Abcam  Invitrogen  Biolegend  Invitrogen  Invitrogen  Invitrogen |
| goat anti-rabbit secondary antibody, AF Plus 555 (A32732,1:1000)  goat anti-rabbit secondary antibody, AF Plus 488 (A32731,1:1000) | Invitrogen  Invitrogen |
| goat anti-mouse secondary antibody, AF Plus 488 (A32723,1:1000) | Invitrogen |
| Antibodies for neutralizing and blocking |  |
| anti-human ITGAV (Mouse IgG2a) (327902)  anti-mouse ITGAV (Rat IgG1) (104102) | Biolegend  Biolegend |
| anti-human CCL5 (Mouse IgG1) (MAB278)  anti-mouse CCL5 (Rat IgG2a) (MAB478)  anti-human/mouse CCR4 (Sheep IgG) (AF5207) | R&D Systems  R&D Systems  R&D Systems |
| anti-human CXCL1 (Mouse IgG2b) (MAB275)  anti-mouse CXCL1 (Rat IgG2a) (MAB4531)  Mouse IgG2a Isotype Control (400201)  Rat IgG1 Isotype Control (400401)  Mouse IgG1 Isotype Control (MAB002) | R&D Systems  R&D Systems  R&D Systems  Biolegend  Biolegend |
| Rat IgG2a Isotype Control (MAB006) | R&D Systems |
| Sheep IgG Control (5-001-A)  Mouse IgG2b Isotype Control (MAB004)  anti-mouse Ly6G (Rat IgG2a)(127650)  anti-mouse CD25 (Rat IgG1)(102059)  Rat IgG2a Isotype Control (400566)  Rat IgG1 Isotype Control (401916)  anti-human IL-17A (Mouse IgG2b) (MAB317-100)  anti-human IL-17RA (Mouse IgG1) (MAB177-100) | R&D Systems  R&D Systems  Biolegend  Biolegend  Biolegend  Biolegend  R&D Systems  R&D Systems |
| Antibodies for western blot |  |
| rabbit anti-human/mouse ANGPTL4 (40-9800, 1:1000)  mouse anti-human/mouse Claudin 1 (37-4900, 1:1000) | Invitrogen  Invitrogen |
| rabbit anti-human/mouse NF-κBp65 (8242,1:1000) | Cell Signaling Technology |
| rabbit anti-human/mouse p-NF-κBp65 (Ser536) (3033, 1:1000) | Cell Signaling Technology |
| rabbit anti-human/mouse p-AKT (Thr308) (13038, 1:1000) | Cell Signaling Technology |
| rabbit anti-human/mouse AKT (4685, 1:1000) | Cell Signaling Technology |
| rabbit anti-human/mouse GAPDH (5174, 1:1000)  mouse anti-human ITGAV (327902, 1:100) | Cell Signaling Technology  Biolegend |
| Antibodies for ChIP |  |
| rabbit anti-human NF-κBp65 (8242S,1:100) | Cell Signaling Technology |
| rabbit IgG Isotype Control (ab172730) | Abcam |
| Antibodies for co-immunoprecipitation |  |
| mouse anti-Flag (8146S, 1:50) | Cell Signaling Technology |
| mouse IgG Isotype Control (5415S, 1:50) | Cell Signaling Technology |
| ELISA kits |  |
| human ANGPTL4 | Thermo Fisher Scientific |
| human CCL5 | Thermo Fisher Scientific |
| human CXCL1 | Thermo Fisher Scientific |
| mouse ANGPTL4 | Thermo Fisher Scientific |
| mouse CCL5 | Thermo Fisher Scientific |
| mouse CXCL1 | Thermo Fisher Scientific |
| human IL-17A | Thermo Fisher Scientific |
| Reagents for mouse gastric organoid cultures |  |
| Murine Wnt3a (315-20) | PeproTech |
| Murine Noggin (250-38) | PeproTech |
| Murine R-Spindin-1 (315-32) | PeproTech |
| Murine EGF (315-09) | PeproTech |
| Murine FGF-10 (450-61) | PeproTech |
| Murine Gastrin I (1003377) | PeproTech |
| N-2 Supplement (17502048) | Thermo Fisher Scientific |
| Advanced DMEM/F12 (12634010) | Thermo Fisher Scientific |
| GlutaMAX Supplement (35050061) | GIBCO |
| Y-27632 dihydrochloride (Y0503) | Sigma-Aldrich |
| Matrigel® Basement Membrane Matrix (356234) | Corning |
| Reagents for human gastric organoid cultures |  |
| Human Gastric Epithelial Organoid Kit | Biogenous |
| Organoid Recovery Solution | Biogenous |
| Anti-Adherence Rinsing Solution | Biogenous |
| Organoid Dissociation Solution | Biogenous |
| Organoid Cryopreservation Medium (Serum Free) | Biogenous |
| Advanced DMEM/F-12 (12634010) | Thermo Fisher Scientific |
| Matrigel® Basement Membrane Matrix (356234) | Corning |
| DPBS | Solarbio |
| Reagents for signaling pathway inhibition |  |
| IκBα inhibitor BAY 11-7082 | Merck Millipore |
| MEK-1 and MEK-2 inhibitor U0126 | Merck Millipore |
| STAT3 inhibitor FLLL32 | Merck Millipore |
| JNK inhibitor SP600125 | Merck Millipore |
| JAK inhibitor AG490 | Merck Millipore |
| MAPKp38 inhibitor SB203580 | Merck Millipore |
| PI3K inhibitor Wortmannin  MEK-1 and MEK-2 inhibitor U0126 | Merck Millipore  Merck Millipore |
| *cagA* EPIYA motif phosphorylation inhibitor PP2 | Merck Millipore |
| Reagents for luciferase reporter assay and ChIP |  |
| Dual-Luciferase Reporter assay Kit | Promega |
| Endo-free Plasmid Mini Kit | Omega |
| Pierce Magnetic ChIP Kit | Thermo Fisher Scientifific |
| truChIP Chromatin Shearing Kit | Covaris |
| 16% Formaldehyde, Methanol-Free | Cell Signaling Technology |
| Protease inhibitor | Thermo Fisher Scientific |
| Human CD326 microbeads | MiltenyiBiotec |
| Mouse CD326 microbeads  Purified anti-human CD3 antibodies  Purified anti-human CD28 antibodies  Purified anti-mouse CD3 antibodies  Purified anti-mouse CD28 antibodies  DAB kit  EnVision™ G2 System/AP Rabbit/Mouse (Permanent Red) | MiltenyiBiotec  Biolegend  Biolegend  Biolegend  Biolegend  Zhongshan Biotechnology  Dako |
| EasySep™ Mouse Monocyte Isolation Kit | StemCell Technologies |
| EasySep™ Human CD14 Positive Selection Kit | StemCell Technologies |
| Leukocyte Activation Cocktail, with BD GolgiPlug™ | BD Pharmingen |
| Perm/Wash solution  Foxp3 Perm/Wash solution  8-μm pore size Transwells | BD Pharmingen  eBioscience  Corning |
| 5-μm pore size Transwells  3-μm pore size Transwells  0.4-μm pore size Transwells | Corning  Corning  Corning |
| Collagenase Ι | Sigma-Aldrich |
| Collagenase IV | Sigma-Aldrich |
| DNase I | Sigma-Aldrich |
| DMSO | Beyotime |
| Complete, EDTA free, EASYpack | Roche |
| PhosStop EASYpack | Roche |
| Super ECL Plus Western Blotting Substrate | Bioground |
| Fetal bovine serum (FBS) | PAN |
| Penicillin/Streptomycin | Beyotime |
| Ampicillin, Sodium Salt | Solarbio |
| RPMI-1640 | Hyclone |
| DMEM/F12 (1:1) | Hyclone |
| Ficoll-Paque Plus | GE Healthcare |
| Red Cell Lysis Buffer | TIANGEN |
| BD FACS™ Lysing Solution | BD Pharmingen |
| TRIzol reagent | TaKaRa |
| Lipofectamine™ 2000 Transfection Reagent | Invitrogen |
| QIAamp DNA Mini Kit | QIAGEN |
| PrimeScript™ RT reagent Kit (Perfect Real Time) | TaKaRa |
| TB Green® Premix Ex Taq™ II | TaKaRa |
| Premix Ex Taq™ (Probe qPCR) | TaKaRa |
| Recombinant human flANGPTL4 | R&D Systems |
| Recombinant human cANGPTL4 | R&D Systems |
| Recombinant human nANGPTL4 | R&D Systems |
| Recombinant mouse flANGPTL4 | R&D Systems |
| Recombinant mouse cANGPTL4 | R&D Systems |
| All other recombinant human/mouse cytokines and chemokines | PeproTech |

APC-Cy7, allophycocyanin-cyanin 7; PE-Cy7, phycoerythrin-cyanin 7; FITC, fluorescein isothiocyanate; PE, phycoerythrin; PerCP-Cy5.5, peridinchlorophyl protein-cyanin 5.5; APC, allophycocyanin; IL, interleukin.

Table. S3. Primer and probe sequences for real-time PCR analysis

| Gene | Primer or probe | Sequence 5′→3′ |
| --- | --- | --- |
| *H. pylori* 16s rDNA | forward | TTTGTTAGAGAAGATAATGACGGTATCTAAC |
|  | reverse | CATAGGATTTCACACCTGACTGACTATC |
|  | probe | CGTGCCAGCAGCCGCGGT |
| Mouse *β2-microglobulin* | forward | CCTGCAGAGTTAAGCATGCCAG |
|  | reverse | TGCTTGATCACATGTCTCGATCC |
|  | probe | TGGCCGAGCCCAAGACCGTCTAC |
| *H. pylori cagA* | forward | GAGTCATAATGGCATAGAACCTGAA |
|  | reverse | TTGTGCAAGAAATTCCATGAAA |
| Mouse *Sry* | forward | TGGGACTGGTGACAATTGTC |
|  | reverse | GAGTACAGGTGTGCAGCTCT |
| Mouse *β-actin* | forward | AGTGTGACGTTGACATCCGT |
|  | reverse | GCAGCTCAGTAACAGTCCGC |
| Mouse *Angptl4* | forward | AAAAGATGCACCCTTCAAAGAC |
|  | reverse | AAAAGATGCACCCTTCAAAGAC |
| Mouse *Ccl1* | forward | CTTCCCCTGAAGTTTATCCAGT |
|  | reverse | TCTACCTTTGTTCAGCCTGAAT |
| Mouse *Ccl2* | forward | TCACCTGCTGCTACTCATTCA |
|  | reverse | CACTGTCACACTGGTCACTCC |
| Mouse *Ccl3* | forward | TTCTCTGTACCATGACACTCTGC |
|  | reverse | CGTGGAATCTTCCGGCTGTAG |
| Mouse *Ccl4* | forward | TGTCTGCCCTCTCTCTCCTCT |
|  | reverse | AGCAAGGACGCTTCTCAGTGA |
| Mouse *Ccl5* | forward | GCTGCTTTGCCTACCTCTCC |
|  | reverse | TCGAGTGACAAACACGACTGC |
| Mouse *Ccl6* | forward | CCAAGACTGCCATTTCATTC |
|  | reverse | AAGCAATGACCTTGTTCCCA |
| Mouse *Ccl7* | forward | ATGGAAGTCTGCGCTGAAG |
|  | reverse | ACATGAGGTCTCCAGAGCTTT |
| Mouse *Ccl8* | forward | ACGCTAGCCTTCACTCCAAAA |
|  | reverse | TTCCAGCTTTGGCTGTCTCTT |
| Mouse *Ccl9* | forward | TGGCATATCTGGCTTTGTCA |
|  | reverse | ATGGCTGTAGCTCAAGATGGT |
| Mouse *Ccl11* | forward | TCCACAGCGCTTCTATTCCT |
|  | reverse | GCAGTTCTTAGGCTCTGGGTT |
| Mouse *Ccl12* | forward | TCGAAGTCTTTGACCTCAACA |
|  | reverse | GGGAACTTCAGGGGGAAATA |
| Mouse *Ccl19* | forward | ACTTGCACTTGGCTCCTGAA |
|  | reverse | AGTCTTCCGCATCATTAGCA |
| Mouse *Ccl20* | forward | GCAAGCGTCTGCTCTTCCTT |
|  | reverse | TTAGGCTGAGGAGGTTCACA |
| Mouse *Ccl21* | forward | GATGATGACTCTGAGCCTCCT |
|  | reverse | TTCTGCACCCAGCCTTCCT |
| Mouse *Ccl22* | forward | TGGCAATTCAGACCTCTGATG |
|  | reverse | TTGCTGGAATGGCAGAAGAA |
| Mouse *Ccl24* | forward | TCATCTTGCTGCACGTCCTTT |
|  | reverse | TAAACCTCGGTGCTATTGCCA |
| Mouse *Ccl25* | forward | TCTCAGGACCAGAAAGGCATT |
|  | reverse | TGGCGGAAGTAGAATCTCACA |
| Mouse *Ccl27* | forward | AGGCTGAGTGAGCATGATGGA |
|  | reverse | TTGGCGTTCTAACCACCGA |
| Mouse *Ccl28* | forward | GCTGTGTGTGTGGCTTTTCAA |
|  | reverse | TACCTCTGAGGCTCTCATCCA |
| Mouse *Cx3cl1* | forward | TGGCTTTGCTCATCCGCTATCAG |
|  | reverse | CGTCTGTGCTGTGTCGTCTCC |
| Mouse *Cxcl1* | forward | ACCCAAACCGAAGTCATAG |
|  | reverse | TTGTATAGTGTTGTCAGAAGC |
| Mouse *CXCL2* | forward | GGTTGACTTCAAGAACATCCAG |
|  | reverse | TTGAGAGTGGCTATGACTTCTG |
| Mouse *Cxcl3* | forward | CAGCCACACTCCAGCCTA |
|  | reverse | CACAACAGCCCCTGTAGC |
| Mouse *Cxcl4* | forward | AGCGATGGAGATCTTAGCTGTGT |
|  | reverse | CCAGGCTGGTGATGTGCTTAA |
| Mouse *Cxcl5* | forward | AGTCAAGAATCATTGGTTGTTAACCTT |
|  | reverse | TCCGGAGACAATGCAATAGTCA |
| Mouse *Cxcl7* | forward | GGAGTTCACTGTGCTGATGTGGA |
|  | reverse | CACAGATGAAGCAGCTGGTCAGTAA |
| Mouse *Cxcl9* | forward | ACAAATCCCTCAAAGACCTCAAACAG |
|  | reverse | ATCTCCGTTCTTCAGTGTAGCAATG |
| Mouse *Cxcl10* | forward | TGAAAGCGTTTAGCCAAAAAAGG |
|  | reverse | AGGGGAGTGATGGAGAGAGG |
| Mouse *Cxcl11* | forward | GTTTCCTGTGAGTCTGCCTTTG |
|  | reverse | AGAGCCAGCCATCCCTACC |
| Mouse *Cxcl12* | forward | CCTCCAAACGCATGCTTCA |
|  | reverse | ACTCTCCTCCCTTCCATTGCA |
| Mouse *Cxcl13* | forward | CAGGCCACGGTATTCTGGA |
|  | reverse | CAGGGGGCGTAACTTGAATC |
| Mouse *Cxcl14* | forward | GCTTCATCAAGTGGTACAAT |
|  | reverse | CTGGCCTGGAGTTTTTCTTTCCAT |
| Mouse *Cxcl15* | forward | CTAGGCATCTTCGTCCGTCC |
|  | reverse | TTGGGCCAACAGTAGCCTTC |
| Mouse *Cxcl16* | forward | AAACATTTGCCTCAAGCCAGT |
|  | reverse | GTTTCTCATTTGCCTCAGCCT |
| Mouse *Cxcl17* | forward | ATGAAGCTTCTAGCCTCTCCC |
|  | reverse | CTATAAGGGCAGCGCAAAGCTTGC |
| Mouse *Cd25* | forward | AACACCACCGATTTCTGG |
|  | reverse | TCTCCGTCATTGCAGTTG |
| Mouse *Foxp3* | forward | GGCAGGCAACAACTCAGTC |
|  | reverse | CAGGCACACTCCAACACATAA |
| Mouse *Cldn1* | forward | GCTGGGTTTCATCCTGGCTTCTC |
|  | reverse | CCTGAGCGGTCACGATGTTGTC |
| Mouse *Ly6g* | forward | TCATCCTTCTTGTGGTCCTA |
|  | reverse | CCAGAGCAACGCAAAATC |
| Human *GAPDH* | forward | ACCCAGAAGACTGTGGATGG |
|  | reverse | CAGTGAGCTTCCCGTTCAG |
| Human *ANGPTL4* | forward | TGGTTTGGCACCTGCAGCCATTC |
|  | reverse | TGCTGCCATGGGCTGGATCAAC |
| Human *CCL5* | forward | GTGTGCCAACCCAGAGA |
|  | reverse | GGATAGTGAGGGGAAGCC |
| Human *CXCL1* | forward | TTTTGAAATGTCAACCCCAAG |
|  | reverse | GATCTCATTGGCCATTTGCT |
| Human *CLDN1* | forward | CCGTGCCTTGATGGTGGTTGG |
|  | reverse | CATCTTCTGCACCTCATCGTCTTCC |
| Human *IL17A* | forward | GAGATATCCCTCTGTGATCTGG |
|  | reverse | GACAGAGTTCATGTGGTAGTCC |
| Human *Foxp3* | forward | AAGGAGGATGGACGAACA |
|  | reverse | CTGGTTGTGAAGGCTCTG |
| Human *CD25* | forward | GAGGAAGAGTAGAAGAACAATC |
|  | reverse | GCTGGCATAGAGACAAGG |
| Human *CLDN2* | forward | CGGTAGCAGGTGGAGTCTT |
|  | reverse | TTGGTAGGCATCGTAGTAGTTG |
| Human *CLDN4* | forward | CCTTCATCGGCAGCAACATTGTC |
|  | reverse | AGCAGCGAGTCGTACACCTTG |
| Human *CLDN6* | forward | TCATCGGCAACAGCATCGT |
|  | reverse | CCAGCAAGGTAGACCAGCAA |
| Human *CLDN7* | forward | GGTCTTGCCGCCTTGGTAGC |
|  | reverse | GGACAGGAACAGGAGAGCAGTG |
| Human *CLDN12* | forward | AACGAGAAGAACCTGACTGTT |
|  | reverse | GCACTATTGACCAGACACTTG |
| Human *CLDN23* | forward | CAGCCAGTGGACGTGGAGTTG |
|  | reverse | CAGCGAGGTGACCATGAGTGC |
| Human *ZO1* | forward | CGGTCCTCTGAGCCTGTAAG |
|  | reverse | GGATCTACATGCGACGACAA |
| Human *OCLN* | forward | CTATAAATCCACGCCGGTTC |
|  | reverse | TATTCCTGTAGGCCAGTGTC |

For the probes, a FAM fluorescent reporter is coupled to the 5' end, and a TAMRA quencher is coupled to the 3' end.

Table. S4. siRNAs used in the present study

| Name |  | Sequence 5′→3′ |
| --- | --- | --- |
| *ANGPTL4* siRNA | sense | CCACAAGCACCUAGACCAUTT |
|  | antisense | AUGGUCUAGGUGCUUGUGGTT |

Table. S5. Primers for Chromatin Immunoprecipitation (ChIP) PCR analysis

| Gene | Primer | Sequence 5′→3′ |
| --- | --- | --- |
| *ANGPTL4* | forward | CTGCCTGAGCCTGGAGCG |
|  | reverse | TGGGCGGAGGAGTCTTGG |

Primers for ChIP analysis of ANGPTL4 were designed and produced by Sangon Biotech (Shanghai, China)

Table. S6. Transcription factors in the *ANGPTL4*/*Angptl4* promoter sequence (PROMO)

| Transcription factors | | | |
| --- | --- | --- | --- |
| HOMO | | MUS | |
|  | RXR-alpha |  | HOXA5 |
|  | RAR-beta |  | COE1 |
|  | C/EBPbeta |  | c-Fos |
|  | HNF-3alpha |  | C/EBPbeta |
|  | GR-beta |  | TFE3-S |
|  | TFIID |  | GR |
|  | GR |  | myogenin |
|  | c-Jun |  | MyoD |
|  | GR-alpha |  | NF-AT4 |
|  | USF2 |  | YY1 |
|  | AP-2alphaA |  | C/EBPalpha |
|  | VDR |  | RXR-alpha |
|  | PXR-1:RXR-alpha |  | JunD |
|  | C/EBPalpha |  | c-Jun |
|  | TFII-I |  | JunB |
|  | IRF-1 |  | GATA-3 |
|  | YY1 |  | GATA-2 |
|  | HOXD9 |  | Nkx2-1 |
|  | HOXD10 |  | HES-1 |
|  | XBP-1 |  | NF-1 |
|  | Ik-1 |  | Tal-1 |
|  | LEF-1 |  | AP-1 |
|  | NF-1 |  | POU2F2 |
|  | PEA3 |  | POU2F2 (Oct-2.1) |
|  | PPAR-alpha:RXR-alpha |  | POU2F2 (Oct-2.3) |
|  | c-Myb |  | POU2F2 (Oct-2.4) |
|  | Pax-5 |  | POU2F2 (Oct-2.6) |
|  | p53 |  | USF-1 |
|  | AR |  | PU.1 |
|  | ER-alpha |  | CRE-BP2 |
|  | STAT4 |  | NF-kappaB |
|  | c-Ets-1 |  | TCF-1(P) |
|  | MAZ |  | NF-muNR |
|  | ENKTF-1 |  | f(alpha)-f(epsilon) |
|  | NFI/CTF |  | AhR |
|  | HIF-1 |  | Pax-5 |
|  | CREB |  | Sp1 |
|  | ATF-2 |  | RelA |
|  | AP-1 |  | NF-kappaB1 |
|  | c-Fos |  | NF-AT1 |
|  | Elk-1 |  | E2F-1 |
|  | PR B |  | CP2 |
|  | PR A |  | HNF-3 |
|  | GATA-2 |  | HNF-3beta |
|  | GATA-1 |  | c-Rel |
|  | c-Ets-2 |  | MTF-1 |
|  | T3R-beta1 |  |  |
|  | c-Myc |  |  |
|  | USF1 |  |  |
|  | Sp1 |  |  |
|  | ETF |  |  |
|  | GCF |  |  |
|  | IRF-2 |  |  |
|  | TCF-4E |  |  |
|  | E2F-1 |  |  |
|  | EBF |  |  |
|  | NF-AT1 |  |  |
|  | ATF-1 |  |  |
|  | NF-kappaB1 |  |  |
|  | TBP |  |  |
|  | FOXP3 |  |  |
|  | WT1 |  |  |
|  | STAT1beta |  |  |
|  | RAR-beta:RXR-alpha |  |  |
|  | TCF-4 |  |  |
|  | AhR |  |  |
|  | SRY |  |  |
|  | HMG I(Y) |  |  |
|  | NF-kappaB |  |  |
|  | RelA |  |  |

*Set the maximum matrix dissimilarity rate to 9%.

*The transcription factors labeled in red are found in both humans and mice.

Table. S7. Potential binding sites of NF-κB1 in the *ANGPTL4*/*Angptl4* promoter sequence (JASPAR)

| HOMO | Predicted sequence | | Position | Relative score | MUC | Predicted sequence | | Position | Relative score |
| --- | --- | --- | --- | --- | --- | --- | --- | --- | --- |
| NF-κB1 (MA0105.4) | 1 | AGGGCATCCCCCA | -157；-145 | 0.843 | NF-κB1 (MA0105.1) | 1 | GGGAATTTCC | -254；-245 | 0.999 |
|  | 2 | AGGTGATCCGCCC | -1345；-1333 | 0.795 |  | 2 | GGGAATTAAC | -1472；-1463 | 0.857 |
|  | 3 | TGGGGTATCTCCA | -1703；-1691 | 0.777 |  | 3 | GGGGTTTTTC | -1917；-1908 | 0.846 |
|  | 4 | AGGGAAACTCCAT | -2822；-2810 | 0.762 |  | 4 | GGTAAATTCC | -764；-755 | 0.845 |
|  | 5 | CGAGGTTTCACCA | -1391；-1379 | 0.736 |  | 5 | GGGGAGTCCT | -2602；-2593 | 0.829 |
|  | 6 | AAGCGATTCTCCT | -1473；-1461 | 0.733 |  | 6 | GGAACTTCAC | -1167；-1158 | 0.828 |
|  | 7 | TGGTGAAACCCTG | -2680；-2668 | 0.731 |  | 7 | GGGAAATCTT | -1635；-1626 | 0.779 |
|  | 8 | AGTGAAACCCCGT | -2990；-2978 | 0.727 |  | 8 | GGGTCTTCCA | -2800；-2791 | 0.773 |
|  | 9 | TGGGGTTTTCCTC | -2115；-2103 | 0.720 |  | 9 | TGGAGATCCC | -2977；-2968 | 0.768 |
|  | 10 | TGGAGAATCGCTT | -2901；-2889 | 0.711 |  | 10 | GTGAATCACC | -2926；-2917 | 0.746 |
|  | 11 | CGGGAGAGCCACA | -183；-171 | 0.700 |  | 11 | GGGGTTCCGC | -529；-520 | 0.745 |
|  |  |  |  |  |  | 12 | AGGGTTTTCT | -2087；-2078 | 0.743 |
| RELA (MA0107.1) | 1 | GGGGTTTTCC | -2114；-2105 | 0.921 |  | 13 | GTGGTTTTTC | -2222；-2213 | 0.732 |
|  | 2 | GGGAAACTCC | -2821；-2812 | 0.826 |  | 14 | GGGAACTGTC | -1851；-1842 | 0.732 |
|  | 3 | GGGTATCTCC | -1701；-1692 | 0.799 |  | 15 | CAGAAATCCC | -680；-671 | 0.728 |
|  | 4 | AGGGATTTGC | -2074；-2065 | 0.784 |  | 16 | GATGAATTCC | -847；-838 | 0.711 |
|  | 5 | GGTGAGTTCC | -793；-784 | 0.771 |  | 17 | GGGTCTTCTT | -1891；-1882 | 0.709 |
|  | 6 | CGGAGATTGC | -2860；-2851 | 0.764 |  | 18 | GAGAACTTTC | -423；-414 | 0.709 |
|  | 7 | GGGGGCTTGC | -285；-276 | 0.741 |  | 19 | TGGACCTTCT | -423；-414 | 0.708 |
|  | 8 | TGCAATTTCA | -278；-269 | 0.741 |  |  |  |  |  |
|  | 9 | GGGCATCCCC | -156；-147 | 0.734 |  |  |  |  |  |
|  | 10 | CTGGGTTTCA | -375；-366 | 0.731 |  |  |  |  |  |
|  | 11 | TGGGATAGCC | -2211；-2202 | 0.730 |  |  |  |  |  |
|  | 12 | TGGGATTACA | -1297；-1288 | 0.730 |  |  |  |  |  |
|  | 13 | GCGATTCTCC | -1471；-1462 | 0.720 |  |  |  |  |  |
|  | 14 | TGGAGCTTGC | -2565；-2556 | 0.715 |  |  |  |  |  |
|  | 15 | TGCAGTTTCA | -1994；-1985 | 0.711 |  |  |  |  |  |
|  | 16 | CGAGGTTTCA | -1391；-1382 | 0.704 |  |  |  |  |  |

*Total putative sites were predicted with relative profile score threshold 70%.

Table. S8. Potential binding sites of NF-κB1 in the *ANGPTL4*/*Angptl4* promoter sequence (PROMO)

| **HOMO** | Predicted sequence | | Position | | Dissimilarity | | RE equally | | RE query | **MUC** | Predicted sequence | | Position | Dissimilarity | RE  equally | RE  query |
| --- | --- | --- | --- | --- | --- | --- | --- | --- | --- | --- | --- | --- | --- | --- | --- | --- |
| NF-κB1 | 1 | AGGGCATCCCC | -158；-148 | 3.230809 | | 0.025 | | | 0.033 | NF-κB1 | 1 | CTGGGAATTTCC | -257；-246 | 8.247013 | 0.02146 | 0.019 |
|  |  |  |  | |  | |  |  | |  |  |  |  |  |  |  |
|  |  |  |  | |  | |  |  | | REL A | 1 | GGGAATTTCC | -255；-246 | 0 | 0.00286 | 0.003 |
|  |  |  |  | |  | |  |  | |  | 2 | GGGAATTAAC | -1473；-1464 | 3.613792 | 0.04864 | 0.043 |
|  |  |  |  | |  | |  |  | |  | 3 | GGGAACTGTC | -1852；-1843 | 5.083279 | 0.0658 | 0.060 |

*Set the maximum matrix dissimilarity rate to 9%.
